# *Rickettsia* Phylogenomics: Unwinding the Intricacies of Obligate Intracellular Life

**DOI:** 10.1371/journal.pone.0002018

**Published:** 2008-04-16

**Authors:** Joseph J. Gillespie, Kelly Williams, Maulik Shukla, Eric E. Snyder, Eric K. Nordberg, Shane M. Ceraul, Chitti Dharmanolla, Daphne Rainey, Jeetendra Soneja, Joshua M. Shallom, Nataraj Dongre Vishnubhat, Rebecca Wattam, Anjan Purkayastha, Michael Czar, Oswald Crasta, Joao C. Setubal, Abdu F. Azad, Bruno S. Sobral

**Affiliations:** 1 Virginia Bioinformatics Institute at Virginia Tech, Blacksburg, Vigrinia, United States of America; 2 Department of Microbiology and Immunology, University of Maryland School of Medicine, Baltimore, Maryland, United States of America; Columbia University, United States of America

## Abstract

**Background:**

Completed genome sequences are rapidly increasing for *Rickettsia*, obligate intracellular α-proteobacteria responsible for various human diseases, including epidemic typhus and Rocky Mountain spotted fever. In light of phylogeny, the establishment of orthologous groups (OGs) of open reading frames (ORFs) will distinguish the core rickettsial genes and other group specific genes (class 1 OGs or C1OGs) from those distributed indiscriminately throughout the rickettsial tree (class 2 OG or C2OGs).

**Methodology/Principal Findings:**

We present 1823 representative (no gene duplications) and 259 non-representative (at least one gene duplication) rickettsial OGs. While the highly reductive (∼1.2 MB) *Rickettsia* genomes range in predicted ORFs from 872 to 1512, a core of 752 OGs was identified, depicting the essential *Rickettsia* genes. Unsurprisingly, this core lacks many metabolic genes, reflecting the dependence on host resources for growth and survival. Additionally, we bolster our recent reclassification of *Rickettsia* by identifying OGs that define the AG (ancestral group), TG (typhus group), TRG (transitional group), and SFG (spotted fever group) rickettsiae. OGs for insect-associated species, tick-associated species and species that harbor plasmids were also predicted. Through superimposition of all OGs over robust phylogeny estimation, we discern between C1OGs and C2OGs, the latter depicting genes either decaying from the conserved C1OGs or acquired laterally. Finally, scrutiny of non-representative OGs revealed high levels of split genes versus gene duplications, with both phenomena confounding gene orthology assignment. Interestingly, non-representative OGs, as well as OGs comprised of several gene families typically involved in microbial pathogenicity and/or the acquisition of virulence factors, fall predominantly within C2OG distributions.

**Conclusion/Significance:**

Collectively, we determined the relative conservation and distribution of 14354 predicted ORFs from 10 rickettsial genomes across robust phylogeny estimation. The data, available at PATRIC (PathoSystems Resource Integration Center), provide novel information for unwinding the intricacies associated with *Rickettsia* pathogenesis, expanding the range of potential diagnostic, vaccine and therapeutic targets.

## Introduction

Rickettsiae are a group of organisms belonging to the class *Alphaproteobacteria*, a large and metabolically diverse group of gram-negative bacteria [1]–[3]. Within *Alphaproteobacteria*, the order Rickettsiales comprises three families: Holosporaceae, Anaplasmataceae and Rickettsiaceae [4], of which *Rickettsia* spp. are grouped in the latter, along with the monotypic genus *Orientia*, the scrub typhus agent [5]. Robust phylogenetic analysis further suggests that the abundant free-living marine bacterioplankton *Pelagibacter ubique* and mitochondria are early-branching groups of the order [6]. Species in the genus *Rickettsia* are obligate intracellular symbionts of plants [7], amoebae [8], [9], arthropods [e.g., 10]–[13], annelids [14], vertebrates [15] and likely many other organisms [16]. Most *Rickettsia*-containing vertebrates are secondary hosts that acquired these bacteria via blood-feeding arthropods or the transdermal inoculation or inhalation of the feces of infected arthropods. *Rickettsia* spp. are often parasitic in the secondary vertebrate host [e.g., 17], and their pathogenicity to some extent has been well studied. In particular, human rickettsial infections are known to cause many diseases, including epidemic typhus (*R*. *prowazekii*), murine typhus (*R*. *typhi*), murine typhus-like (*R*. *felis*), rickettsial pox (*R*. *akari*), Rocky Mountain spotted fever (*R*. *rickettsii*), Boutonneuse fever (*R*. *conorii*), and North Asian tick typhus (*R*. *sibirica*). These virulent species of rickettsiae are of great interest both as emerging infectious diseases [18] and for their potential deployment as bioterrorism agents [19], [20].

**Figure 1:**
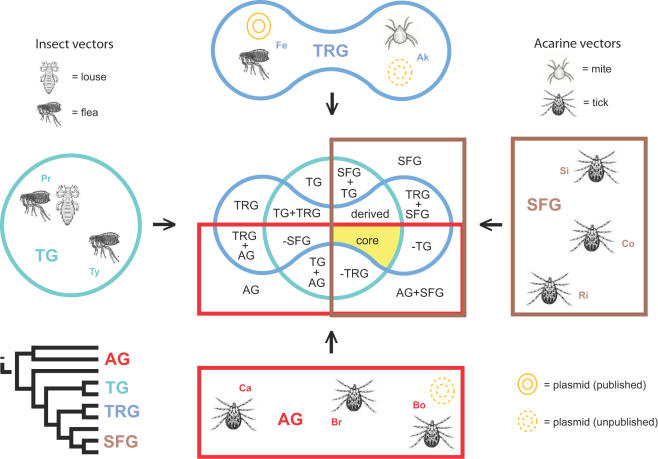
Venn diagram depicting 15 intersections for the four rickettsial groups. Classification scheme based on molecular phylogeny estimation [28], the topology of which is shown in the lower left; AG = ancestral group, TG = typhus group, TRG = transitional group, SFG = spotted fever group. Genome codes are as follows: Br = *R*. *bellii* str. RML369-C, Bo = *R*. *bellii* str. OSU 85 389, Ca = *R*. *canadensis* str. McKiel, Pr = *R*. *prowazekii* str. Madrid E, Ty = *R*. *typhi* str. Wilmington, Ak = *R*. *akari* str. Hartford, Fe = *R*. *felis* str. URRWXCal2, Ri = *R*. *rickettsii* str. Sheila Smith CWPP, Co = *R*. *conorii* str. Malish 7, and Si = *R*. *sibirica* str. 246. Arthropod hosts are illustrated for each genome, and strains known to harbor plasmids are depicted.

Due to both small genome size and medical importance, ten genome sequences from *Rickettsia* spp. have been published and annotated in the last decade [9], [21]–[27], providing a foundation to study the evolutionary history of these lineages through comparative genomics. Recently, Gillespie et al. [28] proposed a revision to the long-standing classification of *Rickettsia* by erecting the transitional group (TRG) as a distinct lineage that shares immediate ancestry with the members of the spotted fever group (SFG) rickettsiae. Coupled with the typhus group (TG) and ancestral group (AG) rickettsiae, these four rickettsial lineages comprising 10 sequenced genomes present an opportunity to create a database that encompasses the distribution of the predicted open reading frames (ORFs) across all ten annotated genomes (**Figure 1**).

Establishing orthology across multiple genomes serves not only to identify genes with shared evolutionarily histories, but also facilitates genome annotation [29], [30], and significant attention has focused on algorithms for creating orthologous groups (OGs). Recent work has centered on the following four aspects: i) overall improvement of OG assignment in the face of paralogy, ii) building tools for the cross-querying of taxon-specific databases, iii) creating databases that house specific gene or protein profiles for facilitating the identification of orthologs in novel sequences, and iv) the inclusion of phylogeny estimation into the processes of assigning orthology and detecting paralogy.

At the PathoSystems Resource Integration Center (PATRIC) [31], OGs have been preliminarily established for several groups of organisms, including *Rickettsia* spp. The advantage of a *Rickettsia*-specific database lies not only in the ability to query exclusively against the 10 genomes currently annotated in our system, but also to evaluate the results of several algorithmic approaches that create OGs. Furthermore, PATRIC offers continued updates to the annotation of rickettsial genes and proteins, and provides multiple sequence alignments as well as phylogenetic trees, when applicable, for each OG consisting of two to ten rickettsial taxa. The database will continually evolve with the addition of newly sequenced rickettsial genomes, with existing OG assignments driving the curation process of raw genome data.

In the present study, we report the rickettsial OGs (RiOGs) in conjunction with a highly robust phylogeny of the core rickettsial genes, providing an evolutionary framework for interpreting the genomic characteristics of the four main lineages of *Rickettsia*. These data highlight the genetic anomalies previously characterized for this genus, such as extremely reduced genomes and the high presence of putative pseudogenes, and also reveal novel characteristics including the lack of group-specific virulence factors and high occurrence of lateral transfer between groups that harbor plasmids (AG and TRG rickettsiae). Information on the conserved core genes, as well as those that may be involved in specific functions that define monophyletic groups, host associations, and plasmid-related behavior, will be valuable resources for future laboratory work (e.g., development of vaccines, diagnostics and therapeutics) as well as further evolutionary studies of this intriguing obligate intracellular bacterial group.

## Results and Discussion

### Synteny and Phylogeny of *Rickettsia* Genomes

**Figure 2:**
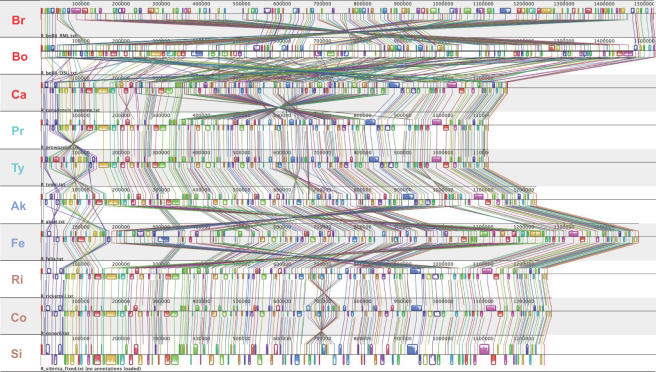
Alignment of 10 rickettsial genomes. Taxa are in the same position as in estimated trees in Figure 3, with taxon abbreviations explained in the Figure 1 legend. Alignment created using Mauve [189] after reindexing the *R*. *sibirica* genome (see text for details).

Whole genome alignments for the ten analyzed *Rickettsia* taxa reveal highly conserved colinearity in six of the seven derived species (sans *R*. *bellii* and *R*. *canadensis*) with minimal gene rearrangements, most of which occur near the predicted origin of replication termination (**Figure 2**). However, the *R*. *felis* genome contains several long-range symmetrical inversions in the central region of the alignment that are not found in other taxa. Removal of *R*. *felis* from the alignment illustrates the highly conserved synteny across the derived rickettsia taxa (**Figure S1-A**). Furthermore, switching the positions of *R*. *akari* and *R*. *felis* in the alignment (**Figure S1-B**) demonstrates that these central inversions in *R*. *felis*, as well as a large genome size, are autapomorphic (uniquely derived) traits within derived rickettsiae. Among the three AG rickettsiae, *R*. *canadensis* (formerly *R*. *canada*) is more colinear with the derived taxa than it is to either *R*. *bellii* strain. Like *R*. *felis*, *R*. *canadensis* contains several autapomorphic symmetrical inversions in the central region of the alignment, yet they are smaller than the long-range inversions found in *R*. *felis*. As previously reported [32], *R*. *bellii* str. RML369-C shares little colinearity with other rickettsial genomes, and our analysis of both *R*. *bellii* genomes is in agreement with this observation. Despite several long and short range inversions between the *R*. *bellii* str. RML369-C and *R*. *bellii* str. OSU 85-389 genomes, few gene positions are shared with *R*. *bellii* and *R*. *canadensis* or the derived taxa (**Figure 2**), and switching the positions of the *R*. *bellii* strains in the alignment does not result in more conserved synteny between either strain and the derived taxa (**Figure S1-C, D**).

**Figure 3:**
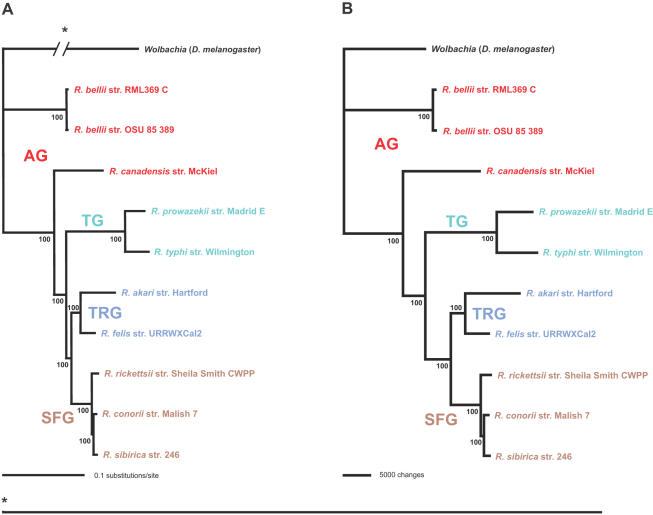
Estimated phylogenies of ten rickettsial taxa based on 731 representative core proteins. (A) Tree from Bayesian analysis. Three MCMC chains were primed with a neighbor-joining tree and run independently for 25000 generations in model-jumping mode. Burn-in was attained by 2500 generations for all chains, and a single tree topology with exclusive use of the Jones substitution model was observed in post burn-in data. The consensus tree shown here thus has 100% support for every branch. Branch support is from the distribution of posterior probabilities from all trees minus the burn-in. (B) Tree from exhaustive search using parsimony. Branch support is from one million bootstrap replicates.

Phylogenetic analyses implementing both maximum likelihood and parsimony of the 731 representative core rickettsial proteins (discussed below) resulted in robust estimates for these 10 taxa (**Figure 3**). The estimated tree topologies are identical in branching pattern and are congruent with the tree from our previous analysis of 716 fewer genes [28], suggesting that ten or more concatenated (and well-behaved, with high signal to noise ratio) genes are sufficient for obtaining a robust phylogenetic estimate for these rickettsial taxa. Thus, our recent classification scheme for *Rickettsia* consisting of 4 major groups (AG rickettsiae: *R*. *bellii* str. RML369-C, *R*. *bellii* str. OSU 85 389, *R*. *canadensis* str. McKiel; TG rickettsiae: *R*. *prowazekii* str. Madrid E, *R*. *typhi* str. Wilmington; TRG rickettsiae: *R*. *akari* str. Hartford, *R*. *felis* str. URRWXCal2; SFG rickettsiae: *R*. *rickettsii* str. Sheila Smith CWPP, *R*. *conorii* str. Malish 7, *R*. *sibirica* str. 246) is substantiated with a phylogenomic approach. In what follows, we use this evolutionary framework to analyze the distribution and relative conservation of all predicted genes for these ten rickettsial genomes.

### Predicted OGs: Conservation and Representation

**Figure 4:**
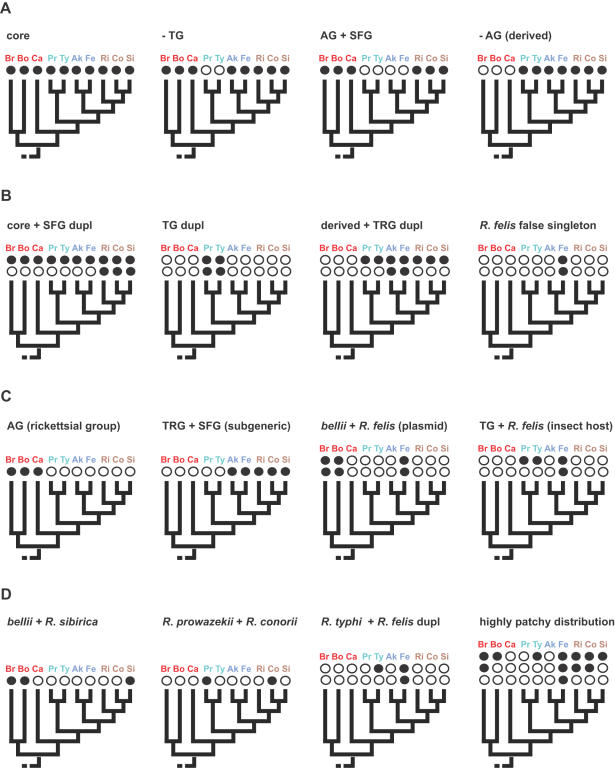
Illustration of representative and non-representative OGs and their categorization into Class 1 and Class 2 OGs. Taxon abbreviations are explained in the Figure 1 legend. Dark circles depict gene presence, while open circles depict gene absence. (A) Representative OGs: orthologous groups with only one ORF per included genome. Our analysis includes ten rickettsial genomes, thus representative OGs only include from 2–10 ORFs. Four examples are shown. (B) Non-representative OGs: orthologous groups with multiple ORFs from at least one included genome, comprised of either recent (orthologs) or distant (paralogs) gene duplications (dupl). False singleton OGs are comprised of only one taxon, but with multiple ORFs from that taxon (example on right). Four examples are shown. (C) Class 1 OGs (C1OGs): orthologous groups comprising single rickettsial groups (e.g., AG, TG, TRG, and SFG), shared rickettsial groups (subgeneric), plasmid-harboring genomes, and genomes with common arthropod hosts. Two representative (left) and two non-representative (right) C1OGs are shown. (D) Class 2 OGs (C2OGs): orthologous groups with patchy distribution across the rickettsial tree, depicting gene losses and/or genes acquired laterally. Two representative and two non-representative C2OGs are shown.

In the analysis of the rapidly growing list of rickettsial genomes we determined that OrthoMCL, a program that applies the Markov clustering algorithm of Van Dongen [33] to resolve the many-to-many orthologous relationships present within cross genome comparisons [34], outperformed more traditional approaches to establishing OGs, such as bidirectional best BLAST hits with and without cliques. Thus, we show here the results generated by OrthoMCL only, which grouped 12887 ORFs into 2082 total OGs (**Table 1**). The bulk (88%) of these OGs are representative (**Figure 4A**), meaning they include only one CDS per strain, thus ranging in membership from 2–10 sequences. The remaining 12% of the OGs are non-representative (**Figure 4B**) and include multiple predicted ORFs from at least one member. Categorization of the OGs into two classes based on distribution across the rickettsial tree and other attributes, such as presence of plasmids and common arthropod hosts (**Figure 4C–D**), reveals that 69% of the OGs are comprised of single rickettsial groups (e.g., AG, TG, TRG, and SFG), shared rickettsial groups (subgeneric), plasmid-harboring genomes, and genomes with common arthropod hosts (**Table 1**). These class 1 OGs (C1OGs) contain 76% of the predicted ORFs grouped into OGs by OrthoMCL, suggesting that our criteria for distinguishing biologically interesting protein families based empirically on robust phylogeny estimation, presence of extra-chromosomal DNA and shared arthropod hosts is valid. The remaining ORFs grouped into class 2 OGs (C2OGs) depict gene families drifting or sporadically lost from the core genetic repertoire of the rickettsial ancestor [32] or genes acquired laterally (**Figure S2**). Interestingly, while the majority (71%) of representative OGs qualify as C1OGs, the non-representative OGs are distributed within C1OGs and C2OGs in near equal frequency (**Table 1**), suggesting minimal conservation for gene duplications and laterally acquired genes in these rickettsial genomes.

**Table 1:** Distribution of representative and non-representative OGs predicted across 14354 ORFs from ten rickettsial genomes, and their categorization into Class 1 and Class 2 OGs.^1^

Composition^2^	All OGs		C1OGs^3^		C2OGs^4^	
	No. OGs	No. ORFs	No. OGs	No. ORFs	No. OGs	No. ORFs
representative	1823 (88%)	11026 (86%)	1300 (71%)	8910 (81%)	523 (29%)	2116 (19%)
non-representative	259 (12%)	1861 (14%)	145 (56%)	930 (50%)	114 (44%)	931 (50%)
**Tot.**	**2082**	**12887**	**1445 (69%)**	**9840 (76%)**	**637 (31%)**	**3047 (24%)**

1 Of 14354 total ORFs, 12887 were grouped by OrthoMCL, leaving 1467 singletons.

2 Containing either no duplications per each member within an OG (representative) or at least one member with a duplication within an OG (non-representative).

**Figure 5:**
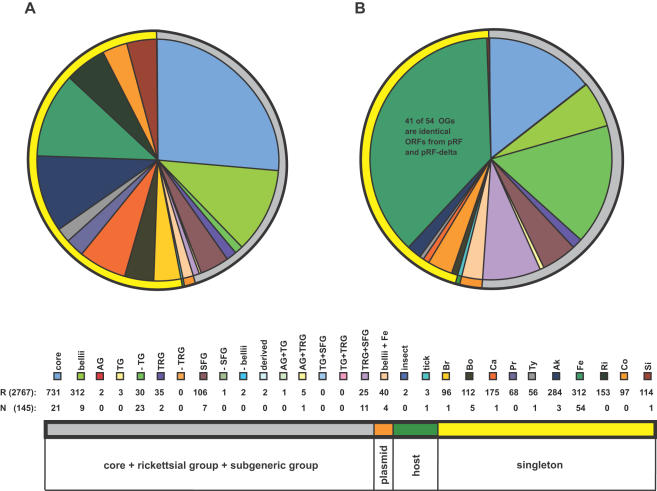
Comparison of the distributions of 1300 representative and 145 non-representative class 1 OGs (C1OGs), 66 false singletons, and 1467 singleton ORFs. Slices depict 16 generic and subgeneric groups, false singletons, singletons, plasmid associated groups, and two host-related groups, with outer circle colors depicted in schema. Taxon abbreviations, including subgeneric groups, are explained in the Figure 1 legend. (A) Distribution of 1300 representative C1OGs and 1467 singletons. (B) Distribution of 79 non-representative C1OGs and 66 false singletons.

3 Class 1 OGs (see **Figure 4** for description and **Figure** **5** and **Figure 7** for distribution of representative and non-representative C1OGs across rickettsial phylogeny).

4 Class 2 OGs (see **Figure 4** for description and **Figure S2** for distribution of representative and non-representative C2OGs across rickettsial phylogeny).

The RiOGs range in membership from two to 31 ORFs, with few (<3%) OGs exceeding more than 10 ORFs (**Table 2**). Representative C1OGs comprise a substantial portion (64%) of the OGs with membership of 10 or fewer ORFs. Regarding the OGs with more than 10 members, a range from 4% (*R*. *prowazekii*) to 32% (*R*. *conorii*) illustrates the frequencies at which a particular rickettsial genome contributes to non-representation. As expected due to their smaller genome sizes and few gene duplications [21], [25], TG rickettsiae make little contribution (avg. 5%) to larger non-representative OGs as compared to AG (avg. 19%), TRG (avg. 17%) and SFG (avg. 31%) rickettsiae (**Table 2**). Thus, these three latter groups have genomes more tolerant of multicopy genes, particularly those resulting from transposases and other insertion sequences, which act to produce elevated levels of paralogous genes. For instance, analysis of the distribution of RiOGs containing genes associated with mobile DNA and/or horizontal gene transfer (HGT), such as genes coding for proteins with ankyrin (ANK) and tetratricopeptide repeat (TPR) motifs, proteins with rickettsial palindromic elements (RPE), proteins associated with transposable elements (TNP), proteins of toxin-antitoxin modules (TA), and phage related elements, revealed that they are nearly non-existent in TG rickettsial genomes (**Table 3**). The remaining three lineages, all purportedly containing some species that harbor plasmids, have elevated levels of most of these gene groups compared to TG rickettsiae. Interestingly, nearly half (47%) of the C2OGs are comprised of these six gene groups, while only a small portion of the C1OGs (5%) and singletons (4%) contain them (**Table 3**). Given the probable lateral inheritance of many of these genes, either as facilitators or products of HGT, it is evident that they are less conserved and of less importance to overall rickettsial fitness and survival. However, their contribution to species- and strain-specific pathogenicity cannot be overlooked. Interestingly, our observation that these more promiscuous gene families tend to occur predominantly within C2OGs is congruent with a recent study demonstrating that barriers to bacterial HGT are more stringent for single copy genes [35].

**Table 2:** Breakdown of membership (no. ORFs) across 2082 rickettsial OGs.

OGs with 10 or fewer ORFs			
No. ORFs	No. OGs	Representative C1OGs^1^	Remaining OGs^2^^,^ 3 ^,^ 4
2	585	312 (*bellii*); 3 (TG); 35 (TRG); 40 (*bellii*+Fe)	195
3	225	2 (AG); 106 (SFG); 2 (insect)	115
4	128	0 (TG+TRG)	128
5	90	25 (TRG+SFG); 1 (AG+TG); 5 (AG+TRG); 0 (TG+SFG)	59
6	62	3 (tick)	59
7	65	2 (derived); 1 (-SFG)	62
8	65	2 (-*bellii*); 30 (-TG); 0 (-TRG)	33
9	56	0	56
10	748	731 (core)	17
**Tot**	**2024**	**1300**	**724 (523 rep., 201 non-rep.)**

Ogs with 11 or more members (all non-representative)																					
No. ORFs	No. OGs	Distribution^4^^–^ [Table-fn nt110] 6																			
		Br		Bo		Ca		Pr		Ty		Ak		Fe		Ri		Co		Si	
11	24	23	0	31	7	23	3	16	0	18	2	23	2	31	6	31	8	33	8	34	9
12	14	15	4	15	3	11	3	7	1	7	0	42	8	17	5	17	5	19	6	18	5
13	6	9	3	7	1	7	2	2	0	2	0	11	4	7	1	11	5	11	5	11	5
14	5	8	3	6	1	6	3	1	0	1	0	10	3	7	1	9	3	12	5	10	4
15	3	15	2	19	3	1	0	1	0	1	0	0	0	1	0	2	1	3	1	2	1
17	1	0	0	0	0	0	0	0	0	0	0	0	0	17	1	0	0	0	0	0	0
18	3	2	0	22	3	2	0	0	0	1	0	6	2	2	0	8	2	7	2	8	2
23	1	1	0	2	1	1	0	1	0	0	0	3	1	1	0	4	1	4	1	5	1
31	1	2	1	1	0	0	0	0	0	0	0	1	0	27	1	0	0	0	0	0	0
**Tot**	**58**	**75**	**13**	**103**	**19**	**51**	**11**	**28**	**1**	**30**	**2**	**96**	**20**	**110**	**15**	**82**	**25**	**89**	**28**	**88**	**27**
**% NR**		**17**		**19**		**22**		**4**		**7**		**21**		**14**		**31**		**32**		**31**	

1 C1OGs (see **Figure 4** for description and **Figure 5** and **Figure 7** for distribution of representative and non-representative

C1OGs across rickettsial phylogeny).

2 Comprising both representative and non-representative OGs.

3 Includes some non-representative C1OGs, which are shown in **Figure 5** and **Figure 7** **.**

4 Distributions of included C2OGs are shown over rickettsial phylogeny in **Figure S2**

5 First number is total no. ORFs within OGs; second number depicts no. of ORFs causing non-representation.

6 Taxon abbreviations are explained in the **Figure 1** legend.

**Table 3:** Distribution across 10 rickettsial genomes of OGs and singletons containing proteins with ankyrin (ANK) and tetratricopeptide repeat (TPR) motifs, proteins with rickettsial palindromic elements (RPE), proteins associated with transposable elements (TPN), proteins of toxin-antitoxin modules (TA), and phage related proteins.

C1OGs^1^	Tot. OGs		Distribution^2^														
			ANK		TPR		RPE		TNP		TA		PHAGE		Tot.		
	R	NR	R	NR	R	NR	R	NR	R	NR	R	NR	R	NR	R	NR	ALL
**core**	731	21	0	0	1	0	10	0	0	0	0	0	0	0	11	0	11
**AG**	2	0	0	0	0	0	0	0	0	0	0	0	0	0	0	0	0
***bellii***	312	9	10	0	3	0	0	0	3	5	1	0	1	0	18	5	23
**-** ***bellii***	2	0	0	0	0	0	0	0	0	0	0	0	0	0	0	0	0
**TG**	3	0	0	0	0	0	0	0	0	0	0	0	0	0	0	0	0
**-TG**	30	23	0	0	0	1	1	0	1	0	1	0	0	2	3	3	6
**TRG**	35	2	1	0	0	0	0	0	0	0	4	0	0	0	5	0	5
**SFG**	106	7	4	0	0	0	2	0	1	0	0	0	0	0	7	0	7
**-SFG**	1	0	0	0	0	0	0	0	0	0	0	0	0	0	0	0	0
**derived**	2	0	0	0	0	0	0	0	0	0	0	0	0	0	0	0	0
**AG+TG**	1	0	0	0	0	0	0	0	0	0	0	0	0	0	0	0	0
**AG+TRG**	5	1	0	0	0	0	0	0	1	0	0	0	0	0	1	0	1
**TRG+SFG**	25	11	0	0	0	1	0	0	0	0	3	0	0	0	3	1	4
***bellii*** **+Fe**	40	4	1	0	0	0	0	0	0	0	5	0	1	0	7	0	7
**insect**	2	0	0	0	0	0	0	0	0	0	0	0	0	0	0	0	0
**tick**	3	1	0	0	0	0	0	0	0	0	0	0	0	0	0	0	0
**Tot.**	**1300**	**79**	**16**	**0**	**4**	**2**	**13**	**0**	**6**	**5**	**14**	**0**	**2**	**2**	**55**	**9**	**64**
																	**(5%)**

C2OGs^3^	% of OGs^4^	Distribution^2^														
		ANK		TPR		RPE		TNP		TA		PHAGE		Tot.		
		R	NR	R	NR	R	NR	R	NR	R	NR	R	NR	R	NR	ALL
**Br**	46	3	2	2	4	2	0	1	5	17	0	0	1	25	15	40
**Bo**	47	3	2	2	4	2	0	1	6	17	0	0	1	25	16	41
**Ca**	21	0	4	1	0	0	0	0	2	2	0	0	0	3	6	9
**Pr**	9	1	2	0	0	2	0	0	0	0	0	0	0	3	2	5
**Ty**	10	0	0	0	0	2	0	0	0	0	0	0	0	2	0	2
**Ak**	44	1	1	1	1	3	0	0	1	13	0	1	2	19	5	24
**Fe**	61	1	7	4	2	4	0	1	34	14	0	0	1	24	44	68
**Ri**	66	0	4	4	1	3	0	3	4	14	0	0	2	24	11	35
**Co**	69	1	3	4	3	4	0	1	5	14	0	2	2	26	13	39
**Si**	71	0	5	4	2	3	0	1	4	15	0	2	1	25	12	37
**Tot.**		**10**	**30**	**22**	**17**	**25**	**0**	**8**	**61**	**106**	**0**	**5**	**10**	**176**	**124**	**300**
																**(47%)**

Singletons^5^	Tot. ORFs		Distribution^2^														
			ANK		TPR		RPE		TNP		TA		PHAGE		Tot.		
	R	NR	R	NR	R	NR	R	NR	R	NR	R	NR	R	NR	R	NR	ALL
**Br**	96	1	0	0	0	0	0	0	5	0	0	0	0	0	5	0	5
**Bo**	112	5	0	0	0	0	0	0	1	1	0	0	0	0	1	1	2
**Ca**	175	1	1	0	1	0	0	0	0	1	0	0	0	0	2	1	3
**Pr**	68	0	1	0	0	0	0	0	0	0	0	0	0	0	1	0	1
**Ty**	56	1	0	0	0	0	0	0	0	0	0	0	0	0	0	0	0
**Ak**	284	3	0	0	0	0	0	0	6	1	1	0	1	0	8	1	9
**Fe**	312	54	4	2	1	0	4	0	2	10	2	1	1	0	14	13	27
**Ri**	153	0	3	0	0	0	1	0	0	0	1	0	1	0	6	0	6
**Co**	97	0	1	0	1	0	1	0	0	0	0	0	0	0	3	0	3
**Si**	114	1	1	0	0	0	1	0	0	0	0	0	0	0	2	0	2
**Tot.**	**1467**	**66**	**11**	**2**	**3**	**0**	**7**	**0**	**14**	**13**	**4**	**1**	**3**	**0**	**42**	**16**	**58**
																	**(4%)**

1 C1OGs (see **Figure 4** for description and **Figure 5** and **Figure 7** for distribution of representative and non-representative C1OGs across rickettsial phylogeny).

2 R = representative OGs, NR = non-representative OGs (see **Figure 4** for description).

3 C2OGs (see **Figure 4** for description and **Figure S2** for distribution of representative and non-representative C2OGs across rickettsial phylogeny).

4 Percentage of 637 C2OGs present within each rickettsial genome. The 128 distributions of these OGs are illustrated in **Figure S2** **.**

5 ORFs found in only one rickettsial genome. Does not include false singletons (see **Figure 4**).

A comparison of the distributions of both representative and non-representative C1OGs and their associated singletons uncovers the high occurrence of singleton genes (53%) per representative C1OGs (**Figure 5**). While many singletons may be the product of gene overprediction (discussed below), some could possibly have important species- or strain-specific functions, such as host manipulation. “False singletons”, which depict non-representative OGs with all members from a single genome (**Figure 4C**), contribute less (17%) towards non-representation when identical genes from *R*. *felis* plasmids pRF and pRFδ are not considered (for speculation on the existence of pRFδ see Gillespie et al. [28]). Thus the biological causes of non-representation, such as HGT and gene duplication, tend to occur more within gene families common across multiple rickettsial genomes rather than in unique genes within individual genomes. This is congruent with our determination of the high occurrence within C2OGs of six gene families typically associated with mobile DNA and/or HGT (above).

### The Nature of Non-Representation

The degree of non-representation recovered by OrthoMCL is not a surprise as *Rickettsia* genomes are notorious for being highly reductive [e.g., 36]–[38], having a high occurrence of split genes and pseudogenes [e.g., 22], [23], [32], [39], [40] and limited conservation in important host-recognition proteins such as rickettsial outer membrane protein A (rOmpA) and other cell surface antigens (Scas) [e.g., 41]–[57]. Coupled with this, some of the more recently sequenced genomes (namely both *R*. *bellii* strains and *R*. *felis*) are riddled with gene rearrangements and elevated levels of repetitive elements and transposases [9], [27], and the staggering degree of repetitive sequences and gene duplications in the recently sequenced genome of *Orientia tsutsugamushi* [58] suggest the old paradigms for genome reduction and synteny in Rickettsiaceae need reevaluation. Furthermore, as we recently predicted [28], new evidence is mounting for the presence of plasmids in several members of AG, TRG and SFG rickettsiae (reviewed in Baldridge et al. [59]), with some proteins having high similarity to counterparts encoded on rickettsial chromosomes [e.g., 28], [60]. All of these factors confound the accurate assignment of gene orthology across genomes, and it is important to view our results as algorithm-dependent, which further required manual scrutiny and adjustment.

**Figure 6:**
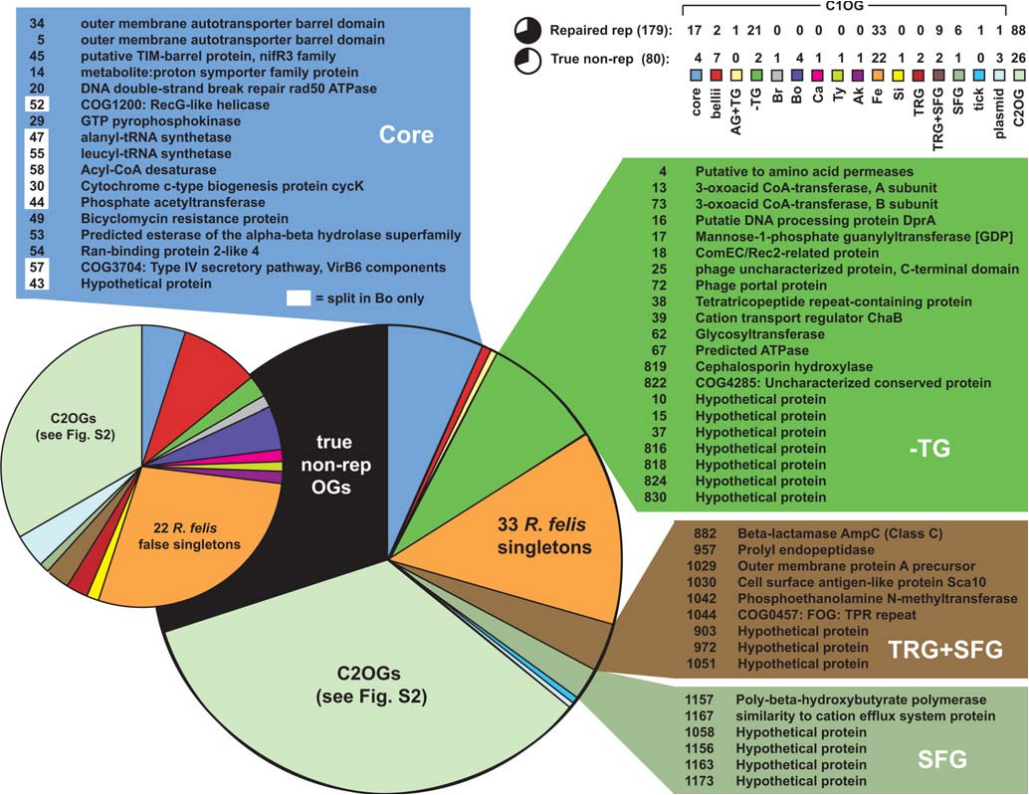
Manual curation of 259 non-representative OGs predicted by OrthoMCL. Schema depicts 179 OGs repaired to representative after stitching together split ORFs (larger pie chart) and remaining true non-representative OGs defined by in-paralogs.

Manual inspection of the 259 non-representative OGs via multiple sequence alignment of each specific case revealed the high occurrence of split genes versus true gene duplications (**Table 4** **;** **Table S1**). Including spurious duplications from the identical *R*. *felis* pRF and pRFδ plasmids, 387 problematic ORFs were eliminated or stitched together to create pseudogene ORFs, resulting in only 80 remaining non-representative OGs defined by true gene duplications. Notably, elimination of identical pRF and pRFδ plasmid genes created 33 additional *R*. *felis* singletons. After “repairing” OGs defined by split ORFs, four distributions contained the majority of C1OGs, illustrating the instances of gene decay from the core, -TG, TRG+SFG, and SFG distributions (**Figure 6**). Regarding the repaired OGs with a core distribution, nearly half of the split genes were from the *R*. *bellii* str. OSU 85-389 genome and include critical genes such as those encoding alanyl- and leucyl-tRNA synthetases and one of the five virB6 components of the type IV secretion system. OGs containing split genes with a -TG distribution include two proteins possibly involved in DNA transformation: a ComEC/Rec2-related protein and a putative DNA processing protein DprA, plus two phage related proteins and a TPR motif-containing protein. This illustrates that genes deleted from the TG genomes involved in conjugation or other methods of foreign DNA uptake are in the process of decaying from the remaining rickettsial genomes. Through the comparison of the proportion of split genes to gene duplications per rickettsial genome (**Table 5**), it is evident that split genes occur more frequently, particularly in SFG rickettsiae, and that both split genes and gene duplications are nearly nonexistent in TG rickettsiae. Interestingly, the genomes with plasmids and elevated levels of transposases and related elements, namely *R*. *felis* and *R*. *bellii*, also have elevated levels of gene duplications.

**Table 4:** Manual evaluation of 259 non-representative OGs across ten rickettsial genomes.

Cause of non-representation^1^	No. OGs	Tot. ORFs	Problem ORFs	Remaining non-rep. after manual curation
split genes only	137	1217	280 split	899 ORFs after concatenation; no non-rep. OGs
gene duplications only	66	425	295 duplicated (207 duplications)	no change (all bona fide non-rep. OGs)
split genes+gene duplications	6	78	9 split; 6 duplicated	66 ORFs after concatenation; all non-rep. OGs
pRFδ only	9	41	9 suspect duplications	32 ORFs; no non-rep. OGs
pRFδ only (*R*. *felis* doublets)	33	66	33 suspect duplications (pRFδ)	33 *R*. *felis* singletons
pRFδ+gene duplications	7	30	8 suspect duplications (pRFδ)	22 remaining ORFs; all non-rep. OGs
pRFδ+split genes+gene duplications	1	5	1 split; 2 suspect duplications (pRFδ)	2 ORFs after concatenation; both non-rep. OGs
**Tot.**	**259**	**1862**	**387 split or spurious ORFs**	**80 non-rep. OGs with 515 ORFs**

1 Split genes may be split multiple times, and multiple gene duplications may occur within single genomes (see **Table S1**).

**Table 5:** Characterization of 259 non-representative OGs per ten rickettsial genomes^1^.

Group	Genome^2^	Split genes^3^			Gene duplications^4^		Total^5^	% Non-representation^6^
**AG**	**Br**	15	30	15	16	56	31	8%
	**Bo**	22	45	23	16	62	38	10%
	**Ca**	20	41	21	2	11	22	5%
**Tot.**		**57**	**116**	**59**	**34**	**129**	**91**	**23%**
**TG**	**Pr**	3	6	3	0	0	3	0.70%
	**Ty**	1	2	1	2	4	3	0.70%
**Tot.**		**4**	**8**	**4**	**2**	**4**	**6**	**1%**
**TRG**	**Ak**	39	87	48	7	34	46	12%
	**Fe**	23	51	28	45	123	68	17%
**Tot.**		**62**	**138**	**76**	**52**	**157**	**114**	**29%**
**SFG**	**Ri**	59	128	69	7	14	66	17%
	**Co**	52	113	61	6	12	58	15%
	**Si**	56	120	64	5	10	61	15%
**Tot.**		**167**	**361**	**194**	**18**	**36**	**185**	**47%**
**Tot. (all)**		**290**	**623**	**333**	**106**	**326**	**396**	

1 Not including 52 instances where pRFδ ORFs cause or further contribute to non-representation.

2 Taxon abbreviations are explained in the **Figure 1** legend.

3 Number of split genes, followed by number of ORFs resulting from splits, followed by overestimated ORFs. Note: split genes may be split more than once.

4 Number of gene duplications, followed by number of duplicated ORFs. Note: some genes are duplicated more than once, and pRF genes are considered duplications of *R*. *felis* chromosomal orthologs.

5 Total number of split ORFs and gene duplication events per genome.

6 Portion of each genome contributing to total non-representation.

### Core and Group-Specific C1OGs

**Figure 7:**
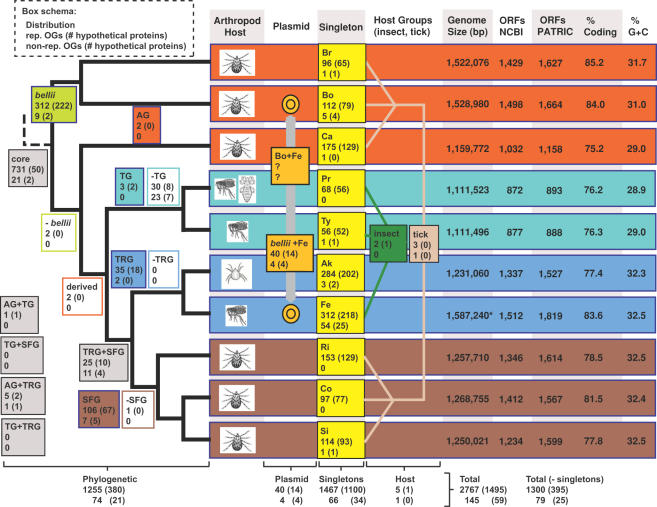
Distribution of representative and non-representative class 1 OGs (C1OGs) and singleton ORFs over estimated rickettsial phylogeny. Boxes depict the distribution of phylogenetic groups, singletons, plasmid associated groups, and host-related groups: Red = AG rickettsiae, aquamarine = TG rickettsiae, blue = TRG rickettsiae, brown = SFG rickettsiae, gray = higher-level groupings, light green = *R*. *bellii* strains only. Orange boxes depict genes found on the pRF plasmid of *R*. *felis* str. URRWXCal2 and chromosomes *R*. *felis* and both *R*. *bellii* strains (as of this publication the *R*. *bellii* plasmids remain unavailable). Genes specific to single rickettsial genomes (singletons) are in yellow boxes, with taxon abbreviations explained in the Figure 1 legend. Host specific groups are defined by green (insect) and tan (tick) boxes. Genome statistics were compiled from the PATRIC and NCBI databases. Cladogram is based on trees shown in Figure 3. Inset in dashed box describes general schema for each box. *Total *R*. *felis* genome size: 1,485,148 bp = chromosome; 62,829 bp = pRF and 39,263 bp = pRFδ.

The distribution of representative (1300) and non-representative (79) C1OGs and singletons are shown over our estimated phylogeny (**Figure 7**). Singletons (1467) are also shown but discussed in a separate section below. Of the 1379 C1OGs, 31% are annotated as hypothetical proteins (HPs), suggesting that a significant amount of even the conserved genes within these rickettsial genomes remain to be characterized. Not considering the *bellii* C1OG, which contains genes unique to the *R*. *bellii* genomes, the amount of HPs within the C1OGs decreases to 18%. The core and lineage specific C1OGs are discussed below.

#### Core rickettsial genes

**Figure 8:**
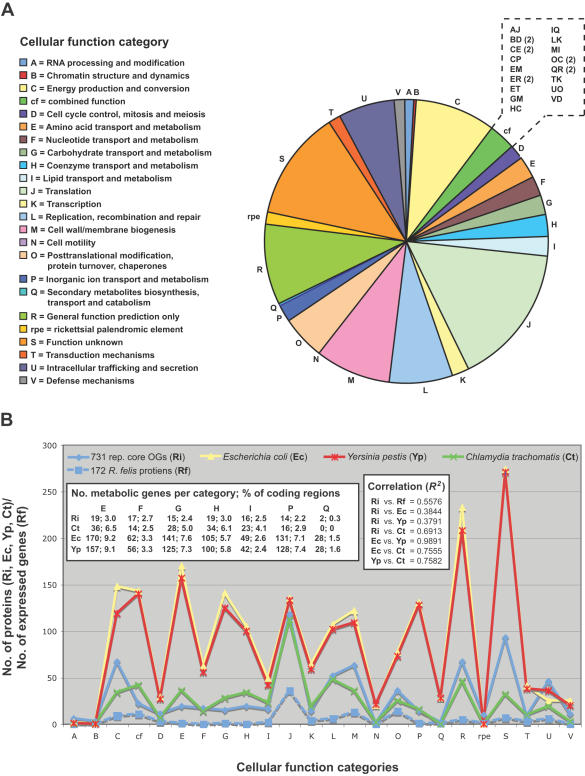
Bioinformatic analysis of core representative OGs. (A) Assignment of 731 core representative RiOGs to predicted cellular function categories. Format follows that established at the COG database (NCBI) except for cf = combined function and rpe = rickettsial palindromic element. (B) Comparison of the distribution of cellular function categories across 731 core rickettsial OGs (Ri), a recent protein expression profile for *R*. *felis* [40] (Rf), and COGs for three other bacteria: *Escherichia coli* (Ec), *Yersinia pestis* (Yp) and *Chlamydia trachomatis* (Ct). Inset at left shows the number of genes per genome for cellular function categories involved in organic and inorganic transport and metabolism (E, F, G, H, I, P, and Q) followed by the percentage these genes comprise of total protein-encoding genes. Results from a six-way regression analysis are shown in the right inset.

OrthoMCL grouped 731 representative and 21 non-representative protein families that are present in all ten analyzed rickettsial genomes (**Table S2**). Thus, the genes encoding these proteins define the foundation of rickettsial biology, such as “house-keeping” functions, as well as rudimentary processes in host cell recognition, invasion and survival (but not necessarily virulence as not all *Rickettsia* spp. are known pathogens). The distribution of the assigned cellular functions of each of these core proteins provides insight on the conservation of cellular activities relative to other bacteria (**Figure 8A**). Not surprising, OGs involved in translation represent the largest functional category (16.14%), as other cellular functions such as amino acid (2.6%), carbohydrate (2.1%), nucleotide (2.3%), and lipid (2.2%) synthesis are less necessary when many of these resources can be obtained from host cells [61], [62]. Analyzing a crude depiction of the *R*. *felis* proteome, Ogawa et al. [40] reached a similar observation as their 172 identified proteins sorted into cellular function categories similar to those assigned for our core proteins, although with far fewer members per category (**Figure 8B**). The core rickettsial protein distribution across cellular function categories is also similar to another obligate intracellular pathogen, *Chlamydia trachomatis*, suggesting that this lifecycle is defined by reduction of many genes with conserved cellular functions (save translation) in facultative intracellular (*Yersinia pestis*) and extracellular (*Escherichia coli*) pathogenic bacteria. The percentage of ORFs coding for metabolic genes is lower in the obligate intracellular bacteria, with exception of the coenzyme transport/metabolism and lipid transport/metabolism genes of *Chlamydia*, which equal and exceed that of the two larger genomes, respectively.

#### AG rickettsiae

Based on phylogeny estimation of over 30 proteins that placed *R*. *canadensis* basal to the TG, TRG and SFG rickettsiae, we categorized it with both *R*. *bellii* strains in the AG rickettsiae [28], a result recovered here and consistent with several previous studies [3; consensus tree of Vitorino et al. [63]]. Conversely, our analysis of OG distribution recovered only two proteins that are unique to AG rickettsiae: RiOG_1416 (Type I restriction-modification system, M subunit) and RiOG_1429 (F pilus assembly protein TraB). RiOG_1416 is truncated in *R*. *bellii* str. OSU 85-389 and extremely truncated in *R*. *canadensis*. Similarly, RiOG_1429 is truncated in *R*. *canadensis*; thus it is unlikely that either ORF is an important signature for AG rickettsiae. Furthermore, while both strains of *R*. *bellii* share 321 unique representative protein families (**Figure 7**, **Table S3**), *R*. *canadensis* only shares two unique proteins with the remaining derived rickettsiae: RiOG_925 (COG0419: ATPase involved in DNA repair) and RiOG_927 (methyltransferase family protein), with the latter likely part of a multigene family with other *R*. *bellii* homologs. Thus, OG distribution provides little evidence for placing *R*. *canadensis* either within AG rickettsiae or as derived. For instance, of the three derived rickettsial groups, *R*. *canadensis* shares more OGs with SFG (13; **Figure S2-C8**) than with either TG (3; **Figure S2-B16**) or TRG (5, **Figure S2-B15**) rickettsiae. However, the three OGs shared between *R*. *canadensis* and TG rickettsiae are all unique sugar transferases, and all three genomes share an unprecedented 52 lost OGs relative to the remaining seven rickettsial genomes (**Table 6** **;** **Figure S2-F3**). Interestingly, *R*. *canadensis* shares zero lost genes with either TRG or SFG rickettsiae. It also shares with *R*. *prowazekii* a unique split gene, *scaI*, that is the most conserved member of the *scas* and is present in all analyzed *Rickettsia* spp. [57]. Thus, while phylogeny estimation places *R*. *canadensis* basal to the TG, TRG and SFG rickettsiae, and common OGs suggest an affinity to SFG and TRG rickettsiae over TG rickettsiae, the mode of gene loss across the lineages branching off after *R*. *bellii* suggests the position of *R*. *canadensis* within our generated phylogeny is well supported, but with possible affinities with TG rickettsiae, which were originally suggested based on serological cross reactivity studies [64]. Accordingly, phylogenetic analysis and signature proteins alone should not be solely used to characterize rickettsial groups, as shared absence of genes may reflect relatedness that is difficult to detect otherwise in these highly reductive genomes.

**Table 6:** OGs missing in the lineage spanning *R*. *canadensis* and TG rickettsiae.

Missing from *R*. *canadensis* and TG rickettsiae (52)^2^		Missing from TG rickettsiae (53)^3^	
RiOG^1^	Annotation	RiOG	Annotation
22	COG1373: Predicted ATPase (AAA+ superfam)	67	Predicted ATPase
973	Acetylglutamate kinase	62	Glycosyltransferase
958	ADP-ribose pyrophosphatase MutT	819	Cephalosporin hydroxylase
955	Clavaminate synthase 1	879	Acylamino-acid-releasing enzyme
966	DNA-damage-inducible protein J	890	AmpG protein
964	Optineurin	886	Blasticidin S-acetyltransferase
982	peptide deformylase	872	COG4912: Predicted DNA alkylation repair enzyme
987	Bacterioferritin comigratory protein	915	DNA repair protein radC homolog
978	Putative integral membrane protein	916	formamidopyrimidine-DNA glycosylase
66	Acetyltransferase	913	gabD
40	Beta-lactamase OXA-18 precursor	888	Magnesium and cobalt transport protein CorA
893	Dihydrofolate reductase type 9	889	methylated-DNA-[protein]-cysteine S-methyltransferase
898	Flavodoxin	875	Periplasmic protein
28	Putative oxidoreductase protein	884	Phosphate regulon transcriptional regulatory protein phoB
877	Putative Zn-dependent hydrolase	908	Predicted metal-dependent hydrolase
64	Putative Zn-dependent hydrolase	906	ribose-phosphate pyrophosphokinase
41	Type I restriction enzyme EcoEI M protein	904	RNA methyltransferase, TrmH family, group 1
891	Na+/H+ antiporter NhaA	4	Putative to amino acid permeases
968	ABC transporter ATP-binding protein	13	3-oxoacid CoA-transferase, A subunit
945	RND efflux system, OM lipoprotein, NodT family	73	3-oxoacid CoA-transferase, B subunit
974	Tellurite resistance protein-related protein	16	Putatie DNA processing protein DprA
960	Multidrug resistance protein mdtA precursor	17	Mannose-1-phosphate guanylyltransferase [GDP]
943	Multidrug resistance protein mdtB	896	Putative amino acid transporter yggA
970	COG0457: FOG: TPR repeat	39	Cation transport regulator ChaB
65	NT domain and HEPN domain	18	ComEC/Rec2-related protein
11	NT domain and HEPN domain	25	phage uncharacterized protein, C-terminal domain
975	addiction module toxin, Txe/YoeB family	72	Phage portal protein
965	prevent-host-death family protein	38	Tetratricopeptide repeat-containing protein
977	prevent-host-death family protein	870	Toxin of toxin-antitoxin system VapC
70	Prophage antirepressor	876	Arp2/3 complex activating protein rickA
949	COG5510: Predicted small secreted protein	901	Ecotin precursor
961	CHP TIGR02217	897	Trichohyalin
950	COG1598: Uncharacterized conserved protein	867	Rickettsial palindromic element (RPE) domain
979	COG3755: Unchar. protein conserved in bacteria	902	Transposase
985	COG5449: Uncharacterized conserved protein	894	CHP TIGR00481
881	COG4804: Uncharacterized conserved protein	822	COG4285: Uncharacterized conserved protein
967	UPF0246 protein FTH_1656		

1 Underscored RiOGs depict non-representative OGs.

2 Including six representative HPs and nine non-representative HPs.

3 Including eight representative HPs and seven non-representative HPs.

**Figure 9:**
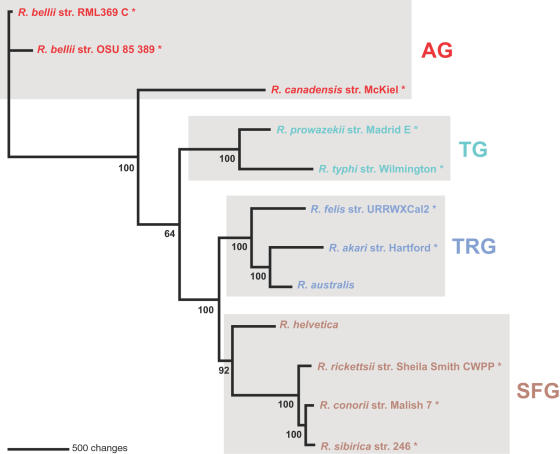
Phylogeny estimation of the ten analyzed rickettsial taxa plus *R. helvetica* and *R. australis* based on 16 proteins. See Table S13 for gene names and sequence accession numbers. Tree estimated under parsimony (see text).

Interestingly, Vitorino et al. [63] recently demonstrated an affinity between *R*. *canadensis* and *R*. *helvetica* based on phylogeny estimation from eight genes, although they concluded that the phylogenetic position of *R*. *canadensis* was unstable, which is consistent with previous studies. For instance, like SFG rickettsiae, *R*. *canadensis* was isolated from ixodid ticks and is maintained transstadially and transovarially [65], [66], grows within the nuclei of its host [65], and contains both *rOmpA* and *rOmpB* genes [67], [68]. However, like TG rickettsiae, *R*. *canadensis* grows abundantly in yolk sac, lyses red blood cells, is susceptible to erythromycin, and forms smaller plaques as compared to SFG rickettsiae [69]. Genomic characteristics are just as anomalous, as despite sharing the same G+C% [26], [69] and only a slightly larger genome size than TG rickettsiae (**Figure 7**), *R*. *canadensis* shares more common repetitive elements with SFG rickettsiae genomes than with any other group [26] and has many similar genes found within the *tra* cluster of *R*. *massiliae* [70]. Switching the position of *R*. *canadensis* in our genome alignment to reflect a derived relationship relative to TG rickettsiae did not improve synteny with the other rickettsial genomes, and despite a large central inversion, *R*. *canadensis* gene order is highly conserved with most of the derived taxa (**Figure S1-D**). In an effort to test a putative affinity between *R*. *canadensis* and *R*. *helvetica* (genome sequence unavailable), we selected 16 existing full or partial gene sequences for *R*. *helvetica* and estimated a phylogeny (**Figure 9**). *R*. *helvetica* is supported as basal to the remaining SFG rickettsiae in an otherwise identical phylogeny estimated from the 731 core rickettsial genes (**Figure 3**), thus refuting an affinity between *R*. *canadensis* and *R*. *helvetica*. The recent phylogenies estimated from 16S rDNA and *groEL* nucleotide sequences, the VirB4 protein and 14 concatenated proteins of the T4SS complex, and entire genome sequences placed *R*. *canadensis* between TG and TRG rickettsiae [26]; however, *R*. *bellii* was not sampled, likely affecting character polarity with the absence of an ancestral taxon. Thus, given our estimation of phylogeny from all available annotated rickettsial genomes, we are confident in the placement of *R*. *canadensis* as basal to the TG, TRG and SFG rickettsiae, although limited similarity is apparent to both *R*. *bellii* genomes as revealed by OG distribution and synteny. It is not unreasonable to predict that *R*. *canadensis* will ultimately group within a fifth distinct rickettsial group once more genomes are sequenced from lesser known rickettsiae, particularly species non-pathogenic to humans.

#### TG rickettsiae

Despite being distinct from the other rickettsial groups with its highly reductive genomes and strictly insect-specific lifestyles, TG rickettsiae were predicted to contain only three unique representative OGs: a putative GTP pyrophosphokinase (RiOG_2080) and two HPs (RiOG_2081 and RiOG_2082). RiOG_2080 is part of a probable multigene family that is duplicated in most rickettsial genomes. These enzymes catalyze the synthesis of guanosine 5′-triphosphate 3′-diphosphate (pppGpp) as well as guanosine 3′,5′-bispyrophosphate (ppGpp) by transferring pyrophosphoryl groups from ATP to GTP or GDP respectively [71], functioning as mediators of the stringent response that coordinate a wide range of cellular activities in reaction to changes in nutritional abundance [72]. While common in multiple variable copies across the sampled genomes, the role lineage specific GTP pyrophosphokinases play in accommodating the different modes of intracellular replication and intercellular spreading by different rickettsial groups is worth exploring. RiOG_2081 is an uncharacterized protein conserved in a limited number of other bacteria (COG3274) and unknown from non-TG rickettsiae. The distribution of this protein, a putative membrane associated acyltransferase, in many pathogenic bacterial species and one bacteriophage, PhiV10, is interesting (**Table 7**). Finally, RiOG_2082 is a small putative ORF that BLASTs to no other organisms, with the start codon missing in *R*. *typhi*.

**Table 7:** Results of a BLASTP search for RiOG_2081 using RP338 (*R*. *prowazekii*) as a query^1^.

Accession no.	Taxon/annotation	score (bits)	E value
**NP_220721**	***Rickettsia prowazekii*** ** str. Madrid E; HP RP338**	**546**	**7.00E-154**
**YP_067290**	***Rickettsia typhi*** ** str. Wilmington; HP RT0328**	**506**	**8.00E-142**
YP_157885	*Azoarcus* sp. EbN1; conserved HP, predicted acyltransferase 3 family	92.8	3.00E-17
YP_039445	*Bacillus thuringiensis* serovar *konkukian* str. 97-27; HP BT9727_5136	81.3	8.00E-14
ZP_00239274	*Bacillus cereus* G9241; membrane protein, putative	79	4.00E-13
NP_847850	*Bacillus anthracis* str. Ames; HP BA5704	78.6	5.00E-13
YP_897634	*Bacillus thuringiensis* str. Al Hakam; possible membrane protein	78.2	6.00E-13
YP_086718	*Bacillus cereus* E33L; probable membrane protein	78.2	7.00E-13
NP_932896	*Vibrio vulnificus* YJ016; HP VV0103	77	2.00E-12
EDK27457	Unclassified Vibrionales; putative inner membrane protein	76.6	2.00E-12
ZP_01261849	*Vibrio alginolyticus* 12G01; putative inner membrane protein	76.3	3.00E-12
NP_799345	*Vibrio parahaemolyticus* RIMD 2210633; putative inner membrane protein	74.7	7.00E-12
NP_760091	*Vibrio vulnificus* CMCP6; HP VV1_1144	74.7	7.00E-12
ZP_01066487	*Vibrio* sp. MED222; putative inner membrane protein	70.5	1.00E-10
ZP_01474781	*Vibrio* sp. Ex25; HP VEx2w_02002647	69.3	3.00E-10
ZP_00833544	*Yersinia intermedia* ATCC 29909; COG3274	68.6	6.00E-10
YP_001008263	*Yersinia enterocolitica* subsp. enterocolitica 8081; HP YE4126	67.4	1.00E-09
YP_206230	*Vibrio fischeri* ES114; integral membrane protein	67.4	1.00E-09
ZP_00992296	*Vibrio splendidus* 12B01; putative inner membrane protein	67.4	1.00E-09
YP_512280	Phage phiV10; putative acetyltransferase	65.9	4.00E-09
ZP_00823633	*Yersinia bercovieri* ATCC 43970; COG3274	64.7	7.00E-09
ZP_00829271	*Yersinia frederiksenii* ATCC 33641; COG3274	64.3	1.00E-08
NP_521411	*Ralstonia solanacearum* GMI1000; HP RSc3292	63.9	1.00E-08
YP_100876	*Bacteroides fragilis* YCH46; HP BF3599	62	6.00E-08
YP_213008	*Bacteroides fragilis* NCTC 9343; HP BF3402	61.6	6.00E-08
ZP_01237231	*Vibrio angustum* S14; HP VAS14_21937	61.2	9.00E-08
ZP_00826782	*Yersinia mollaretii* ATCC 43969; COG3274	60.8	1.00E-07
ZP_01160312	*Photobacterium* sp. SKA34; HP SKA34_16770	60.5	1.00E-07

1 Only sequences with a score greater than 60 bits are shown; of 88 subjects, no other rickettsiae were retrieved.

While a wealth of unique genes seemingly does not define TG rickettsiae, 53 unique gene loss events may offer insight into the streamlined manner of their evolution (**Table 6**). The loss of the Arp2/3 complex activating protein, *rickA*, from TG rickettsiae has been well-documented, and distinguishes this group in its mode of host cell spreading [73], [74]. Interestingly, our comparative analysis has revealed two other curious proteins that are present and conserved in all other non-TG rickettsiae genomes. The first is RiOG_897, a putative trichohyalin, which are intermediate filament-associated proteins found predominantly in the hair follicle cells of mammals [75], [76] but also expressed in the hard palate, tongue, nail bed, and a suite of pathological epidermal tissues [77], [78]. We discuss more about trichohyalins below in regards to insect-associated rickettsiae containing a unique trichohyalin-like homolog that is different from the gene found in all other non-TG rickettsiae. The second interesting OG (RiOG_901) found exclusively in non-TG rickettsiae is an ecotin-like protein. Ecotin is a dimeric periplasmic protein described in *Escherichia coli* that belongs to the protease inhibitor I11 (ecotin) family (PF03974). Ecotin inhibits several pancreatic serine proteases, including chymotrypsin, trypsin, elastases, factor X, kallikrein, as well as a variety of other proteases [79]–[81]. Eggers et al. [82] have shown that ecotin protects *E*. *coli* from neutrophil elastase (NE), a mammalian serine protease demonstrated to be important for neutrophil killing of several gram-negative bacteria. Specifically, NE cleaves ompA causing increased permeability to the bacterial outer membrane [83]. Once NE translocates across the vulnerable outer membrane, it functions in inhibiting bacterial cell growth and repair, causing cell death. The presence of ecotin in the periplasm inhibits NE function, thus fostering recovery and growth of the invading bacterial cells [82]. Given the diversity of rickettsial outer membrane surface proteins, particularly the Scas [55], it is reasonable to suggest that one or several surface proteins present in all non-TG rickettsiae may be dependent upon the putative NE inhibitory function of RiOG_901.

#### TRG rickettsiae

Based on the monophyly of its sampled members (*R*. *felis* and *R*. *akari*), its strongly supported position in our estimated rickettsial phylogeny, an affinity with AG rickettsiae plasmid-associated genes, and the use of both acarines and insects as primary invertebrate hosts, we erected the TRG rickettsiae as a third derived lineage of *Rickettsia* [28]. OrthoMCL predicted 37 OGs unique to TRG rickettsiae (**Table 8**). Of the three other rickettsial lineages, TRG shares more common OGs with SFG rickettsiae (36) than with TG rickettsiae (0) or AG rickettsiae (6) (**Figure 7**), reflecting its shared common ancestry with the “true” spotted fever group taxa. However, exclusion of *R*. *canadensis* sheds light on our previously described affinities of TRG rickettsiae with AG rickettsiae (**Table 9**). For instance, 26 OGs are shared between the *R*. *bellii* genomes and TRG rickettsiae (**Figure S2-C25**), with six of these annotated as members of toxin-antitoxin (TA) modules, and another two annotated as bacteriophage-derived proteins. Additionally, the *R*. *felis* genome shares 44 OGs with the *R*. *bellii* genomes (**Figure 7**), six of which are annotated as members of TA modules, with another one annotated as bacteriophage-derived protein. Furthermore, the *R*. *akari* genome shares 10 OGs with the *R*. *bellii* genomes (**Figure S2-B23**), and two of these OGs are predicted members of TA modules. This high presence of TA system components, as well as bacteriophage-derived proteins, attests to our previous observations that AG (at least *R*. *bellii*) and TRG rickettsiae are linked via conjugative systems and have a pronounced presence of similar plasmid (and now phage) related ORFs, likely the end products of various lateral gene exchanges between these distantly related groups.

**Table 8:** OGs present only in TRG rickettsiae.

RiOG^1^	Annotation (37)^2^
2043	COG1670: Acetyltransferases, incl. N-acetylases of ribosomal proteins
2078	Predicted acetyltransferase
2062	Predicted hydrolase or acyltransferase
2038	Putative cysteine protease yopT-like
2047	5-Formyltetrahydrofolate cyclo-ligase
1125	alanine racemase
2033	Outer membrane protein A precursor
2037	Outer membrane protein A precursor
2046	Outer membrane protein A precursor
2076	Outer membrane protein A precursor
2049	ABC transporter, ATP-binding protein
2075	Cell surface antigen-like protein Sca7
2059	Ankyrin repeat
2066	COG1487: Predicted nucleic acid-binding protein, contains PIN domain
2056	Probable antitoxin of toxin-antitoxin stability system
2069	addiction module toxin, Txe/YoeB family
2050	Virulence-associated protein B
1483	CHP
2068	CHP

1 Underscored RiOGs depict non-representative OGs.

2 Including 18 representative HPs.

**Table 9:** OGs present only in *R*. *bellii* strains and TRG rickettsiae.

Present in *R*. *bellii* strains and TRG rickettsiae (26)^2^	
RiOG^1^	Annotation
1245	HicB family
1261	Phage-related transcriptional regulator
1215	phage host specificity protein
1128	Transcriptional regulator
1266	PIN domain containing protein
1256	Antitoxin of toxin-antitoxin system StbD
1262	Cytotoxic translational repressor of toxin-antitoxin (TA) system RelE
1251	Cytotoxic translational repressor of toxin-antitoxin system RelE
1243	Growth inhibitor
1240	putative addiction module antidote protein, CC2985 family
1269	Transposase
1	Probable transposase for insertion sequence element
1260	CHP

Present in *R*. *bellii* strains and *R*. *felis* (44)^3^	
RiOG	Annotation
1437	Cell surface antigen-like protein Sca9
1418	Alkylated DNA repair protein
1423	cAMP-inducible prespore protein D7 precursor
1415	Caspase recruitment domain-containing protein 15
1451	Citrate-proton symporter
1410	Fic family protein
1438	Heavy metal tolerance protein precursor
1420	invasion protein homolog
1442	Lysine efflux permease
1428	Lysozyme
1417	Menaquinone biosynthesis methyltransferase ubiE
1406	nucleotidyltransferase substrate binding protein, HI0074 family
1452	Putative AAA+ superfamily ATPase
1431	Putative integrase/recombinase HI1572
1436	Streptomycin 6-kinase
1414	Ankyrin repeat
1434	phage major capsid protein, HK97 family
1426	Bacterial transcription activator, effector binding domain
1446	Antitoxin of toxin-antitoxin system Phd
1408	prevent-host-death family protein
1427	prevent-host-death family protein
1430	probable addiction module killer protein
1435	Toxin of toxin-antitoxin (TA) system
1450	Transcriptional regulator, AbrB family
1411	CHP
1413	CHP

Present in *R*. *bellii* strains and *R*. *akari* (10)^4^	
RiOG	Annotation
1458	endo/excinuclease amino terminal domain protein
1472	Leucine-rich repeat-containing protein 45
1489	Reticulocyte-binding protein 1 precursor
1465	Ankyrin repeat
1466	COG1848: Predicted nucleic acid-binding protein, contains PIN domain
1470	Prevent-host-death protein
1480	CHP

1 Underscored RiOGs depict non-representative OGs.

2 Including 12 representative HPs and 1 non-representative HP.

3 Including 14 representative HPs and 4 non-representative HPs.

4 Including 3 HPs.

Despite the abovementioned characteristics shared between AG and TRG rickettsiae, the TRG rickettsiae also share three TA components exclusively with SFG rickettsiae (**Table S4**). Additionally, SFG rickettsiae and the *R*. *bellii* genomes have three TA components not found in the other analyzed genomes (**Figure S2-D7**). This alludes to the likelihood that SFG rickettsiae and *R*. *bellii* have also had lateral exchange between plasmids at some point in their evolution, although not nearly to the degree that TRG and the *R*. *bellii* genomes have had. For instance, of the 27 OGs shared between *R*. *felis* and SFG rickettsiae (**Figure S2-C1**), only three are components of TA modules (**Table S4**). And of the 22 OGs shared between *R*. *akari* and SFG rickettsiae (**Figure S2-C2**), none are predicted as components of TA modules. This distinction of the close relatedness of TRG to AG rickettsiae (at least the *R*. *bellii* genomes) relative to its sister clade, SFG rickettsiae, based on plasmid associated gene distribution is critical in understanding the mode of gene loss from the last common ancestor of *Rickettsia*, as well as the degree conjugative systems have contributed to the architecture of these genomes.

Based on phylogeny estimation of 16S rDNA sequences, the largest clade recovered to date for TRG rickettsiae included *R*. *akari*, *R*. *felis*, *R*. *australis*, and poorly characterized rickettsiae from booklouse (*Liposcelis* sp.) and parasitic wasp (*Neochrysocharis* sp.) hosts [16]. In addition, Reeves et al. [84] recently identified two novel rickettsial genotypes from the mite *Ornithonyssus bacoti* from Egypt that are closer to TRG rickettsiae than the other rickettsial groups based on partial sequence comparison of the 17 kD antigenic gene. Aside from *R*. *australis*, which has been found exclusively in tick hosts, none of these taxa purportedly parasitize ticks, with *R*. *akari* found in mites [85], *R*. *felis* found in fleas [51], [86]–[89], and the other unnamed *Rickettsia* spp. known only from their booklouse, wasp and mite hosts. Thus the group is interesting from an arthropod host perspective as well as from its apparent affinities to the *R*. *bellii* genomes. In light of this, we suggested that *R*. *australis* would continue to group within the TRG rickettsiae [28], as it has previously done in some cases wherein one or few genes were analyzed [e.g., 16], [63], [90]–[92]. Our dataset including 16 gene sequences from *R*. *helvetica* (discussed above) also contained eight sequences from *R*. *australis* and grouped this taxon with *R*. *akari* in a clade subtended by *R*. *felis* with strong bootstrap support (**Figure 9**). However, while the TRG rickettsiae is still recovered when *R*. *akari* and *R*. *australis* are analyzed in the absence of *R*. *felis* [49], [92], [93], the exclusion of *R*. *akari* in the presence of *R*. *australis* and *R*. *felis* [51] failed to recover a monophyletic TRG rickettsiae. Furthermore, while four of the eight single gene phylogeny estimates by Vitorino et al. [63] recovered the TRG rickettsiae, the consensus tree did not, as the TG rickettsiae was placed within the TRG rickettsiae, splitting the *R*. *akari*/*R*. *australis* clade from *R*. *felis*. Thus, the TRG rickettsiae is not easily demonstrated as a distinct lineage of rickettsiae unless the taxon and character sampling is robust enough for this intriguing lineage to emerge (**Figure 9**; [28]).

#### SFG rickettsiae

The majority of the described species of *Rickettsia* fall within the SFG rickettsiae. The analyzed spotted fever group genomes form a monophyletic cluster of taxa with little sequence divergence relative to the other rickettsial groups (**Figure 3**). OrthoMCL predicted 113 OGs that are unique to SFG rickettsiae (**Table 10**). Of note, in addition to the four core rickettsial proline/betaine transporters (**Table S2**), SFG rickettsiae contain two variant copies (RiOG_1314 and RiOG_1332). Other transporters unique to SFG rickettsiae include three ATPase and permease components of an ABC-type multidrug transporter (RiOG_1347, RiOG_1364 and RiOG_1365), an ATP-binding protein similar to ABC transporter (RiOG_1376), an MSF-like sugar transporter (RiOG_1355), and an RND family efflux transporter (RiOG_1294). While high numbers of transporters are expected in *Rickettsia* to counterbalance depleted metabolic pathways and acquire host resources, it is unclear why the SFG rickettsiae have elevated levels of unique components of organic and inorganic transport systems relative to the other three rickettsial groups. As with TG rickettsiae, there are group-specific GTP pyrophosphokinases (RiOG_1350 and RiOG_1361) in SFG rickettsial genomes, and their role in a group-specific stringent response is worthy of attention. Like AG and TRG rickettsial genomes, SFG rickettsiae have group-specific ANK repeat containing proteins, with a particular one (RiOG_1344) similar to metazoan tankyrases, telomeric repeat binding factor-interacting ANK-related ADP-ribose polymerases. Aside from potentially playing key roles in the maintenance of telomere function [e.g., 94], tankyrases have been implicated in mitogen-activated protein kinase signaling [95], regulation of cell death [96], [97] and viral inhibition [98].

**Table 10:** OGs present only in SFG rickettsiae.

RiOG^1^	Annotation (113)^2^
1312	COG0522: Ribosomal protein S4 and related proteins
1378	Acetate kinase
1342	Acetyltransferase
1313	COG1835: Predicted acyltransferases
1317	COG0840: Methyl-accepting chemotaxis protein
1350	GTP pyrophosphokinase
1361	GTP pyrophosphokinase
1284	Predicted NTPase
1334	Prolyl endopeptidase precursor
1330	Putative DNA processing protein DprA
1398	similarity to D-alanyl-D-alanine dipeptidase
1344	Tankyrase-1
1286	Type I restriction enzyme EcoBI specificity protein
1363	P pilus assembly protein FimD
1386	P pilus assembly protein FimD
1157	Poly-beta-hydroxybutyrate polymerase
1360	Cell surface antigen Sca3
1349	Cell surface antigen-like protein Sca8
1383	Cell surface antigen-like protein Sca8
1347	ABC-type multidrug transport syst., ATPase and permease components
1364	ABC-type multidrug transport syst., ATPase and permease components
1365	ABC-type multidrug transport syst., ATPase and permease components
1376	similarity to ABC transporter ATP-binding protein
1314	Proline/betaine transporter
1332	Proline/betaine transporter
1294	RND family efflux transporter
1355	MFS type sugar transporter
1167	similarity to cation efflux system protein
1307	Multidrug resistance protein mdtB
1345	Rickettsial palindromic element (RPE) domain
1357	Rickettsial palindromic element (RPE) domain
1380	Ankyrin repeat
1388	Ankyrin repeat
1315	Ankyrin repeat domain-containing protein 28
1392	putative transposable insertion element
1382	COG4804: Uncharacterized conserved protein
1299	CHP
1324	CHP
1341	CHP
1348	CHP
1354	CHP

1 Underscored RiOGs depict non-representative OGs.

2 Including 67 representative HPs and five non-representative OGs.

Using EasyGene [99], a program that ranks prokaryotic predicted ORFs based on statistical significance, Nielsen and Krogh [100] determined that the *R*. *conorii* str. Malish 7 genome was over-annotated by 16%, ranking 7th among most over-annotated replicons in a sample of 143 prokaryotic genomes. Specifically, EasyGene determined 225 RefSeq genes to be false, with 34 additional genes predicted by EasyGene that were not called in the original study [22], [23]. Aside from possible gross ORF over-prediction in all ten rickettsial genomes (discussed below), our analysis yielded many OGs with imperfect representation within the SFG group, as 54 OGs are found exclusively in the *R*. *conorii* and *R*. *sibirica* genomes (**Figure S2-A1**), 52 are found exclusively in the *R*. *rickettsii* and *R*. *sibirica* genomes (**Figure S2-A2**), and 36 are found exclusively in the *R*. *rickettsii* and *R*. *conorii* genomes (**Figure S2-A3**). Given that the SFG rickettsial genomes have elevated split genes as compared to other rickettsial genomes (**Table 5**; **Table S1**), our findings and those of Nielsen and Krogh [100] hint at a pronounced rate of pseudogenization in SFG rickettsiae depicted by a patchy distribution of split and truncated ORFs decaying from the ancestral SFG genome.

One hallmark occurrence of probable pseudogenization in SFG rickettsiae involves a Sec7-domain-containing protein known in prokaryotes only from *Rickettsia* and *Legionella* species [101]. The *Legionella* counterpart of this curious protein, named RalF, is a guanine nucleotide exchange factor that recruits ADP-ribosylation factor to occupied phagosomes, permitting *Legionella* to replicate free from the host immune system [102]. The rickettsial RalF xenolog (RiOG_19), including the N-terminal Sec7 domain and immediate flanking Sec7-capping-domain [103], is present in all rickettsial genomes except for SFG rickettsiae and *R*. *canadensis*, suggesting a biological mechanism that has been lost from the true spotted fever group and *R*. *canadensis*. Unlike *Legionella* RalF, which has a short (44 aa) C-terminal tail containing a type 4 secretion system (T4SS) signal sequence [104], the rickettsial genes encode an additional variable domain (97–315 aa) between the Sec7-capping-domain and the C-terminal tail. Within this third domain lies a region immediately flanking the predicted T4SS signal sequence that is extraordinarily rich in proline residues, much like the P-rich domain of *rickA* proteins [74]. Interestingly, the SFG genomes each contain small ORFs corresponding to the tails of the RalF-like sequences. A similar sequence within the *R*. *canadensis* genome (not annotated) also spans this region yet is riddled with frame-shift mutations. Given that *Rickettsia*, unlike *Legionella,* quickly lyse the phagosome upon host cell entry, the function of a RalF xenolog, particularly given its curious distribution in the rickettsial tree, is worthy of investigation. Finally, full intact RalF xenologs in both TRG rickettsial genomes further attest the distinction of this lineage from the SFG rickettsiae [28].

### Arthropod Host-Specific OGs

Several studies have demonstrated the presence of certain rickettsial species outside of their natural arthropod hosts. For example, the louse (and less often flea) associated *R*. *prowazekii* has been found in ticks in Africa [105] and Mexico [106], and was also reported in acarids from flying squirrels in the United States [107]. However, it should be recognized that many blood-feeding arthropods have a wide range of vertebrate hosts and likely act as reservoirs for a variety of bacteria that incidentally fall outside of their natural arthropod vector. To this extent reports of pathogenic bacteria (i.e., *R*. *prowazekii*) in unusual vectors need to be substantiated beyond simple detection in these foreign hosts, and caution should be taken when immediately assigning novel host associations. Given the low frequency of resident bacteria in many natural arthropod populations [108], substantiation of novel arthropod hosts can be achieved in the field by robustly sampling other invertebrate and vertebrate animals from the same locality that may actually be the true host of the incidentally collected bacterium. Furthermore, laboratory studies would be needed to determine the pathogenicity, if any, that the bacterium causes in its novel host. However, laboratory inoculation of an animal may result in pathogenesis only because the number of bacteria far exceeded what occurs in nature, thus compromising an immune system that under natural circumstances is quite capable of killing the pathogen. Furthermore, demonstrating laboratory bacterial infection or vectorization in a foreign host, for example *R*. *conorii* in the body louse [109], may initially prove successful, but eventually will clear from the host as it would from natural populations. For instance, *Rickettsia* have been grown in mosquito cell lines, yet to our knowledge no wild caught mosquitoes to date have been shown to act as hosts to any *Rickettsia*. In fact, based on the analysis of the highly divergent *sca* genes in rickettsiae, which are suspected to directly interact with host cell proteins [47], [110], Blanc et al. [55] concluded that rapid evolution of such important host colonization genes likely keep *Rickettsia* host ranges quite narrow.

Given our conservative stance on definitive rickettsial arthropod hosts, we have chosen to present the predicted genes that are exclusive to insect associated *Rickettsia* and tick associated *Rickettsia* (as depicted in **Figure 7**). Because only one analyzed genome is from a mite-associated species (*R*. *akari*), we have no comparative analysis to describe potential mite-specific rickettsial genes. However, the list of singleton genes found in *R*. *akari* may provide a start to such an approach (see below).

#### Insect-associated rickettsiae

Three of the ten analyzed rickettsial genomes have definitive insect hosts, with *R*. *typhi* and *R*. *felis* reported from rodent, shrew and feline [51], [86]–[88] associated fleas, and *R*. *prowazekii* predominantly pathogenic in lice, as well as fleas in the sylvatic form. Thus these three rickettsial lineages share common arthropod hosts at least in fleas. Regarding *R*. *typhi*, It has become apparent that the ecology of murine typhus in both south Texas and southern California, where the classic cycle of *R. typhi* involving commensal rats and primarily the rat flea (*Xensopsylla cheopis*), has been replaced by the Virginia opossum (*Didelphis virginiana*)/cat flea (*Ctenocephalides felis*) cycle. For instance, Sorvillo et al. [111] demonstrated the association of 33 cases of locally acquired murine typhus in Los Angeles County with seropositive domestic cats and opossums. However, urban rat/flea populations are still the main reservoir of *R. typhi* worldwide and particularly in many cities where urban settings provide a constellation of factors for the perpetuation of murine typhus, including declining infrastructures, increased immunocompromised populations, homelessness, and high population density of rats and fleas. Thus, aside from the reported louse host of *R*. *prowazekii* and a laboratory demonstration that *R. typhi* infection is lethal for human body lice [112] despite *R*. *typhi* being unknown from wild lice, these three rickettsial taxa are all capable of infecting and causing pathogenicity in an overlapping range of flea species, prompting a genomic comparison to detect common genes possibly involved in flea cell invasion and pathogenicity.

Despite the vast evolutionary divergence between arachnids and hexapods, two lineages with a common ancestor estimated to have split over 500 million years ago [113], only two OGs (RiOG_1496 and RiOG_1497) specific to the *R*. *prowazekii*, *R*. *typhi* and *R*. *felis* genomes were predicted by OrthoMCL (**Figure 5** **,** **Figure 7** **,** **Table 11**). However, these genes are exceptionally interesting from two perspectives. First, while the ORFs encoding both OGs are contiguous in all three genomes, they are present only on the pRF plasmid and not the chromosome of *R*. *felis*, suggesting a possible lateral exchange of these genes between TG rickettsiae and the *R*. *felis* genome. Second, these ORFs share little homology with genes from other organisms, and the taxonomic distribution of these organisms is quite intriguing. RiOG_1496 is annotated as myosin-11 and has close similarities to RiOG_1454, which is annotated as a HP found in the *R*. *felis* genome as well as both *R*. *bellii* genomes. Furthermore, RiOG_897 (discussed above), a predicted trichohyalin-like protein found in all analyzed rickettsial genomes but TG rickettsiae, has limited similarity with RiOG_1496. Aside from the more general functions described above, trichohyalin also acts as a cross-bridging protein that assists in the coordination of mechanical strength between the peripheral cell envelope barrier structures and cytoplasmic keratin filament networks [114]. The lysosomal cysteine protease, cathepsin L, which is critical for skin and hair follicle homeostasis, likely uses trichohyalin as a substrate [115]. Recently, Ou et al. [116] determined that a trichohyalin homolog, DYF-14, in the nematode *Caenorhabditis elegans* is essential for cilium biogenesis. Thus, this group of proteins seems to be critical for epithelial cell maintenance in a wide range of animals, and the presence of similar proteins in TG rickettsiae may hint at a molecular function involved with epithelial (invertebrate host) or endothelial (vertebrate host) cell entry and modification, as both *R*. *typhi*, *R*. *prowazekii* and *R*. *felis* enter their vertebrate hosts transdermally through inoculation or inhalation of insect feces.

**Table 11:** Results of BLASTP searches evaluating two OGs (1496 and 1497) predicted by OrthoMCL to contain only insect-associated rickettsiae.

RiOG_1496					
Accession No.	Annotation	Taxon	score (bits)	E value	OG
**NP_220662**	**HP RP278**	***Rickettsia prowazekii*** ** str. Madrid E**	**484**	**5.00E-135**	**1496** A
**YP_067231**	**CHP**	***Rickettsia typhi*** ** str. Wilmington**	**431**	**3.00E-119**	**1496** A
**YP_247443**	**HP RF_p27**	***Rickettsia felis*** ** URRWXCal2**	**102**	**3.00E-20**	**1496** A
YP_246459	HP RF_0443	*Rickettsia felis* URRWXCal2	67	2.00E-09	1454^B^
ZP_01379825	HP RbelO_01000612	*Rickettsia bellii* OSU 85-389	50.1	2.00E-04	1454^B^
YP_537715	HP RBE_0545	*Rickettsia bellii* RML369-C	48.9	5.00E-04	1454^B^
NP_975020	Ribose/Galactose ABC transporter, permease component	*Mycoplasma mycoides* subsp. mycoides SC str. PG1	43.9	0.014	------
ZP_01380625	HP RbelO_01001434	*Rickettsia bellii* OSU 85-389	37.4	1.3	897^C^
YP_537282	HP RBE_0112	*Rickettsia bellii* RML369-C	37.4	1.3	897^C^
XP_973544	PREDICTED: similar to SMC6 protein	*Tribolium castaneum*	35.4	5	------
Q805A1	SMC protein 5	*Xenopus laevis*	35	6.6	------
XP_956017	HP	*Neurospora crassa* OR74A	35	7.7	------
XP_783551	PREDICTED: HP	*Strongylocentrotus purpuratus*	34.7	7.9	------
XP_001254413	PREDICTED: similar to citron, partial	*Bos taurus*	34.7	9.5	------

RiOG_1497					
Accession No.	Annotation	Taxon	score (bits)	E value	OG
**NP_220661**	**HP RP277**	***Rickettsia prowazekii*** ** str. Madrid E**	**431**	**2.00E-119**	**1497** B
**YP_067230**	**rickettsial CHP**	***Rickettsia typhi*** ** str. Wilmington**	**366**	**4.00E-100**	**1497** B
**YP_247444**	**HP RF_p28**	***Rickettsia felis*** ** URRWXCal2**	**187**	**5.00E-46**	**1497** B
YP_720393	serine/threonine protein kinase	*Trichodesmium erythraeum* IMS101	35.8	1.7	------
CAF95313	unnamed protein product	*Tetraodon nigroviridis*	35	2.7	------
YP_537955	HP RBE_0785	*Rickettsia bellii* RML369-C	34.7	3.5	1439^D^
ZP_01380188	HP RbelO_01000982	*Rickettsia bellii* OSU 85-389	34.7	3.5	1439^D^
ZP_01546758	HP SIAM614_07403	*Stappia aggregata* IAM 12614	34.3	5	------
XP_361067	HP MG03610.4	*Magnaporthe grisea* 70-15	33.9	6.5	------

A Myosin-11.

B Consensus annotation = HP.

C Trichohyalin. OG_897 also contains VBI2812RCa_1005 (ZP_01347956.1), VBI0166RF1_1469 (YP_247242.1), VBI0269RA_1318 (ZP_00340773.1), VBI0113RR_1403 (ZP_00154140.1), VBI2627RCo_1353 (NP_360825.1), and VBI0076RS_1050 (ZP_00142696.1) (all but TG rickettsiae).

D Consensus annotation = HP. OG_1439 also contains VBI0166RF1_0910 (YP_246763.1).

Aside from sharing limited homology to these other OGs, RiOG_1496 is also similar to a predicted permease component of a ribose/galactose ABC transporter from the bacterium *Mycoplasma mycoides* (mollicutes: Spiroplasma group), the etiological agent of contagious bovine pleuropneumonia. Interestingly, a similar ORF is present in the cow genome, possible hinting at a horizontal exchange between *M*. *mycoides* and its bovine host. RiOG_1496 also Blasts to sequences from three other metazoans, the rust red flour beetle, *Tribolium castaneum*, the African clawed frog, *Xenopus laevis*, and the California purple sea urchin, *Strongylocentrotus purpuratus*. The beetle and frog ORFs are predicted as structural maintenance of chromosomes (SMC) proteins 6 and 5, respectively. SMC proteins are involved in such cellular processes as chromosome condensation, sister chromatid cohesion, chromosome partitioning, dosage compensation, DNA repair, and recombination [e.g.], [ 117]–[119]. In *Bacillus subtilis*, an SMC protein (BsSMC) plays a role in chromosome organization and partitioning, and has been shown to affect supercoiling *in vivo*, most likely by constraining positive supercoils, an activity contributing to the compaction and organization of chromosomes [120]. The ORF from the sea urchin, as well as one final BLASTP hit to a sequence from *Neurospora crassa*, a type of red bread mold of the phylum Ascomycota, are annotated as HPs.

Like RiOG_1496, RiOG_1497 had only a few BLASTP hits with significant alignments, yet they cover a range of diverse organisms. RiOG_1497 shares limited similarity with RiOG_1439, which is annotated as a HP and found only in the *R*. *bellii* genomes and the chromosomal genome of *R*. *felis*. Regarding eukaryotes, RiOG_1497 shares limited similarity with HPs from the green spotted pufferfish, *Tetraodon nigroviridis*, and the rice blast fungus, *Magnaporthe grisea*. RiOG_1497 also Blasts to a HP from another α-proteobacterium, *Stappia aggregata* (Rhodobacterales). Interestingly, there is also limited similarity between RiOG_1497 and a serine/threonine protein kinase from the marine filamentous cyanobacterium, *Trichodesmium erythraeum*.

OrthoMCL predicted zero non-representative OGs for the insect-associated *Rickettsia* (**Figure 5** **,** **Figure 7**), and only two representative and two non-representative OGs are present in all other genomes except the insect-associated rickettsiae (depicting shared lost genes in the insect-associated genomes) (**Figure S2-F6**). Both representative OGs (RiOG_948 and RiOG_951) are HPs, while the two non-representative OGs, RiOG_814 and RiOG_817, are annotated as a conserved uncharacterized bacterial protein (COG4374) and a HP, respectively. Thus, only the poorly characterized tandem gene group of RiOG_1496 and RiOG_1497 exists for attempting to distinguish the insect-associated *Rickettsia* from the other lineages with non-insect hosts.

Although the similarity of both RiOG_1496 and RiOG_1497 to the sequences described above is limited, it is nonetheless interesting that their distribution as contiguous ORFs in the TG rickettsiae and the *R*. *felis* pRF plasmid is unique amongst the analyzed rickettsial genomes. It is also interesting that at least one of the ORFs (RiOG_1496) has homology to vertebrate smooth muscle protein myosin-11, which is known to be expressed in the esophagus and trachea of humans, as well as trichohyalin, a protein associated with various healthy and pathological epithelial cell types. Both of these proteins are present at the infection interface between insect associated *Rickettsia* and vertebrate hosts and, at the very least, provide our best guess for a means to distinguish, at the genomic level, insect-associated vertebrate cell invasion from that of acarine. This result of a few examples from the comparative analysis of ten genomes is surprising, and perhaps can be improved upon by the sequencing of more insect-associated rickettsial genomes.

While much of the genome sequencing of rickettsiae has focused on medically important species, it is imperative to consider the species non-pathogenic to humans for comparative biological reasons, in particular for determining the mode of insect-cell invasion and pathogenicity. Studies demonstrating pathogenicity exclusively in insect hosts are limited to 1. male killing in two ladybird beetles (Coleoptera: Coccinelidae), *Adalia bipunctata* [10] and *A*. *decempuncata* [121], and the buprestid beetle *Brachys tessellatus* (Coleoptera: Buprestidae) [122], 2. thelytoky (female parthenogenesis) induction in the serpentine leafminer endoparasitoid, *Neochrysocharis formosa* (Hymenoptera: Eulophidae), [123], 3. reduced weight and fecundity in the pea aphid, *Acrythosiphon pisum* (Hemiptera: Aphididae), [11], [124], and 4. oogenesis induction in the booklouse *Liposcelis bostrychophila* (Psocoptera: Liposcelididae) [125] and in the date stone beetle, *Coccotrypes dactyliperda* (Coleoptera: Scolytidae), [126]. Other organisms beneficial to humans that are affected by insect-associated *Rickettsia* will also be of interest in evaluating insect cell invasion and pathogenicity. For instance, the leafhopper *Empoasca papayae* (Hemiptera: Cicadellidae) is seemingly unaffected by a resident species of *Rickettsia* (PBT) that devastates commercial papaya production (papaya bunchy top disease) [7]. However, the effects on insects by some poorly characterized resident *Rickettsia* species are currently unknown, including those from the springtail *Onychiurus sinensis* (Collembola: Onychiuridae), the bluetongue virus vector *Culicoides sonorensis* (Diptera: Ceratopogonidae), the sweet potato whitefly *Bemisia tabaci* (Hemiptera: Aleyrodidae), the bruchine beetle *Kytorhinus sharpianus* (Coleoptera: Chrysomelidae), and the crane fly *Limonia chorea* (Diptera: Limoniidae) [7], [13], [126]–[128]. Nevertheless, all of these less-understood insect-associated *Rickettsia* spp. are good candidates for comparative genomic analysis with *R*. *prowazekii*, *R*. *typhi* and *R*. *felis* for improving the current knowledge of the mechanisms underlying insect cell invasion and pathogenicity.

#### Tick-associated rickettsiae

Six of the ten analyzed rickettsial genomes have definitive tick hosts, including both *R*. *bellii* genomes, *R*. *canadensis*, *R*. *rickettsii*, *R*. *conorii*, and *R*. *sibirica*. In general, little is known about the definitive host ranges of members of the AG and SFG rickettsiae, partly because few host-specific characteristics have been described for any rickettsial/acarine relationship, but also because multiple arthropod or vertebrate (or other eukaryote) hosts are seldom sampled from a given locality to distinguish between true rickettsial hosts and incidental vectors (discussed above). *R*. *bellii* seems to parasitize the widest range of tick genera [17], while of the pathogenic taxa, only *R*. *conorii* seems to be limited to one vector species [129]. OrthoMCL predicted one non-representative (RiOG_866) and three representative (RiOG_1005, RiOG_1012 and RiOG_1021) OGs specific to the tick-associated rickettsial genomes (**Figure 5**, **Figure 7**). RiOG_866 is an alpha-(1,3)-fucosyltransferase that is highly truncated in all but the *R*. *bellii* genomes and further split in *R*. *conorii* and *R*. *sibirica* (**Table S1**), depicting a gene undergoing decay. Similarly, RiOG_1021, annotated as a poly-beta-hydroxybutyrate polymerase, is also experiencing pseudogenization, as it depicts an artifact of the clustering process. RiOG_1021 is related to RiOG_834 (core distribution), which has full-length (∼583 aa) proteins in TG and TRG rickettsiae, but parts of split genes from the tick-associated taxa. The corresponding halves of these split genes constitute RiOG_1021. Thus, if only the full sized ORFs are functional, alpha-(1,3)-fucosyltransferase is the lone signature protein found exclusively in TG and TRG genomes (the converse of the tick-associated rickettsiae).

RiOG_1005 has mild similarity to fic (filamentation induced by cAMP) proteins (**Table 12**), which are involved in cell division and folate metabolism (IPR003812). Aside from *R*. *canadensis*, which is highly truncated, the rickettsial sequences contain the central conserved HPFXXGNG motif characteristic of this protein family. Critical for the production and maintenance of new cells [130], folate is especially important during periods of rapid cell division and growth. While the exact molecular function of fic proteins is unknown, it is possible RiOG_1005 is involved in some aspect of folate synthesis, an incomplete pathway in *Rickettsia* likely requiring energy-coupled transporters to uptake host stores of the vitamin and/or its derivatives [61], [62]. However, the absence of this gene in insect- and mite-associated rickettsial genomes and the loss of the majority of the protein in *R*. *canadensis* hint more toward the decaying of this gene family. The identification of a core rickettsial transporter involved in folate/folate derivative uptake would support this hypothesis.

**Table 12:** Results of BLASTP searches evaluating two OGs (RiOG_1005 and RiOG_1012) predicted by OrthoMCL to contain only tick-associated rickettsiae.

RiOG_1005			
Accession No.	Taxon/annotation^A^	score (bits)	E value
**YP_538109**	***Rickettsia bellii*** ** RML369-C; Cell filamentation protein Fic**	**636**	**0**
**ZP_01379987**	***Rickettsia bellii*** ** OSU 85-389; HP RbelO_01000779**	**634**	**2.00E-180**
**NP_360166**	***Rickettsia conorii*** ** str. Malish 7; similarity to cell filamentation proteins (fic)**	**518**	**2.00E-145**
**ZP_00142033**	***Rickettsia sibirica*** ** 246; hypothetical cell filamentation proteins (fic)**	**517**	**4.00E-145**
**ZP_00153572**	***Rickettsia rickettsii*** **; COG3177: Uncharacterized conserved protein**	**514**	**2.00E-144**
ZP_01254701	*Psychroflexus torquis* ATCC 700755; HP P700755_13960	286	8.00E-76
ZP_01048880	*Cellulophaga* sp. MED134cell filamentation protein-like (fic)	286	1.00E-75
ZP_01202287	Flavobacteria bacterium BBFL7putative cell filamentation protein Fic	285	2.00E-75
YP_860923	*Gramella forsetii* KT0803; filamentation induced by cAMP (Fic) family protein	275	4.00E-72
NP_973239	*Treponema denticola* ATCC 35405; Fic family protein	242	2.00E-62
YP_378503	*Chlorobium chlorochromatii* CaD3; Fic family protein	241	4.00E-62
YP_790458	*Pseudomonas aeruginosa* UCBPP-PA14; HP PA14_28800	236	2.00E-60
ABQ20395	*Vibrio cholerae* O395; Fic family protein	234	6.00E-60
YP_388986	*Desulfovibrio desulfuricans* G20; HP Dde_2494	232	2.00E-59
YP_901526	*Pelobacter propionicus* DSM 2379; transcriptional regulator, Fis family	228	4.00E-58
ZP_01673548	Candidatus *Desulfococcus oleovorans* Hxd3; conserved HP	226	2.00E-57
YP_064910	*Desulfotalea psychrophila* LSv54; HP DP1174	224	5.00E-57
NP_603868	*Fusobacterium* n. *nucleatum* ATCC 25586; Huntington interacting Protein HYPE	221	5.00E-56
EDK50213	*Shewanella baltica* OS223; filamentation induced by cAMP protein Fic	218	3.00E-55
YP_064922	*Desulfotalea psychrophila* LSv54; HP DP1186	218	3.00E-55
YP_750639	*Shewanella frigidimarina* NCIMB 400; filamentation induced by cAMP protein Fic	218	3.00E-55
ZP_01704525	*Shewanella putrefaciens* 200; filamentation induced by cAMP protein Fic	218	5.00E-55
ABA87022	*Vibrio cholerae*; HP	216	2.00E-54
YP_847967	*Syntrophobacter fumaroxidans* MPOB; filamentation induced by cAMP protein Fic	212	2.00E-53
ZP_00143967	*Fusobacterium nucleatum* subsp. *vincentii* ATCC 49256; hypothetical cytosolic protein	206	1.00E-51
NP_931130	*Photorhabdus luminescens* subsp. *laumondii* TTO1; HP plu3930	202	2.00E-50
YP_516911	*Desulfitobacterium hafniense* Y51; HP DSY0678	196	2.00E-48
NP_634630	*Methanosarcina mazei* Go1; HP MM2606	118	5.00E-25
ZP_00121191	*Bifidobacterium longum* DJO10A; COG3177: uncharacterized conserved protein	92.4	3.00E-17
ZP_01347715	*Rickettsia canadensis* str. McKiel; HP RcanM_01000664	92	4.00E-17
NP_695410	*Bifidobacterium longum* NCC2705; narrowly conserved HP	89	3.00E-16
YP_064096	*Desulfotalea psychrophila* LSv54; HP DP0360	88.6	4.00E-16
YP_001112298	*Desulfotomaculum reducens* MI-1; filamentation induced by cAMP protein Fic	88.2	5.00E-16
YP_001048169	*Methanoculleus marisnigri* JR1; filamentation induced by cAMP protein Fic	85.9	3.00E-15
YP_155075	*Idiomarina loihiensis* L2TR; Uncharacterized protein containing Fic domain	85.5	4.00E-15
YP_001213658	*Dehalococcoides* sp. BAV1; filamentation induced by cAMP protein Fic	82.4	3.00E-14
XP_972015	*Tribolium castaneum*; PREDICTED: similar to CG9523-PA	82	4.00E-14
NP_396283	*Agrobacterium tumefaciens* str. C58; HP AGR_pAT_503	80.9	9.00E-14
NP_268827	*Streptococcus pyogenes* M1 GAS; HP SPy0558	80.5	1.00E-13
YP_281824	*Streptococcus pyogenes* MGAS5005; hypothetical cytosolic protein	80.1	1.00E-13
ZP_01199959	*Xanthobacter autotrophicus* Py2; conserved HP PA0574	80.1	2.00E-13
NP_714132	*Leptospira interrogans* serovar Lai str. 56601;Huntingtin interacting protein E-like protein	80.1	2.00E-13

RiOG_1012			
Accession No.	Taxon/annotation^A^	score (bits)	E value
**NP_360191**	***Rickettsia conorii*** ** str. Malish 7; similarity to acetyltransferase**	**253**	**2.00E-66**
**ZP_00142008**	***Rickettsia sibirica*** ** 246; hypothetical acetyltransferases**	**248**	**1.00E-64**
**ZP_00153597**	***Rickettsia rickettsii*** **; COG0110: Acetyltransferase (isoleucine patch superfamily)**	**242**	**5.00E-63**
AAA86871	*Staphylococcus aureus*; VAT B	64.3	2.00E-09
NP_713859	*Leptospira interrogans* serovar Lai str. 56601; Probable macrolide acetyltransferase	62.4	7.00E-09
YP_517378	*Desulfitobacterium hafniense* Y51; HP DSY1145	59.7	4.00E-08
ZP_01621907	*Lyngbya* sp. PCC 8106; acetyltransferase	59.3	6.00E-08
ZP_01370449	*Desulfitobacterium hafniense* DCB-2; transferase hexapeptide repeat	58.9	7.00E-08
YP_600714	*Streptococcus pyogenes* MGAS2096; Virginiamycin A acetyltransferase	58.5	9.00E-08
NP_441499	*Synechocystis* sp. PCC 6803; acetyltransferase	58.2	1.00E-07
ZP_01547157	*Stappia aggregata* IAM 12614; streptogramin A acetyl transferase	57.8	2.00E-07
ZP_01227803	*Aurantimonas* sp. SI85-9A1; acetyltransferase	57.8	2.00E-07
YP_001182985	*Shewanella putrefaciens* CN-32; transferase hexapeptide repeat containing protein	57.8	2.00E-07
NP_624622	*Streptomyces coelicolor* A3(2); acetyltransferase	57.8	2.00E-07
YP_001038094	*Clostridium thermocellum* ATCC 27405; HP Cthe_1678	57.4	2.00E-07
YP_870325	*Shewanella* sp. ANA-3; streptogramin A acetyl transferase	57.4	2.00E-07
YP_001049952	*Shewanella baltica* OS155; transferase hexapeptide repeat containing protein	57.4	2.00E-07
EDK51061	*Shewanella baltica* OS223;transferase hexapeptide repeat containing protein	57.4	3.00E-07
ZP_01434328	*Shewanella baltica* OS195; transferase hexapeptide repeat	57	3.00E-07
NP_717373	*Shewanella oneidensis* MR-1; streptogramin A acetyl transferase	56.2	5.00E-07
ZP_01595082	*Marinomonas* sp. MWYL1; streptogramin A acetyl transferase	56.2	5.00E-07
ZP_01439726	*Fulvimarina pelagi* HTCC2506; streptogramin A acetyl transferase	55.8	6.00E-07
AAK96241	*Enterococcus hirae*; streptogramin A acetyltransferase	55.8	7.00E-07
AAK91782	*Enterococcus faecium*; streptogramin A acetyltransferase	55.8	7.00E-07
AAK91783	*Enterococcus faecium*; streptogramin A acetyltransferase	55.8	7.00E-07
NP_783842	*Lactobacillus fermentum*; streptogramin A resistance protein	55.5	8.00E-07
AAG21695	*Enterococcus faecium*; streptogramin A acetyltransferase	55.1	1.00E-06
YP_001175130	*Enterobacter* sp. 638; streptogramin A acetyl transferase	55.1	1.00E-06
YP_738635	*Shewanella* sp. MR-7; streptogramin A acetyl transferase	55.1	1.00E-06
NP_385342	*Sinorhizobium meliloti* 1021; PUTATIVE ACETYLTRANSFERASE PROTEIN	54.7	1.00E-06
YP_702166	*Rhodococcus* sp. RHA1; probable chloramphenicol O-acetyltransferase	54.7	1.00E-06
ZP_01706265	*Shewanella putrefaciens* 200; transferase hexapeptide repeat	54.7	2.00E-06
YP_964018	*Shewanella* sp. W3-18-1; transferase hexapeptide repeat containing protein	54.7	2.00E-06
YP_051279	*Erwinia carotovora* subsp. *atroseptica* SCRI1043; streptogramin A acetyl transferase	54.3	2.00E-06
ZP_01691225	*Microscilla marina* ATCC 23134; virginiamycin A acetyltransferase	54.3	2.00E-06
ZP_01780719	*Shewanella baltica* OS185; transferase hexapeptide repeat containing protein	54.3	2.00E-06
ZP_01186630	*Bacillus weihenstephanensis* KBAB4; transferase hexapeptide repeat	54.3	2.00E-06
ZP_01613073	*Alteromonadales bacterium* TW-7; streptogramin A acetyl transferase	53.9	2.00E-06
YP_927261	*Shewanella amazonensis* SB2B; streptogramin A acetyl transferase	53.9	2.00E-06
YP_171536	*Synechococcus elongatus* PCC 6301; acetyltransferase	53.9	3.00E-06
NP_978962	*Bacillus cereus* ATCC 10987; acetyltransferase, CYSE/LACA/LPXA/NODL family	53.9	3.00E-06
NP_832369	*Bacillus cereus* ATCC 14579; Virginiamycin A acetyltransferase	53.5	3.00E-06
NP_844991	*Bacillus anthracis* str. Ames; acetyltransferase, CYSE/LACA/LPXA/NODL family	53.5	3.00E-06
AAF63432	*Yersinia enterocolitica*; streptogramin A acetyl transferase	53.1	4.00E-06
AAK91784	*Enterococcus faecium*; streptogramin A acetyltransferase	53.1	4.00E-06
YP_007518	Candidatus *Protochlamydia amoebophila* UWE25; streptogramin A acetyltransferase	53.1	4.00E-06
AAF24171	*Enterococcus faecium*; acetyltransferase SatG	53.1	5.00E-06
ZP_00241060	*Bacillus cereus* G9241; streptogramin A acetyl transferase	53.1	5.00E-06
YP_001214452	*Dehalococcoides* sp. BAV1; Acetyltransferase (Ile patch superfamily)-like protein	52.8	5.00E-06
ZP_00834849	*Yersinia intermedia* ATCC 29909; COG0110: Acetyltransferase (Ile patch superfamily)	52.8	5.00E-06
AAK91785	*Enterococcus faecium*; streptogramin A acetyltransferase	52.8	6.00E-06
ZP_01727521	*Cyanothece* sp. CCY0110; VatB	52.4	7.00E-06
NP_105599	*Mesorhizobium loti* MAFF303099; streptogramin A acetyl transferase	51.6	1.00E-05
EAZ74717	*Vibrio cholerae* NCTC 8457; streptogramin A acetyltransferase (Vat(D)	51.6	1.00E-05
YP_537654	*Rickettsia bellii* RML369-C; Acetyltransferases	51.2	1.00E-05
ZP_00743573	*Bacillus thuringiensis* serovar *israelensis* ATCC 35646; Virginiamycin A acetyltransferase	51.2	2.00E-05
ZP_00909619	*Clostridium beijerincki* NCIMB 8052; acetyltransferase (the Ile patch superfamily)	51.2	2.00E-05
YP_508577	*Jannaschia* sp. CCS1; transferase hexapeptide protein	51.2	2.00E-05
ZP_01379765	*Rickettsia bellii* OSU 85-389; HP RbelO_01000549	50.8	2.00E-05
NP_811297	*Bacteroides thetaiotaomicron* VPI-5482; acetyltransferase	50.4	3.00E-05
YP_895166	*Bacillus thuringiensis* str. Al Hakam; virginiamycin A acetyltransferase	50.4	3.00E-05
AAK91786	*Enterococcus faecium*; streptogramin A acetyltransferase	50.4	3.00E-05
ZP_01803272	*Clostridium difficile* QCD-32g58; HP CdifQ_04002574	50.1	3.00E-05
NP_246134	*Pasteurella multocida* subsp. *multocida* str. Pm70; VatB	50.1	3.00E-05
NP_347413	*Clostridium acetobutylicum* ATCC 824; Acetyltransferase (the Ile patch superfamily)	50.1	4.00E-05
YP_083964	*Bacillus cereus* E33L; virginiamycin A acetyltransferase	50.1	4.00E-05
YP_734654	*Shewanella* sp. MR-4; streptogramin A acetyl transferase	50.1	4.00E-05

A HP.

B Truncated ORF from *R*. *canadensis* (ZP_01347690.1) annotated as HP with bit score = 45.8.

RiOG_1012 is highly similar to macrolide, virginiamycin A, chloramphenicol, and streptogramin A acetyltransferases, acetyltransferases of the isoleucine patch superfamily and transferases with hexapeptide repeats from many different bacterial species, several of which are highly pathogenic (**Table 12**). In particular, streptogramin A and virginiamycin A acetyltransferases confer gram-positive bacteria resistance to A-type compounds of virginiamycin-like (Vml) antibiotics [e.g.], [ 131]–[134]. Because gram-negative bacteria typically have an innate resistance to Vml antibiotics [e.g.], [135,136], the presence of Vml acetyltransferases in certain gram-negative bacterial genomes went unnoticed until their discovery early this decade in *Yersinia enterocolitica* [137]. With the rapid accumulation of bacterial genome sequences it became apparent that many gram-negative bacterial genomes harbor Vml acetyltransferases (e.g., **Table 12**). Interestingly, the predominant presence of Vml acetyltransferases on plasmids in gram-positive bacteria versus their typical chromosomal location in gram-negative bacteria suggests that the genes encoding these variable proteins likely spread via conjugation and possibly equip gram-positive bacteria with resistance to Vml antibiotics [137]. While all six sequences within RiOG_1012 are highly similar in the C-terminal region, the N-terminal halves of the proteins are highly divergent between SFG rickettsiae, the *R*. *belii* sequences, and *R*. *canadensis* (**Table 12**). This is consistent with the initial studies that concluded streptogramin, chloramphenicol and related acetytransferases belong to a vast family of enzymes with varying substrates [131], [138]. The presence of a Vml acetyltransferase only in tick-associated rickettsiae is interesting and implores further laboratory investigation.

As with insect-associated rickettsiae, OrthoMCL predicted few signatures for tick-associated rickettsiae. Despite the diversity between insects and ticks, all of the analyzed rickettsial species are capable of infecting vertebrates; thus the identified host-specific OGs likely do not contain proteins involved in vertebrate host cell invasion and pathogenicity. The likelihood that these signatures are involved in arthropod cell entry is also low, given the incidental collection of rickettsial species outside the range of their expected hosts (discussed above). However, these signature genes may be involved in mechanisms specific to arthropod host lifestyle, aiding long-term infection and the ability to persist in tick (via transstadial and transovarial transmission) and insect (via fecal inoculation and inhalation) populations despite the rapid generation times of these arthropods.

### Plasmid Associated OGs

We recently analyzed the genetic composition of the pRF plasmid of *R*. *felis* and determined that the replicon is composed of genes with likely origins to AG rickettsiae and other plasmid-containing bacteria [28]. This suggests that the last common ancestor of all rickettsiae likely harbored plasmids, with *R*. *bellii* [139], *R*. *felis* [27], likely *R*. *akari* [140] and other members of TRG rickettsiae, and some members of SFG rickettsiae either maintaining plasmids despite the constraints of shrinking genomes, or acquiring plasmids later in their evolution. Given the plasticity of plasmid presence/absence in other obligate intracellular bacteria [[e.g., 141]–[145]], as well as other medically-important pathogenic bacteria [e.g., 146]–[150], it is probable that the presence of plasmids may be variable at the strain level in *Rickettsia*, particularly when only one of the two sequenced *R*. *bellii* genomes harbors a plasmid [9], [139]. Past reports of pulsed-field gel electrophoresis (PGE) on rickettsial species that do not correlate with the sizes of recently sequenced genomes [151], [152] may also allude to plasmid plasticity in populations of species and strains of *Rickettsia*.

Our previous suspicion that plasmids are likely to be found in some lineages of SFG rickettsiae [28] has recently been confirmed, as the plasmid pRM from *R*. *monacensis* was identified by transposon insertion and further characterization and sequencing [60]. Subsequently, the same research group used PGE and southern blotting to identify plasmids of variable size and composition in *R*. *helvetica*, *R*. *peacockii*, *R*. *amblyommii*, and *R*. *massiliae* [59]. The entire plasmid sequence of *R*. *massiliae* was later reported [70]. Furthermore, the duplication of several ORFs associated with the type IV secretion system (T4SS) in rickettsiae (VirB4, VirB6, VirB8, and VirB9), coupled with phylogenetic evidence for an ancestral plasmid origin of all T4SSs [153], suggests plasmid systems and related chromosomal genes are a major constituent of rickettsial genomes, possibly contributing to pathogenicity in many lineages. The recent discovery of extraordinarily duplicated conjugative operons, as well as extremely elevated levels of transposons, TPR and ANK motif-containing proteins, integrases, and potential T4SS effector proteins in the *Orientia tsutsugamushi* genome further attests to the phenomena of plasmid plasticity and HGT amongst the Rickettsiales [58], implying that the rickettsiae progenitor was larger and less stream-lined than its modern descendants [32] and likely equipped with a suite of conjugative machineries [28].

#### Plasmids

OrthoMCL grouped 58 predicted pRF ORFs into 49 OGs, with 11 pRF ORFs left as singletons (**Table 13**). Of these 49 OGs, six contained two pRF ORFs (RiOG_920, RiOG_1057, RiOG_1279, RiOG_1282, RiOG_1283, and RiOG_1596), and one contained three pRF ORFs (RiOG_928), depicting the presence of duplicated genes on the plasmid, including the chromosomal replication initiator protein DnaA (pRF04 and pRF19), a probable transposase of the mutator family (pRF01, pRF30 and pRF55), an epsilon subunit-like protein of DNA polymerase III (pRF34 and pRF53), two TPR motif-containing proteins: (pRF12 and pRF15) and (pRF16 and pRF18), a site specific recombinase similar to DNA invertase Pin homologs and TnpR resolvase (pRF32 and pRF66), and a predicted transcription regulatory protein (pRF02 and pRF29). The remaining representative OGs containing single pRF ORFs generally reflect the distribution reported by Gillespie et al. [28] based on BLASTP results, except for a few instances (italicized OGs in **Table 13**). In comparison to the recently discovered SFG rickettsial plasmids, it is apparent that at least three proteins, namely a DnaA-like replication initiation protein, a Sca12-like protein and a small heat shock protein, are common to all rickettsial plasmids [59]. Thus, despite the growing number of plasmids in *Rickettsia*, their unknown origin in the rickettsial tree and lack of conserved genes involved in conjugation keep their exact function and essentiality elusive.

**Table 13:** Distribution of the 68 *R*. *felis* pRF plasmid ORFs within the OGs predicted by OrthoMCL^A^.

ORFs present *exclusively* on the pRF plasmid						
(pRF+pRFδ)						
ORF	Name	Annotation^B^	OG^C^^,^ D ^,^ E	R/N^F^	No. taxa	No. ORFs
pRF04	--------	*R*. *felis* specific protein^G^	1604	R*	1	2
pRF05	--------	chromosomal replication initiator protein DnaA-like protein G	920	N*	6	8
pRF07	*HsdR*	type I restriction-modification system methyltransferase subunit H	1223	R*	3	4
pRF09	--------	*R*. *felis* specific protein (not found in other life) G	1605	R*	1	2
pRF12	*tpr*	tetratricopeptide repeat domain (TPR) G ^,^ I	1279	N*	1	2
pRF14	*ank*	ankyrin-repeat containing gene (ANK) J	1597	R*	1	1
pRF39	--------	MobA_MobL (plasmid transfer)/RecD (exonuclease V) hybrid I ^,^ K	1086	R*	4	4
pRF40	--------	*R*. *felis* specific protein I	1603	R*	1	1
pRF44	*traDF*	putative conjugative transfer protein TraD (*E*. *coli* F plasmid) G	1073	R*	4	4
pRF45	--------	*R*. *felis* specific protein G	1593	R*	1	1
pRF46	*traGF*	putative conjugative transfer protein TraG (*E*. *coli* F plasmid) G	1085	R*	4	4
pRF47	*traGF*	putative conjugative transfer protein TraG (*E*. *coli* F plasmid)	1588	R*	1	1
pRF48	*rve*	integrase (integration of viral DNA into the host chromosome) L	1402	R*	2	2
pRF49	--------	similar to integrase L	1578	R*	1	1
pRF50	--------	HP conserved in a few other bacteria	1582	R*	1	1
pRF53	--------	DNA polymerase III, epsilon subunit-like protein I ^,^ M	1057	N*	1	3
pRF56	--------	hyaluronidase (increases tissue permeability/antigenic disguise) I	1583	R*	1	1
pRF57	*trp_20*	transposase 20: IS116/IS110/IS902 family [pfam02371] N	1591	R*	1	1
pRF58	*trp*	COG3547: transposase and inactivated derivatives N	1585	R*	1	1
pRF59	--------	*R*. *felis* specific protein (not found in other life) G	1580	R*	1	1
pRF60	--------	similar to IS element transposase (*E*. *coli*) G	1592	R*	1	1
pRF62	--------	*R*. *felis* specific protein; possible tldD/PmbA protein I	1589	R*	1	1
pRF63	--------	*R*. *felis* specific protein; similar to *Wolbachia* repA G	1590	R*	1	1
pRF66	--------	site-specific recombinases (DNA invertase Pin homologs) O	1282	N*	1	2
pRF67	--------	similar to transposase ISSag8 (*Streptococcus agalactiae* A909) P	1602	R*	1	1
pRF68	--------	rickettsial HP	1054	R*	5	5

(pRF)						
ORF	Name	Annotation^B^	OG^C^^,^ D ^,^ E	R/N^F^	No. taxa	No. ORFs
pRF15	--------	rickettsial HP G ^,^ I	1279	N*	1	2
pRF20	--------	chromosomal replication initiator protein DnaA-like protein	-------	---	--	--
pRF21	--------	*R*. *felis* specific protein; possible transcription repressor protein	-------	---	--	--
pRF22	--------	similar to *P*. *syringae* plasmid Ppsr1 ORF12	-------	---	--	--
pRF24	*tpr*	tetratricopeptide repeat domain (TPR); similar to sca12	-------	---	--	--
pRF28	--------	rickettsial HP	1497	R	3	3
pRF32	*tnpR*	TnpR resolvase (plasmid-encoded site-specific recombinase) Q	1282	N*	1	2
pRF33	--------	*R*. *felis* specific protein	-------	---	--	--
pRF34	--------	DNA polymerase III, epsilon subunit-like protein; WGR domain I ^,^ M	1057	N*	1	3
pRF36	--------	*R*. *felis* specific protein	-------	---	--	--
pRF37	--------	conjugative transfer protein TraD Ti (*A*. *tumefaciens* Ti plasmid)	1200	R	4	4
pRF38	--------	conjugative transfer protein TraA Ti (*A*. *tumefaciens* Ti plasmid) R	1606	N	1	2

ORFs present on the pRF plasmid *and* the *R*. *felis* chromosome						
(pRF+pRFδ)						
ORF	Name	Annotation^B^	OG^C^^,^ D ^,^ E	R/N^F^	No. taxa	No. ORFs
pRF01	*tnp*	hypothetical transposase (or inactive derivative) G ^,^ S	928	N*	1	5
pRF02	--------	hypothetical transcription regulatory protein I ^,^ T	1283	N*	1	2
pRF03	*parA*	possible cytokinesis regulatory protein	*1610*	R*	1	1
pRF06	*HsdR*	type I restriction-modification system methyltransferase subunit	*1601*	R*	1	1
pRF08	--------	similar to a part of CheY-like receiver domain U	*1579*	R*	1	1
pRF10	--------	rickettsial HP	*1584*	R*	1	1
pRF11	*pat2*	patatin-like phospholipase	*1587*	R*	1	1
pRF13	*tmk*	thymidylate kinase (TMPK)	*1595*	R*	1	1
pRF41	*tnp*	transposase 31: putative transposase, YhgA-like [pfam04754] I	936	R*	6	6
pRF42	*ank*	ankyrin-repeat containing gene (ANK)	*1586*	R*	1	1
pRF43	*traDF*	putative conjugative transfer protein TraD (*E*. *coli* F plasmid) V	1401	R*	2	2
pRF51	*hspP2*	small heat-shock protein 2 W	1280	N*	1	2
pRF52	*hspP1*	small heat-shock protein 1 W	*1594*	R*	1	1
pRF54	*tnp*	transposase, mutator family (transposase_mut) [pfam00872] X	1278	R*	2	2
pRF55	*tnp*	transposase, mutator family (transposase_mut) [pfam00872]^G^^,^ S	928	N*	1	5
pRF61	*tnp*	transposase 31: putative transposase, YhgA-like [pfam04754] I	*1581*	R*	1	1
pRF64	*tnp*	transposase 14 [pfam01710]	1281	N*	1	2
pRF65	*tpr*	tetratricopeptide repeat domain G	*1607*	R*	1	1

(pRF)						
ORF	Name	Annotation^B^	OG^C^^,^ D ^,^ E	R/N^F^	No. taxa	No. ORFs
pRF16	*tpr*	tetratricopeptide repeat domain (TPR) I ^,^ Y	1596	N	2	2
pRF17	*tpr*	tetratricopeptide repeat domain (TPR)	-------	---	--	--
pRF18	*tpr*	tetratricopeptide repeat domain (TPR) I ^,^ Y	1596	N	2	2
pRF19	--------	chromosomal replication initiator protein DnaA-like protein G	920	N*	6	7
pRF23	*parA*	possible cytokinesis regulatory protein	-------	---	--	--
pRF25	*sca12*	cell surface antigen 12	-------	---	--	--
pRF26	*lon*	ATP-dependent protease La, bacterial type (TPR-containing)	-------	---	--	--
pRF27	--------	similar to ABC_SMC_euk (chromosome maintenance) Z	1496	R	3	3
pRF29	--------	rickettsial HP I ^,^ T	1283	N*	1	2
pRF30	*tnp*	transposase, mutator family (transposase_mut) [pfam00872] G ^,^ S	928	N*	1	5
pRF31	*tnp*	COG3328: transposase (or inactive derivative) G	6	N*	1	17
pRF35	*parB*	cleaves ssDNA and supercoiled plasmid DNA	-------	---	--	--

A Results include 44 predicted ORFs from the putative smaller *R*. *felis* plasmid, pRFδ (see Gillespie et al., 2007).

B Follows Gillespie et al. (2007). Additional annotation is listed below in footnotes G-Z.

C Blank values depict *R*. *felis* singletons.

D Bold OGs depict singletons upon the removal of doubtful orthologs from pRFδ. Italicized OGs Blast to chromosomal proteins on the R. *felis* chromosome.

E Underlined OGs contain more than one pRF ORF.

F Representative (R) or non-representative (N) family. Groups containing ORFs from pRFδ are noted with an asterisk.

G HP.

H Type I restriction enzyme EcoEI M protein.

I CHP.

J The pRFδ protein in PATRIC, VBI0166RF3_0019, is slightly different than the pRF protein.

K Conjugal transfer protein TraA.

L Putative transposase.

M DNA polymerase III polC-type

N Transposase for insertion sequence element IS1328.

O R46 site-specific recombinase, Transposon Tn917 resolvase.

P ISBma2, transposase.

Q Transposon Tn917 resolvase, R46 site-specific recombinase.

R The pRFδ protein in PATRIC, VBI0166RF3_0020, is slightly different than the pRF protein.

S Probable transposase for transposon Tn903.

T COG1396: Predicted transcriptional regulators.

U COG3706: Response regulator containing a CheY-like receiver domain and a GGDEF domain.

V Protein virD4.

W Spore protein SP21.

X Probable transposase for insertion sequence element.

Y Cell division cycle protein 27 homolog.

Z Myosin-11 (*R*. *prowazekii*), M protein, serotype 2.1 precursor (*R*. *typhi*).

#### Toxin-antitoxin modules

Many plasmid-containing bacteria have associated toxin-antitoxin (TA) systems encoded on plasmids, typically as two-component operons, for the control of plasmid partitioning and stable inheritance [154]–[157]. Antitoxins, usually highly labile in their mature form, are constitutively expressed and neutralize the accumulation of their counterpart toxins, which are more stable. Upon imperfect segregation of plasmids after cell division, plasmidless daughter cells are destroyed by elevated toxin levels due to the rapid breakdown of the unstable antitoxin and lack of its further synthesis [158], [159]. Although originally described as mediators of bacterial programmed cell death, studies now suggest that TA modules also act as regulators of the stringent response (reviewed in [159]) and are widely present on chromosomes of diverse bacteria [160]. While TA systems are found in many free-living bacteria, they are typically uncommon among obligate intracellular pathogens [160], [161]. However, the genome sequences for both *R*. *bellii* strains and *R*. *felis* contain elevated levels of chromosomally encoded TA loci, the majority of which seem to be degraded [9], [27]. Moreover, these genomes typically retain only one component of the TA modules, possibly alluding to a neofunctionalization [153] of the remaining genes for adaptation to eukaryotic hosts, as has been suggested for at least *R*. *felis* toxin and antitoxin genes [27]. However, given that the reductive nature of rickettsial genomes may result in high levels of constitutively expressed loci and reduced operons, and that many antitoxins contain motifs common to two, three and even four different DNA-binding-proteins [162], incomplete and noncontiguous rickettsial TA modules may still interact with one another to coordinate a response to stress within host cells. Alternatively, the presence of incomplete TA modules may reflect vertically acquired plasmid-associated genes that are in the process of pseudogenization. In support of this, of the numerous TA components in the *R*. *felis* genome, only one VapB antitoxin (RiOG_941) was recovered in a proteome screen [40].

Our bioinformatic analysis reveals that components of 5 TA systems (*relBE*, *phd/doc*, *vapBC/vag*, *mazEF*, and *parDE*) are recurrent in all rickettsial genomes save the TG rickettsiae and *R*. *canadensis* (**Table S4**). Of the predicted 56 toxin and 86 antitoxin ORFs, zero occur in the TG rickettsiae and only two are found in *R*. *canadensis*. The majority of these ORFs occur in the *R*. *bellii* genomes and TRG rickettsiae (avg. of 22 and 26 TA ORFs per genome, respectively), although slightly lower levels also occur in SFG rickettsiae (avg. of 14.3 TA ORFs per genome). However, there are more occurrences of similar TA module components shared between the *R*. *bellii* genomes and TRG rickettsiae (12) than between SFG rickettsiae and either the *R*. *bellii* genomes (1) or TRG rickettsiae (5) (**Table S4**). Thus, the presence and distribution of these TA ORFs correlates to the lineages of sampled rickettsiae that contain plasmids, and further supports the TRG rickettsiae having affinities with AG rickettsiae [28]. Furthermore, the *R*. *belli* and TRG rickettsiae genomes have elevated levels of predicted PIN-domain proteins (homologs of the pilT N-terminal domain), which in eukaryotes function as ribonucleases [163], [164] involved in RNAi and nonsense-mediated RNA degradation [162], [163]. Most of the described prokaryotic PIN-domain proteins are toxins of chromosomally-encoded TA operons [159]–[161] that are present in a diverse array of unrelated bacteria, likely having arisen due to the advantages they bestow on competing mobile elements [165], [166]. Indeed, the PilT protein of the pathogenic *Neisseria meningitidis* has been hypothesized to interact with the T4SS due to its limited homology to the DotB protein of the *Legionella* T4SS [167].

While the exact manner of their origin and current functional significance is debatable, it is apparent that TA systems have arisen via HGT in a wide range of bacteria [168]. Given the distribution of plasmids and associated TA systems in the analyzed rickettsial genomes, it is likely that conjugation via plasmids befits some rickettsial lineages with genes important for survival in stressful environments, allowing for dormancy and slow growth. However, it remains to be determined if those rickettsial species that harbor plasmids use TA modules for mediating the partitioning and stable inheritance of said plasmids.

### Singleton ORFs

**Figure 10:**
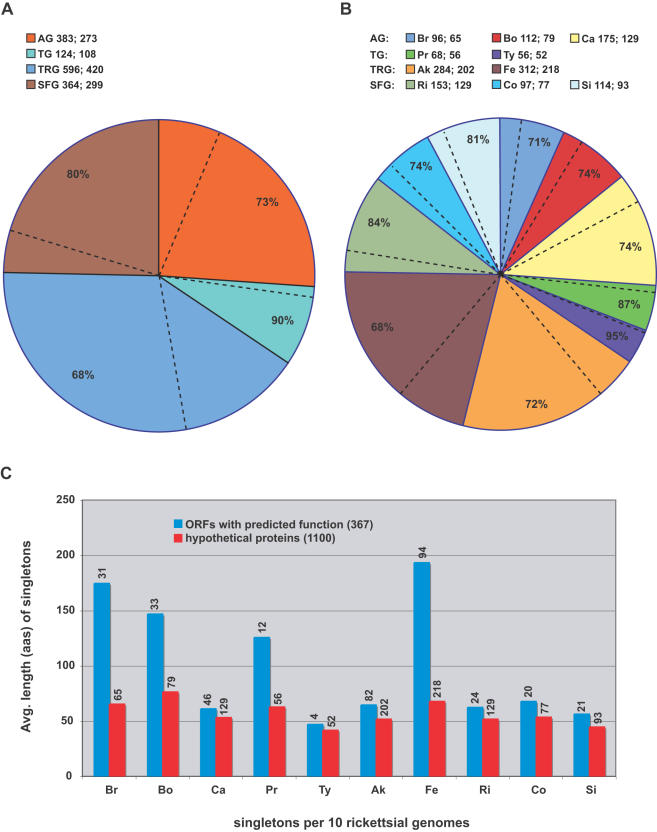
Analysis of the distribution of 1467 singleton ORFs omitted from OG prediction across 10 rickettsial genomes. (A) Singleton ORFs across four rickettsial groups. (B) Singleton ORFs across 10 rickettsial genomes. First number is total number of singleton ORFs per taxon, with second number the total singleton ORFs annotated as HPs. Dashed lines in pie charts separate characterized proteins from HPs, with percentages given only for HPs. (C) Average lengths of singleton ORFs with predicted functions versus singleton ORFs annotated as HPs for all ten analyzed rickettsial genomes.

OrthoMCL failed to group 1467 ORFs (10.2% of total predicted ORFs) from the ten analyzed genomes into any OG (**Figure 5** **,** **Figure 7** **;** **Table S5** **,** **S6** **,** **S7** **,** **S8** **,** **S9** **,** **S10** **,** **S11** **,** **S12** **,** **S13** **,** **S14**). The range across rickettsial groups shows TG genomes contribute the least (8.5%) and TRG genomes contribute the most (41%) to the total count of singletons (**Figure 10A**). The individual genome contributions to the overall singleton count range from 4% (*R*. *typhi*) to 21% (*R*. *felis*), with the rank of all genomes matching the group ranking (TG<SFG<AG<TRG) (**Figure 10A**). However, an inherent bias of these comparisons is difficult to avoid, as OrthoMCL grouped 321 ORFs present only from both *R*. *bellii* genomes (**Figure 5** **,** **Figure 7** **,** **Table S3**). Accounting for these *R*. *bellii* doubletons, the rank and proportion of singletons per rickettsial group is modified: TG (7%)<SFG (20%)<TRG (34%)<AG (39%), and illustrates that TRG and AG genomes are more similar in their number of singleton ORFs relative to TG and SFG rickettsiae. This brings up a practical concern with phylogenomic analysis in that sampling one genome per species (or strain) may not suffice for capturing the true composition of genes within the bacterial population. This is consistent with a recent study that cautioned on the very same idea in relation to vaccine design for *Streptococcus agalactiae*, an organism that has a core genome of approximately 80% across various strains, with the accessory genome quite plastic [169]. Using mathematics, a rather daunting conclusion was reached suggesting even after sampling hundreds of additional *S*. *agalactiae* genome sequences, novel genes would still be added to the accessory genome [169]. Nonetheless, inclusion of the *R*. *bellii* doubletons illustrated the similar composition of singletons in AG and TRG genomes and further adds to the similarities these genomes share as a result of related conjugation systems.

Unsurprisingly, the majority of singleton ORFs are annotated as HPs, ranging from 68% (*R*. *felis*) to 95% (*R*. *typhi*) across the analyzed genomes (**Figure 10A, B**). In an effort to identify the degree of over-prediction of ORFs, we plotted the average lengths of singleton ORFs with predicted functions versus singleton ORFs annotated as HPs for all ten rickettsial genomes (**Figure 10C**). The rationale for this is that the majority of singletons under 100 amino acids in length should be HPs, with many having arisen by chance [100]. Aside from *R*. *felis*, *R*. *prowazekii* and the *R*. *bellii* genomes, there is minimal difference between the average lengths of singletons with predicted functions and singletons annotated as HPs. The much larger average lengths of singletons with predicted functions versus singleton HPs are expected in the *R*. *bellii* and *R*. *felis* genomes, as many of the larger singletons in these genomes are probable products of HGT events (e.g., larger transposases, ANK- and TPR-motif containing proteins). This same pattern in the *R*. *prowazekii* singletons, however, is unexpected, yet is skewed in part due to the presence of several large split ORFs that did not cluster into their respective OGs. While the shorter singleton HPs may have arisen by chance, it is likely that some of them are functional genes that are difficult to homologize with other closely related sequences, given the problems with assessing percent conservation across short sequences with even minimal differences. For instance, small ORFs are found in a variety of protein classes, including ribosomal proteins, transcriptional regulators, chaperonins, thioredoxins, metal ion chelators, proteolipids, stress proteins, nucleases, and mating pheromones [170]. Of the original 299 *Saccharomyces cerevisiae* small ORFs annotated as HPs, 170 have since been assigned cellular functions, with the majority of information coming from laboratory evidence [171]. Given the probable plasticity of the accessory genomes of rickettsial strains (discussed above) and the growing importance small ORFs have garnered in the literature [e.g.], [ 171]–[174], the high number of small singleton HPs in *Rickettsia* should not be ignored. Experimental evidence has confirmed the translation of several small HPs in *R*. *felis* [40] and future microarray data will help lend resolution to this poorly understood characteristic of rickettsial genomes.

### Conclusion

This study analyzed 14354 predicted ORFs from ten rickettsial genomes and generated OGs ranging from two to 31 sequences for 90 percent of the total ORFs. A conserved core rickettsial genome consisting of 731 OGs (51% of total predicted ORFs) was identified, and a phylogeny was estimated from this core genome to allow for subsequent phylogenomic comparison of the remaining accessory genome. This robust phylogeny estimate is congruent with our recent reclassification of rickettsial lineages into four groups [28] and OGs specific to each group provide the first signature genes possibly involved in the phenotypic characteristics defining each group. The unstable phylogenetic position of *R*. *canadensis*, coupled with it only sharing three OGs with the *R*. *bellii* genomes, reflects that the base of the rickettsial tree is poorly defined. However, an unprecedented mode of gene loss was discovered in the lineage spanning *R*. *canadensis* and TG rickettsiae, illustrating that gene signatures alone may not well-characterize specific rickettsial groups, but instead the modes of gene loss (and stricter reliance on host resources) may be the defining features [175]. Given the emerging diversity of *Rickettsia* [16], particularly species associated with medically non-important metazoans and ancestrally related to the pathogenic species analyzed here, the origins of pathogenicity from primitive rickettsial symbionts may not be elucidated without a broader genomic comparison reflective of the overall diversity within the genus.

As a consequence of distinguishing OGs comprising single rickettsial groups (e.g., AG, TG, TRG, and SFG), shared rickettsial groups (subgeneric), plasmid-harboring genomes, and genomes with common arthropod hosts (C1OGs) from OGs with a patchy distribution across the rickettsial tree (C2OGs), two interesting results were obtained. First, C2OGs comprise 31% of all generated OGs, implying a significant portion of the rickettsial accessory genome is comprised of gene decay and laterally acquired genes. Supporting this is the presence of the majority of split ORFs within C2OGs (**Table S1**) and the high proportion of gene families typically associated with the bacterial mobile gene pool in C2OGs (47%) versus the low proportions in C1OGs (5%) and singleton ORFs (4%). Second, the ratio of representative OGs to non-representative OGs is skewed within C1OG distributions (71–29%) but nearly equal in C2OG distributions (56–44%), suggesting that gene duplications (paralogs) and HGT events (xenologs) are more prevalent in C2OGs. Taken collectively, these observations yield the manner in which the rickettsial genomes have acquired their variation: a conserved core genome is supplemented with a highly variable accessory genome that is comprised of gene decay and many horizontally acquired genes. However, the nature of the horizontally acquired genes remains unknown: for example, did the products of HGT arise ancestrally in the analyzed taxa, becoming shuffled over time through recombination and high rates of decay, or are HGT products continually sculpting the variation within the accessory genome overtop of a highly reductive nature of all genes within the genome? The recent explosion of reported cases of plasmids in all rickettsial groups except TG rickettsiae argues for the latter scenario, and is congruent with our findings of nearly zero instances of plasmid associated genes, genes typical of HGT events and gene duplications within TG rickettsial genomes. Thus, while many *Rickettsia* seem to be able to accept and pass genes of the mobile gene pool, the contribution of HGT products to pathogenicity is unknown and seemingly nonessential to all known rickettsial pathogens. The role lineage specific virulence factors play in pathogenic strains is thus an important aspect of future laboratory work. While HGT was traditionally considered rare in *Rickettsia*, we recently suggested, based on a detailed analysis of the *R*. *felis* pRF genes, that it is more common, particularly among species in which conjugation systems had yet been discovered [28]. Our suspicions have recently been verified [70] and the exact degree HGT contributes to rickettsial diversification will only be elicited with the accumulation of more rickettsial genome sequences. Such endeavors will challenge our existing classification scheme; however, a preliminary analysis of two recently published SFG rickettsiae genomes (*R*. *massiliae* str. MTU5 and *R*. *africae* str. ESF 5) using genome alignment (**Figure S1-E**) and phylogeny estimation (**Figure S3**) does not overturn our results, and we predict that OGs generated with the inclusion of these new genomes will not alter the conclusions reached herein.

Finally, we present two concerns regarding phylogenomic analysis of *Rickettsia*. First, the high degree of pseudogenization in rickettsial genomes means that OG prediction programs and related methods alone are insufficient for grouping related genes. Manual inspection of algorithm output is imperative, as the high occurrence of split genes will lead to overestimation of non-representative OGs as well as inaccuracies in ORF clustering (see **Table S1**). Second, and perhaps more pressing, is the revelation that rickettsial species may be comprised of highly variable genomes, particularly across exceedingly divergent strains. Attesting to this, our analysis of predicted OGs included two strains of *R*. *bellii* that shared 321 species-specific genes but contained 97 (str. RML369-C) and 117 (str. OSU 85 389) strain-specific genes. Similarly, a recent genomic comparison of *R*. *rickettsii* str. Sheila Smith CWPP with the avirulent *R*. *rickettsii* str. Iowa revealed 143 deletions and 492 SNPs between the two genomes [176]. Altogether, these issues challenge future genomic studies on *Rickettsia*, particularly regarding which species/strains to select for genome sequencing, but also for justifying approaches for vaccine design with little understanding of *what exactly are* rickettsial virulence factors. The complexity *Rickettsia* has posed on laboratory work has plagued researchers for decades, and it is apparent from our study that genomic comparison is not immune from these associated difficulties.

## Materials and Methods

### Gene and protein prediction

Complete protocols for manual and automated curation and annotation of predicted rickettsial ORFs are listed at the PATRIC website (http://patric.vbi.vt.edu/about/standard_procedures.php). The number of ORFs per rickettsial genome differ from the previously published studies (**Figure 7**).

### Generation of orthologous groups

Complete lists (in FASTA format) of all predicted proteins encoded by each of the ten analyzed rickettsial genomes were used as templates for evaluating the performance of a suite of OG prediction methods. All methods began with all-vs-all BLASTP [177], [178] of the complete protein set. The OrthoMCL program [34], a graph-based clustering method centered on the Markov clustering algorithm of Van Dongen [33], was compared with other clustering methods. A reciprocal-best-hit clustering was performed, in which the blast results were first filtered for reciprocal best hits. In the resulting OGs, each member was the reciprocal-best-hit of each other member. Another method used these reciprocal-best-hit clusters as seed groups, which were augmented using Hidden Markov Model (HMM) searches of the complete protein set. A comparison of the resulting OG sets indicated superior performance by OrthoMCL, using the criteria of least number of ungrouped singleton ORFs and most number of OGs with perfect representation (10 ORFs from 10 genomes). Files containing all results from OrthoMCL are posted on PATRIC (http://patric.vbi.vt.edu/about/publications.php).

### Phylogeny estimation

Rickettsial protein sequences comprising the 731 core representative OGs (dataset 1) were exported from the PATRIC database and aligned locally using default parameters in the command-line version of the program MUSCLE [179], [180]. Related sequences from *Wolbachia* (*Drosophila melanogaster* symbiont) were included when possible. Alignments were analyzed under maximum likelihood using Bayesian inference in the program MrBayes v3.1.2 [181]. A starting tree was generated with BIONJ using the WAG amino acid substitution matrix [182] and estimating all parameters with four substitution rate categories [183]. This tree was used to prime the Bayesian analysis, which was run in model-jumping mode with a single chain implemented, assessing burn-in (arrival at a likelihood plateau) as described previously [184]. We also analyzed the data under parsimony in an exhaustive search in the program PAUP* version 4.10 (Altivec) [185]. Branch support was assessed using the bootstrap [186] with default settings in PAUP*. We performed one million bootstrap replications. Tree files from both Bayesian and parsimony analyses were used to draw trees in PAUP*.

The second phylogenetic analysis (dataset 2) incorporating additional rickettsial taxa for which a genome sequence is not available (*R*. *helvetica*, *R*. *australis*) was initiated by performing BLASTP searches against the NCBI protein database using the following 16 *R*. *helvetica* amino acid sequences as queries: citrate synthase I (Q59741; RiOG_175), ATP synthase F1 alpha subunit (AAM93518; RiOG_208), type IV secretion/conjugal transfer ATPase, VirB4 family (ABG74480; RiOG_225), DNA polymerase III alpha subunit (CAB56077; RiOG_230), DNA polymerase I (Q9RLB6; RiOG_231), signal recognition particle-docking protein FtsY (CAB56072; RiOG_232), recombinase A (ABG74458; RiOG_245), translation elongation factor Tu (Q8KT99; RiOG_305), 10 kDa chaperonin 5 (GroES) (ABD93985; RiOG_335), chaperonin GroEL (ABD93984; RiOG_336), chromosomal replication initiator protein DnaA (ABG74394; RiOG_356), antigenic heat-stable 120 kDa protein Sca4 (AAL23857; RiOG_432), chaperone protein DnaK (ABG74418; RiOG_667), DNA-directed RNA polymerase, beta subunit (AAM93506; RiOG_701), translation elongation factor G (Q8KTB4; RiOG_708), and outer membrane autotransporter barrel domain (190 KD antigen precursor *sca1*) (AAU06440; RiOG_797). The nr (All GenBank+RefSeq Nucleotides+EMBL+DDBJ+PDB) database was used, coupled with a search against the Conserved Domains Database. Searches were performed across ‘all organisms’ with composition-based statistics. No filter was used. Default matrix parameters (BLOSUM62) and gap costs (Existence: 11 Extension: 1) were implemented, with an inclusion threshold of 0.005. Subjects from the ten genomic sequences were retrieved from BLAST results with the *R*. *helvetica* query sequences. When available (8 out of 16) sequences for *R*. *australis* were also retrieved (**Table S15**). Fasta-formatted sequence files were aligned using MUSCLE, with aligned datasets converted to Nexus format using the program seqConverter.pl, version 1.1 [187]. Each Nexus file was concatenated manually into a combined executable Nexus file and analyzed under parsimony in a heuristic search implementing 500 random sequence additions saving 100 trees per replicate. Branch support was assessed from 1000 bootstrap replications.

The third phylogenetic analysis (dataset 3) used the same query sequences as the second analysis but performed tBLASTN [188] searches against the NCBI whole-genome shotgun reads (wgs) database to retrieve homologous sequences from the unannotated *R*. *massiliae* and *R*. *africae* genomes. Parameters were the same as used in the BLASTP searches, and the data were aligned and analyzed in the same manner as the second phylogenetic analysis.

### Genome alignment

Six genome sequence alignments were performed using Mauve v.2.0.0 [189]. Unmodified Fasta files for each rickettsial genome were used as input, except that the *R*. *sibirica* genome sequence was reindexed using the reverse-complement of its circular permutation from the original position 668301.

## Supporting Information

Figure S1 Analysis of synteny across aligned rickettsial genomes. Taxon abbreviations are explained in the Figure 1 legend. Five alignments are shown that are all permutations of the alignment presented in Figure 2. (A) Removal of R. felis. (B) Swapping of R. felis and R. akari. (C) Swapping of the R. bellii genomes. (D) Swapping of the R. bellii genomes plus the repositioning of the R. canadensis genome between TG and TRG rickettsiae. (E) Inclusion of the recently sequenced genomes of R. massiliae str. MTU5 and R. africae str. ESF 5, both SFG rickettsiae. Alignments performed using Mauve (Darling et al., 2004) (see text for details).
(1.99 MB PDF)

Figure S2 Distribution of 637 representative and non-representative class 2 OGs (C2OGs) over estimated rickettsial phylogeny. These OGs likely include pseudogenes, genes with less conserved functions in rickettsiae, and laterally acquired genes. Black = strictly representative OGs, blue = strictly non-representative OGs, red = both representative and non-representative OGs. Top numbers depict total number of OGs and bottom numbers show proportion of hypothetical proteins. Numbers in parentheses depict the proportion of non-representative OGs made representative via concatenation of split ORFs (see Table S1). Asterisks denote distributions that are made entirely representative after split ORF concatenation (27 of 47 non-representative distributions; see Table 4 and Table S1).
(2.99 MB PDF)

Figure S3 Phylogenetic analysis of 14 rickettsial taxa. Tree estimated using the same 16 proteins as the analysis in Figure 9, with the addition of orthologous sequences from the recently completed genomes of R. massiliae str. MTU5 and R. africae str. ESF 5 (sequences obtained from WGS reads using tBlastn). Tree estimated under parsimony (see text for details).
(0.34 MB PDF)

Table S1 Phylogenetic analysis of 14 rickettsial taxa. Tree estimated using the same 16 proteins as the analysis in Figure 9, with the addition of orthologous sequences from the recently completed genomes of R. massiliae str. MTU5 and R. africae str. ESF 5 (sequences obtained from WGS reads using tBlastn). Tree estimated under parsimony (see text for details).
(0.34 MB PDF)

Table S2 Distribution and characterization of predicted ORFs within 259 non-representative OGs across ten rickettsial genomes, and the results after manual curation.
(0.18 MB PDF)

Table S3 Seven hundred-fifty two core rickettsial OGs predicted across ten analyzed genomes.
(0.19 MB PDF)

Table S4 OGs present only in the R. bellii genomes.
(0.06 MB PDF)

Table S5 Distribution of putative toxin-antitoxin (TA) systems within the rickettsial OGs predicted by OrthoMCL.
(0.07 MB PDF)

Table S6 Singletons present in the R. bellii str. RML369-C genome.
(0.05 MB PDF)

Table S7 Singletons and false singletons present in the R. bellii str. OSU 85 389 genome.
(0.06 MB PDF)

Table S8 Singletons and false singletons present in the R. canadensis str. McKiel genome.
(0.06 MB PDF)

Table S9 Singletons present in the R. prowazekii str. Madrid E genome.
(0.05 MB PDF)

Table S10 Singletons present in the R. typhi str. Wilmington genome.
(0.05 MB PDF)

Table S11 Singletons and false singletons present in the R. akari str. Hartford genome.
(0.07 MB PDF)

Table S12 Singletons and false singletons present only in the R. felis genome.
(0.08 MB PDF)

Table S13 (0.06 MB PDF)

Table S14 (0.05 MB PDF)

Table S15 (0.05 MB PDF)
