# Supplementary figures and images for: Rickettsia Phylogenomics: Unwinding the Intricacies of Obligate Intracellular Life
Source: PLoS One. 2008 Apr 16;3(4):e2018. doi: 10.1371/journal.pone.0002018 (PMC2635572; doi:10.1371/journal.pone.0002018)

A

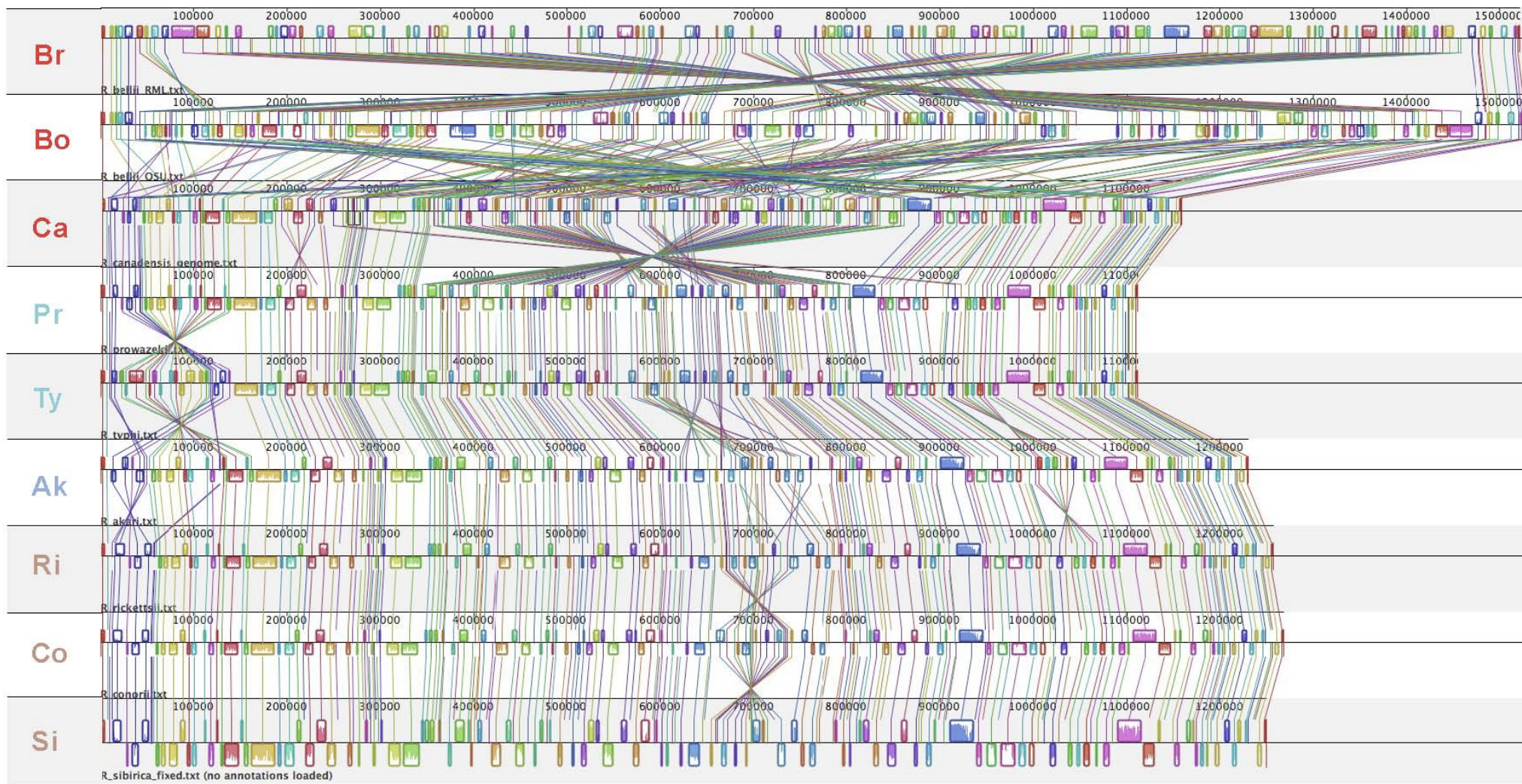

Figure S1-A

**B**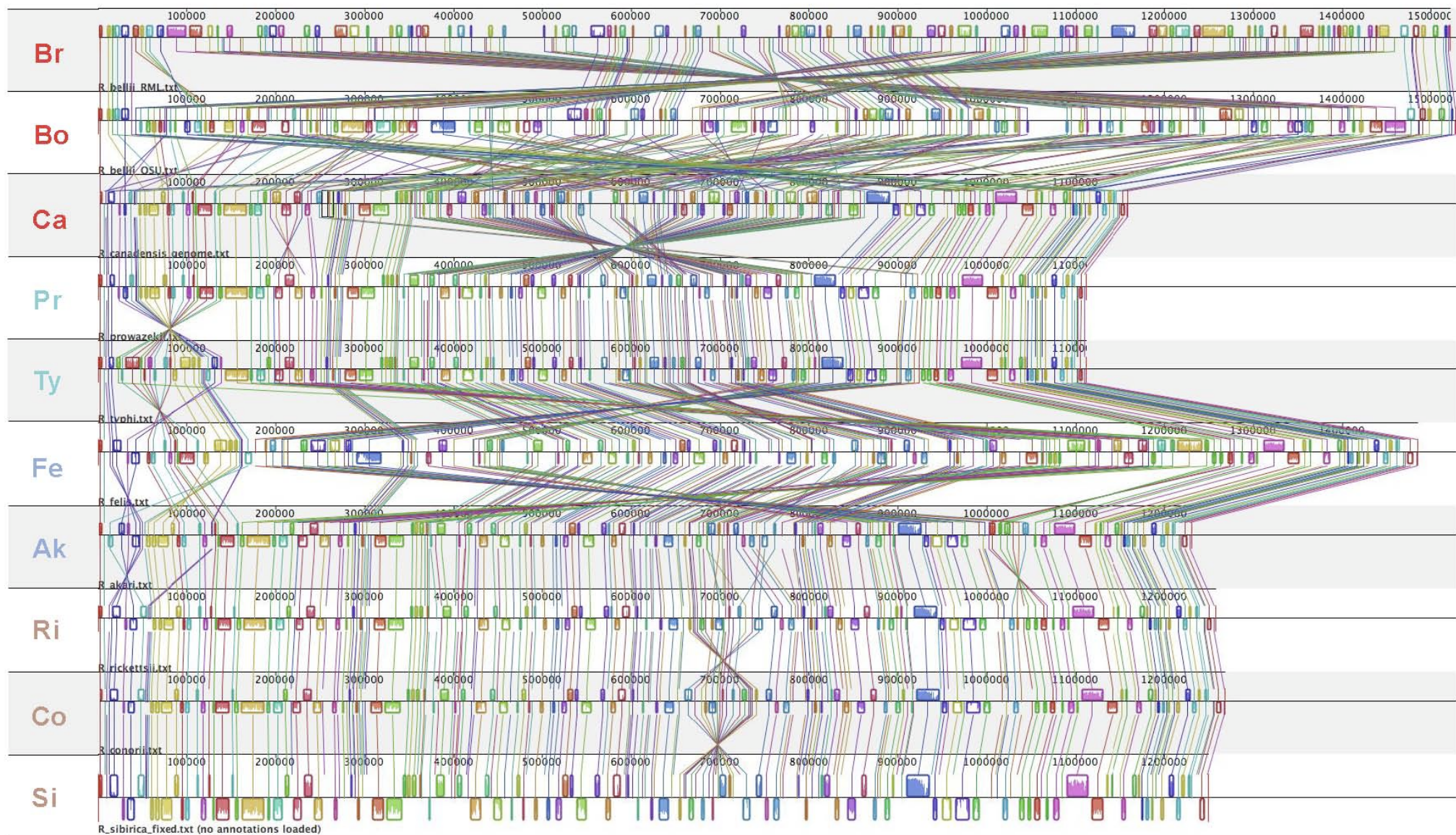

Figure S1-B

C

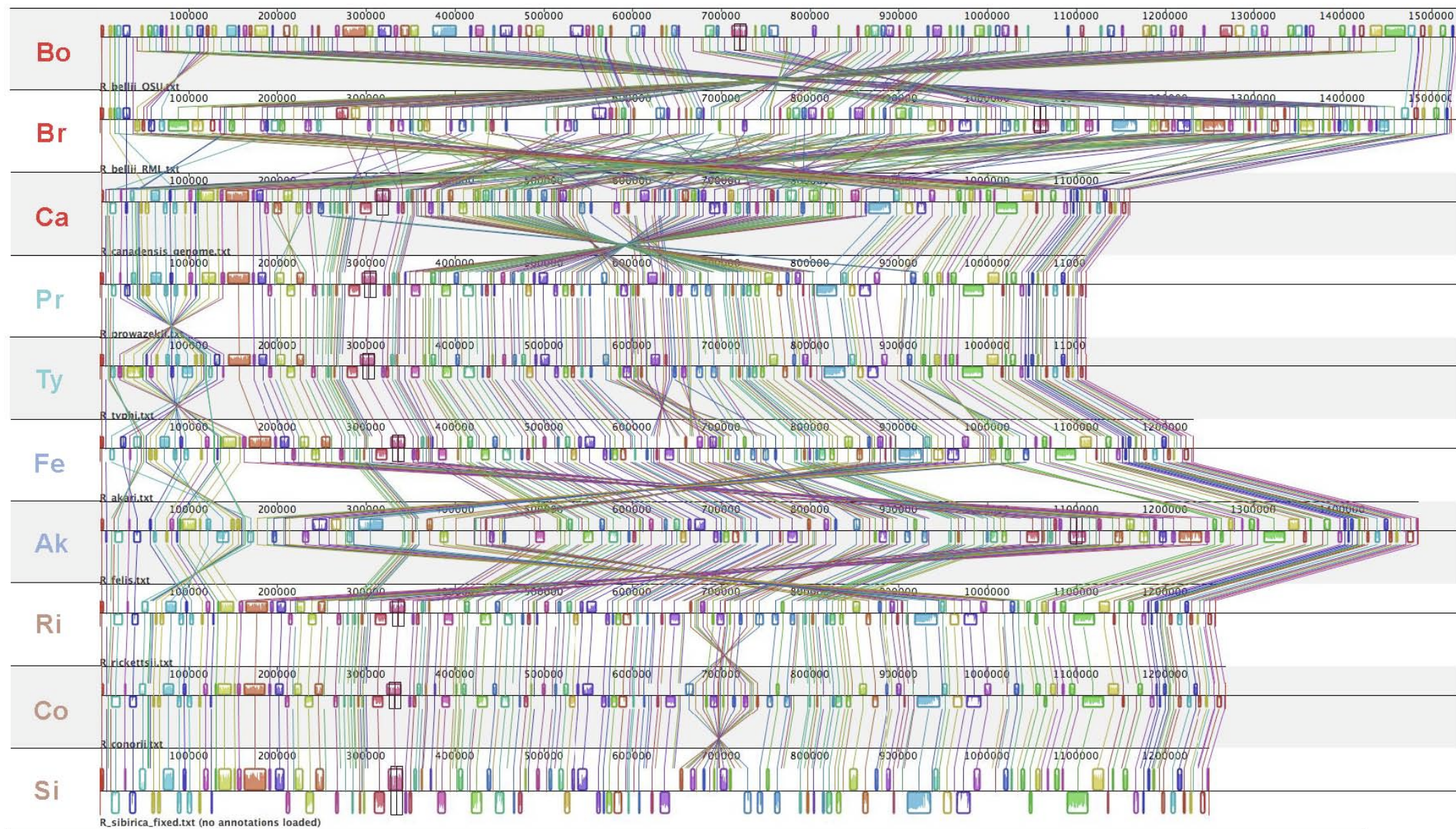

Figure S1-C

D

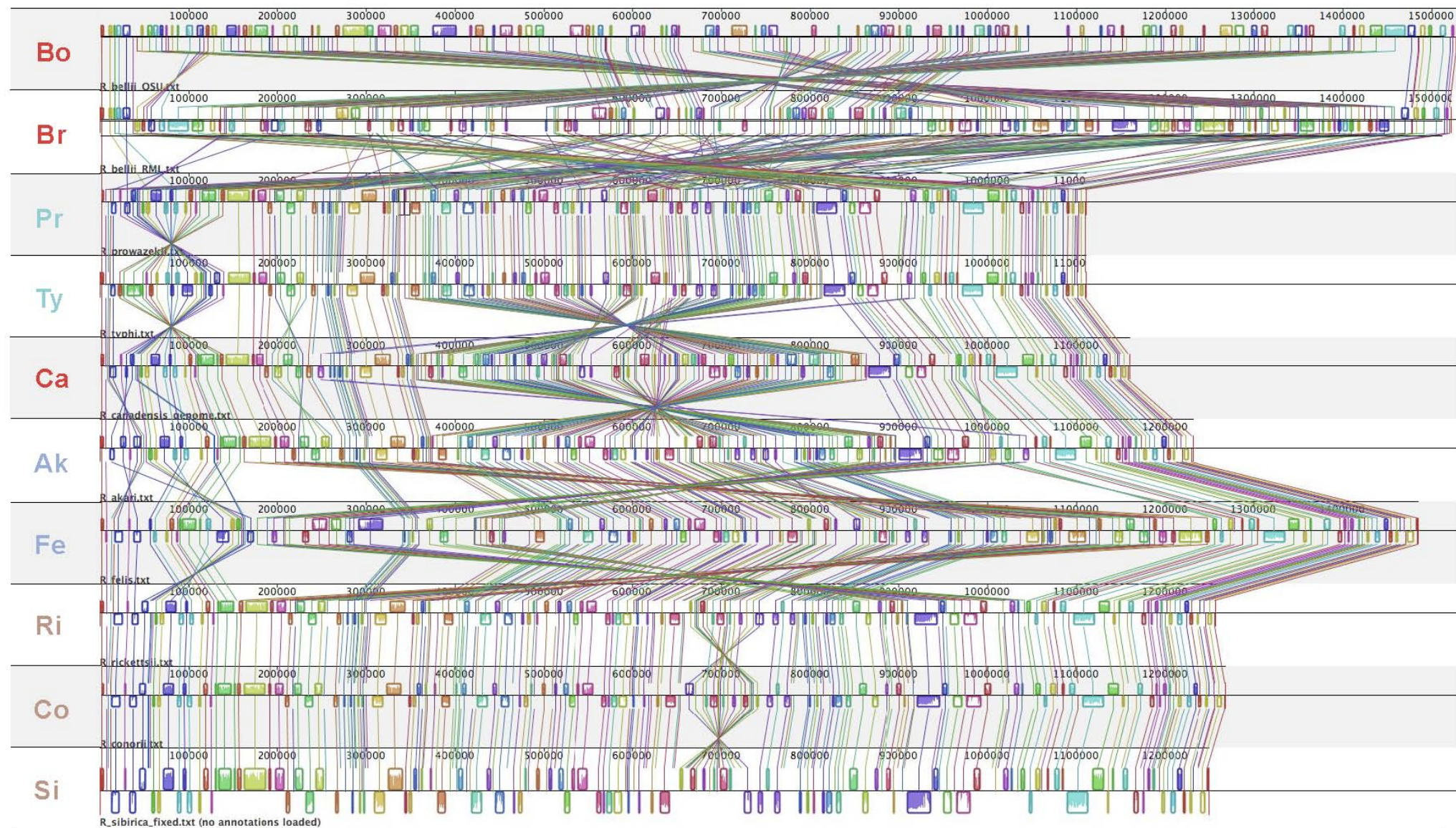

Figure S1-D

E

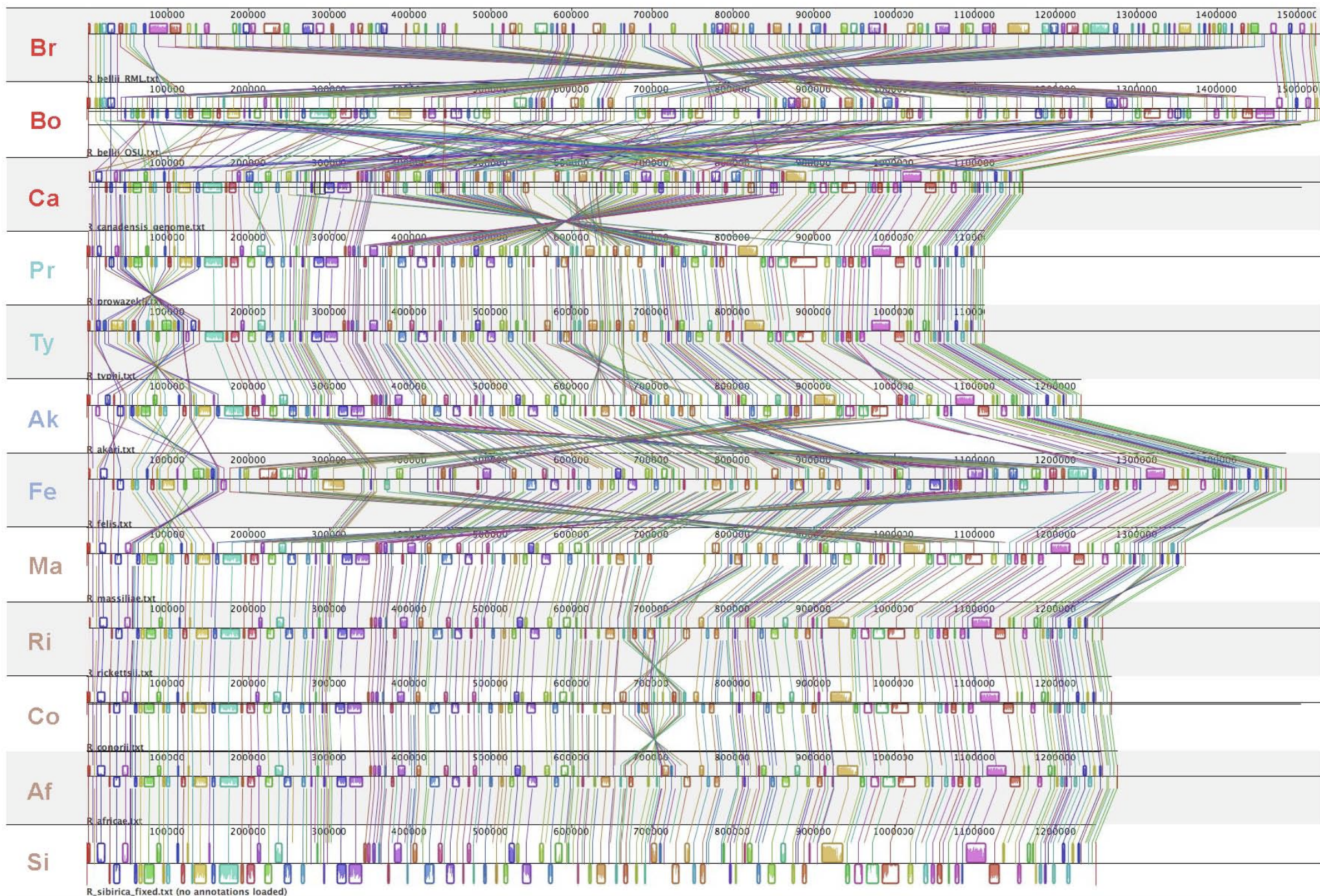

Figure S1-E

Supplement: Figure S1 — Analysis of synteny across aligned rickettsial genomes. Taxon abbreviations are explained in the Figure 1 legend. Five alignments are shown that are all permutations of the alignment presented in Figure 2. (A) Removal of R. felis. (B) Swapping of R. felis and R. akari. (C) Swapping of the R. bellii genomes. (D) Swapping of the R. bellii genomes plus the repositioning of the R. canadensis genome between TG and TRG rickettsiae. (E) Inclusion of the recently sequenced genomes of R. massiliae str. MTU5 and R. africae str. ESF 5, both SFG rickettsiae. Alignments performed using Mauve (Darling et al., 2004) (see text for details). (1.99 MB PDF) [file pone.0002018.s001.pdf]

Figure S2

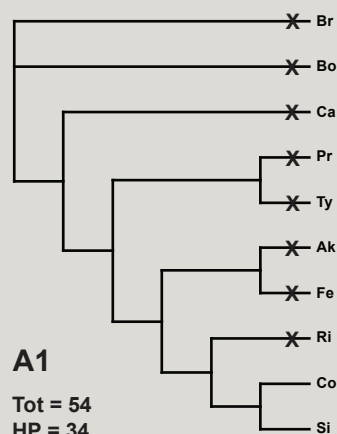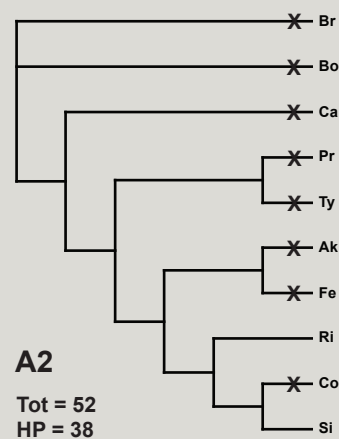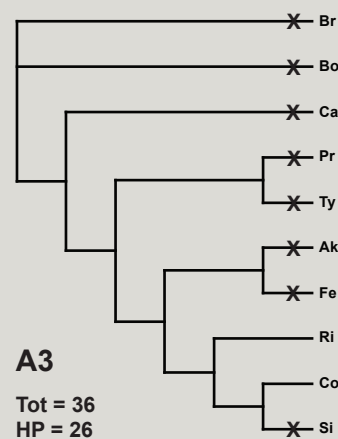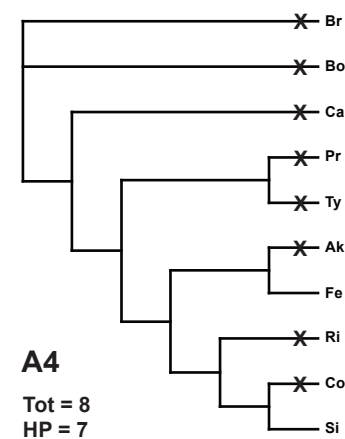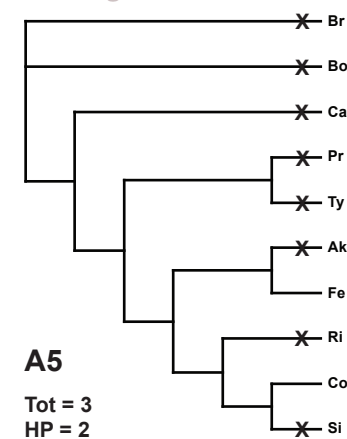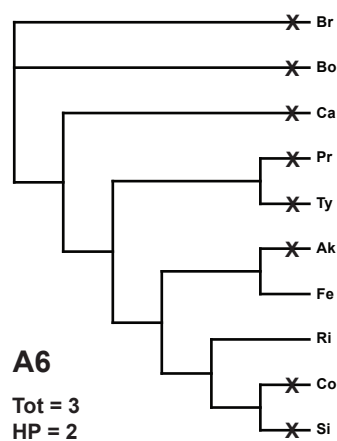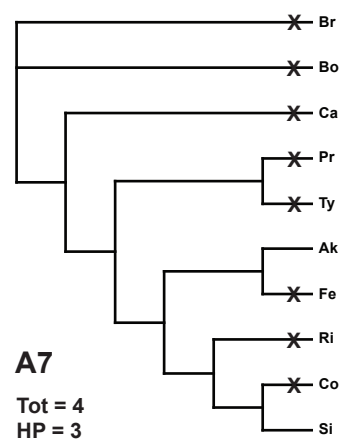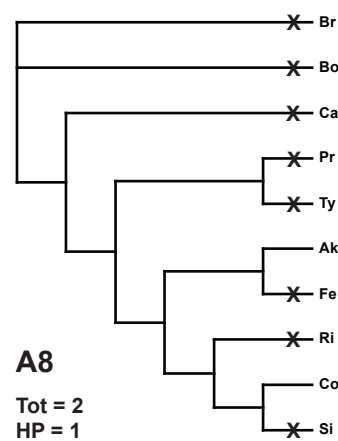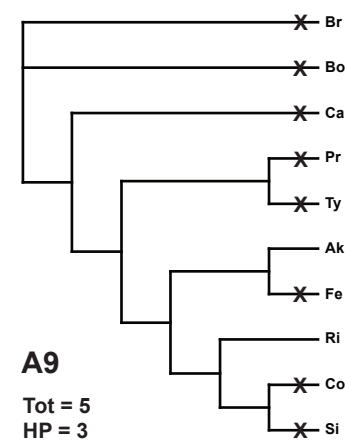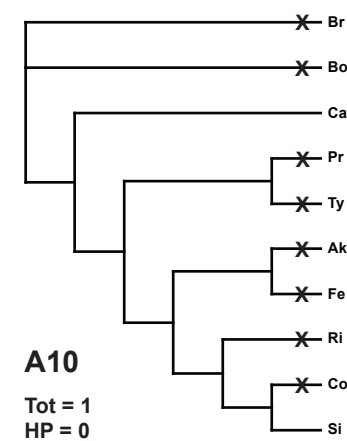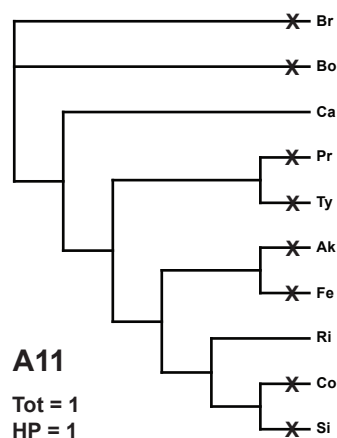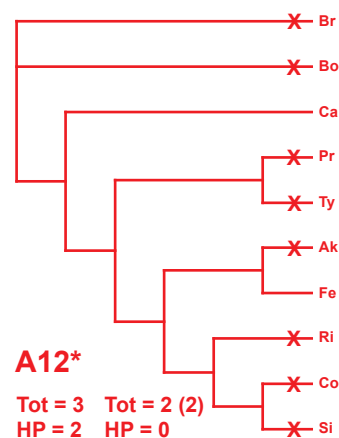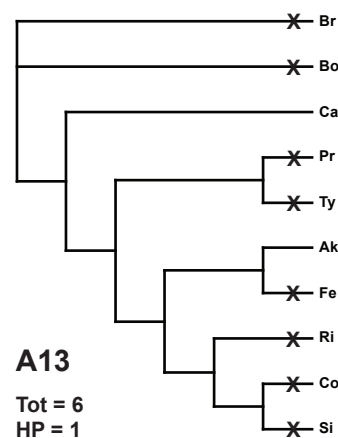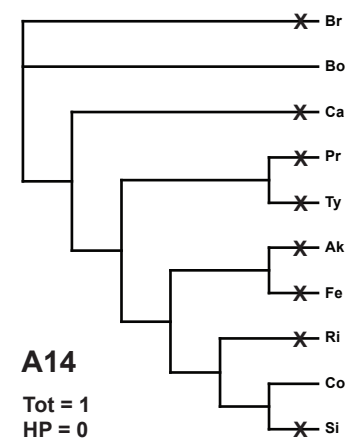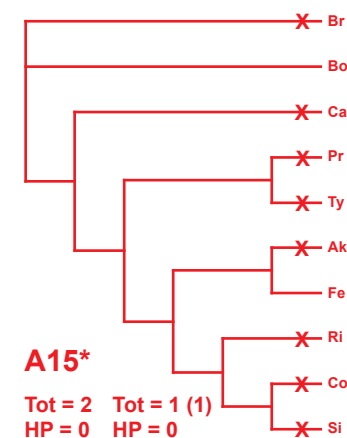

Figure S2

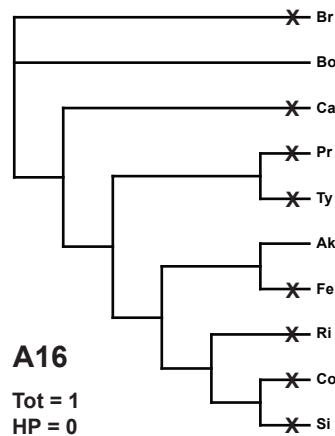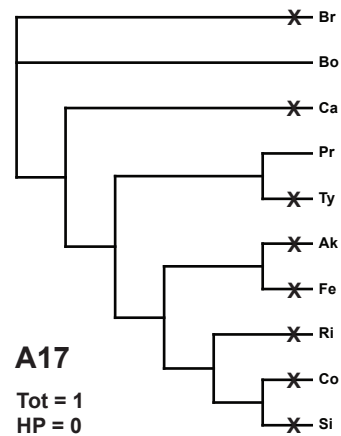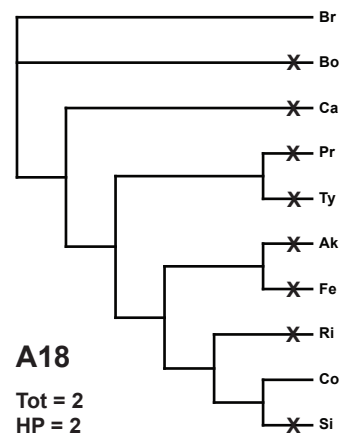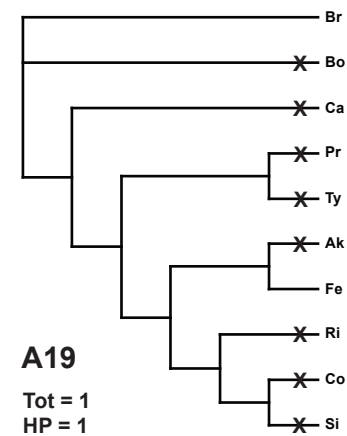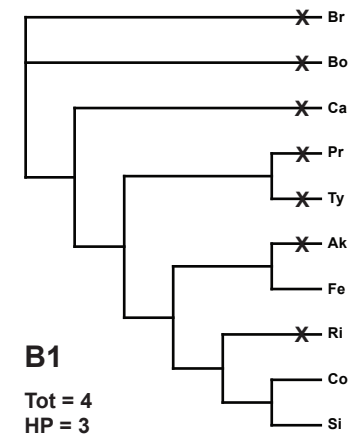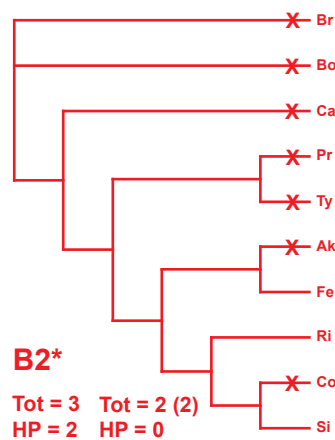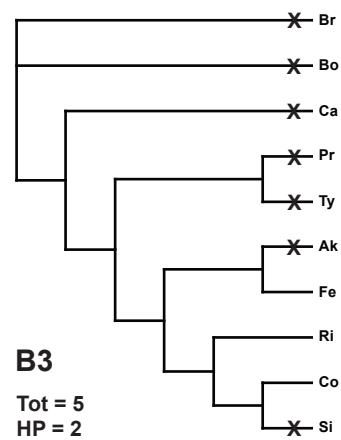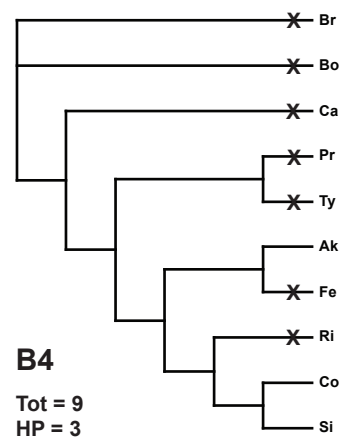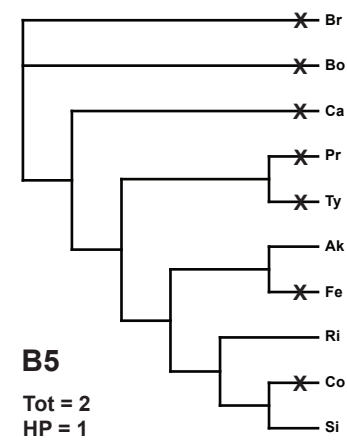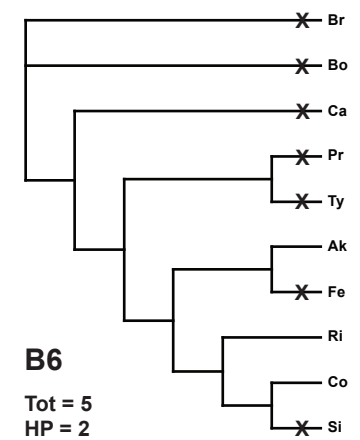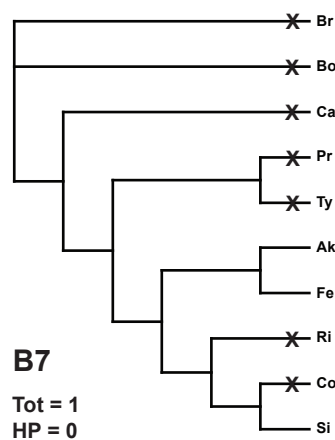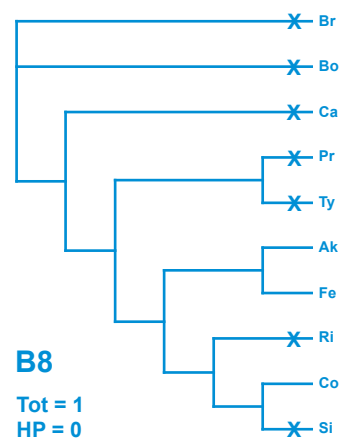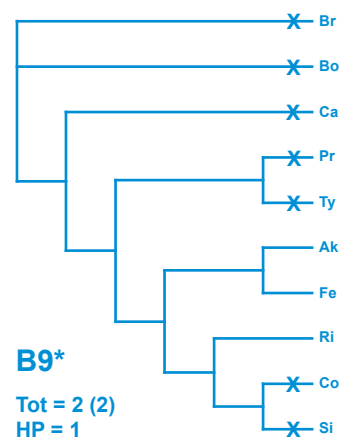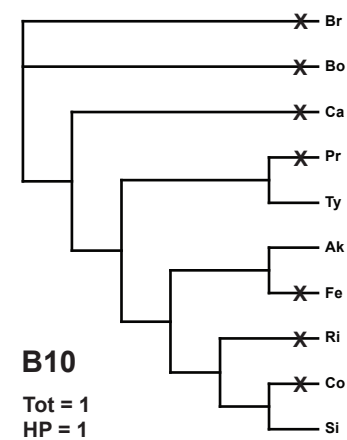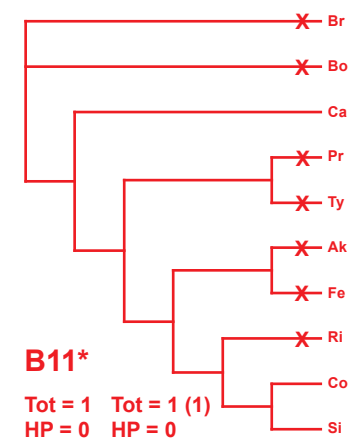

Figure S2

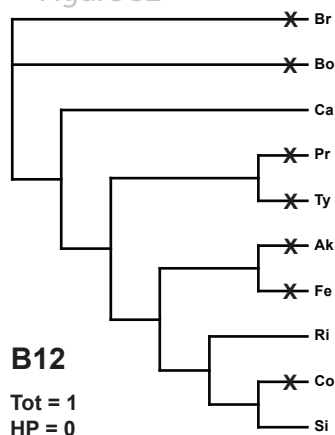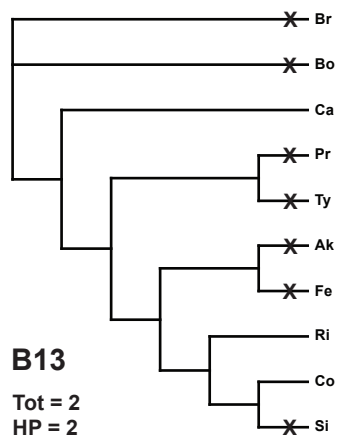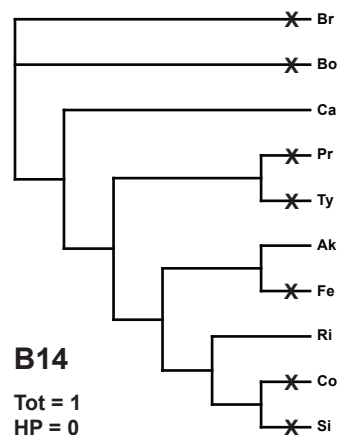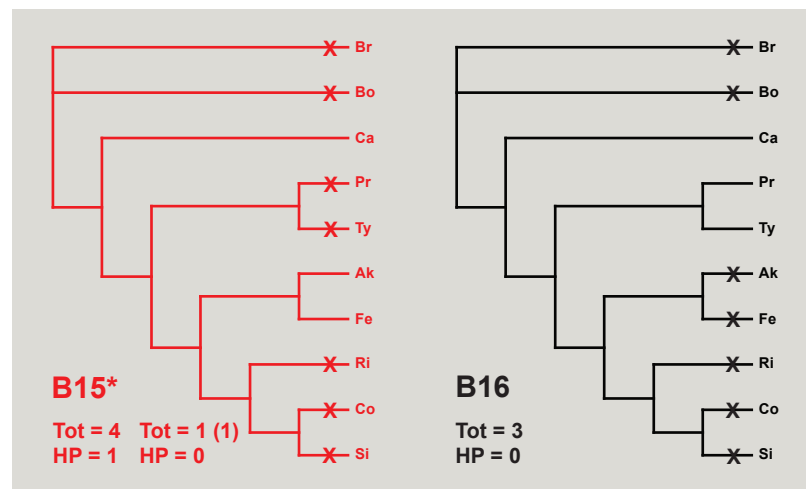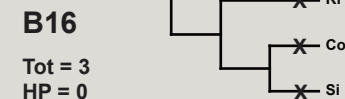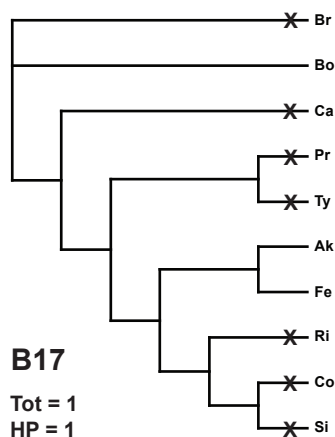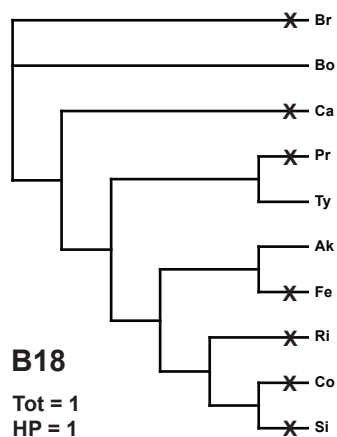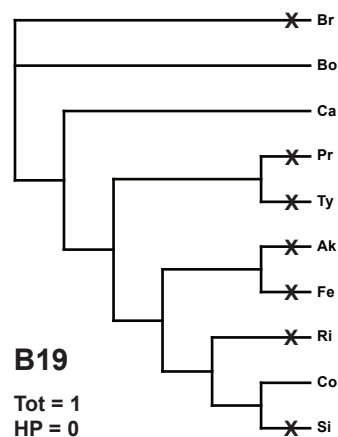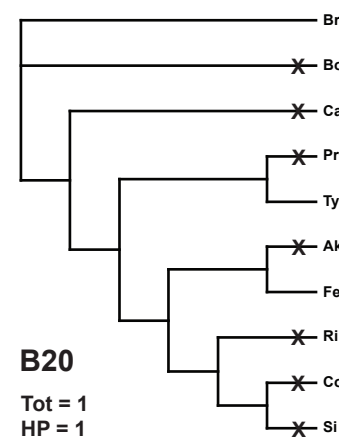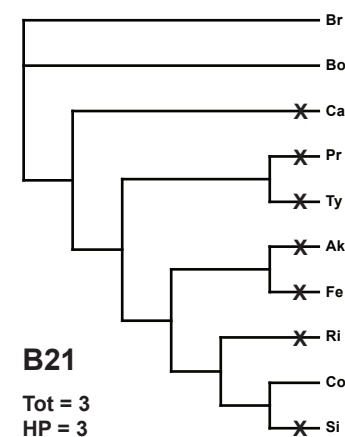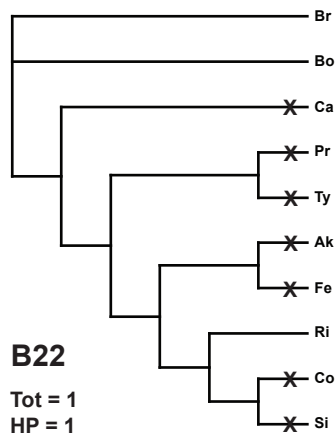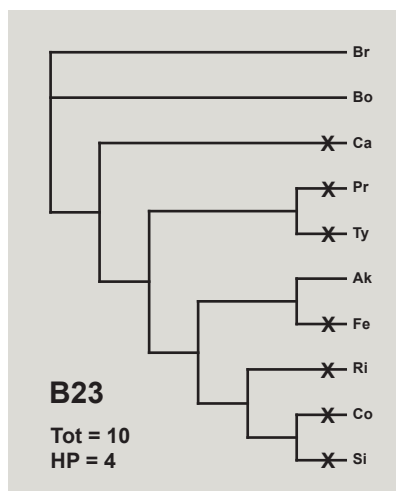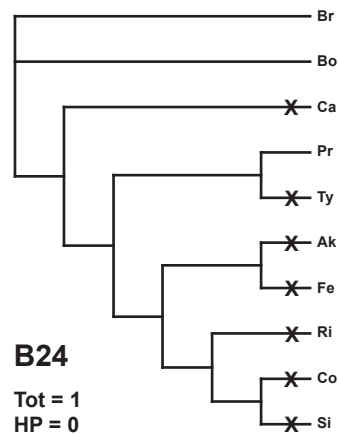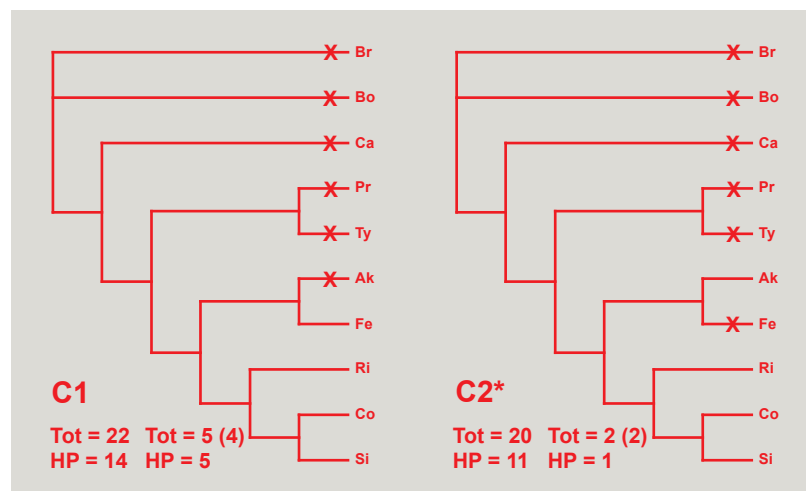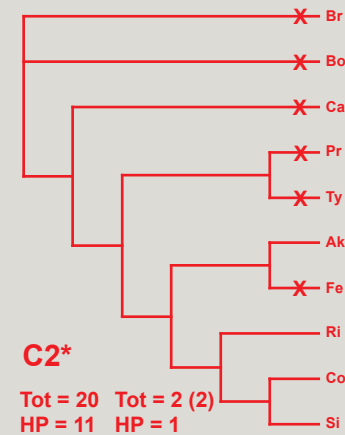

Figure S2

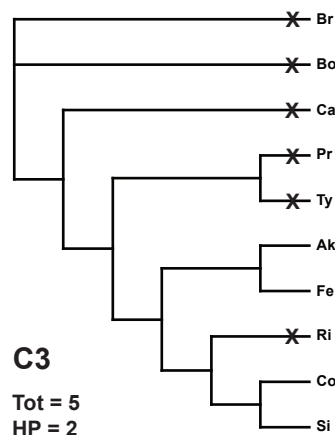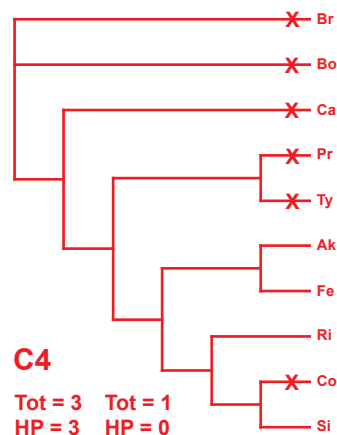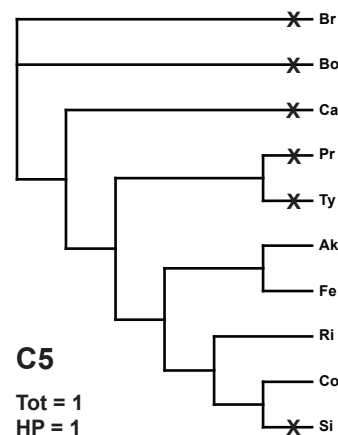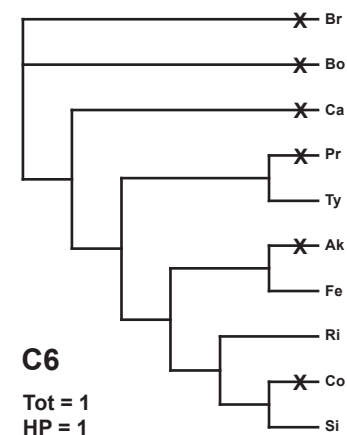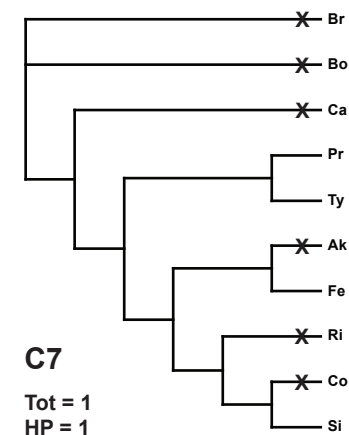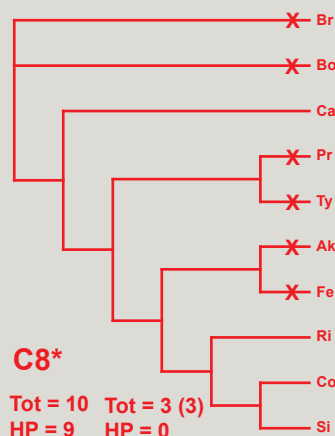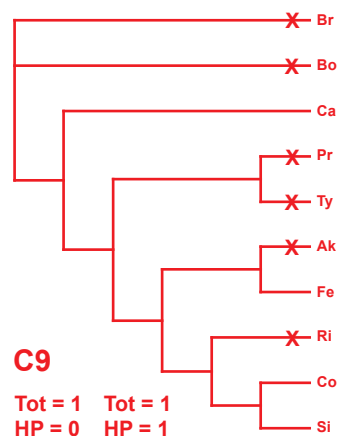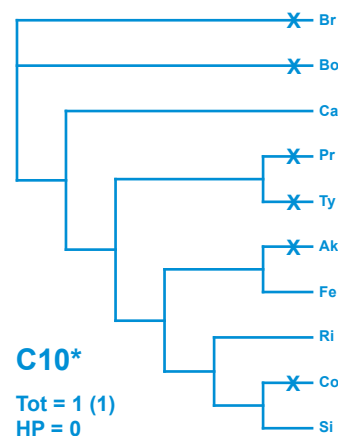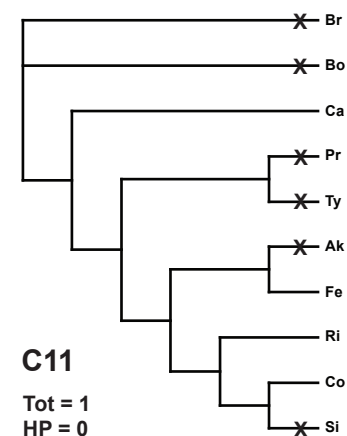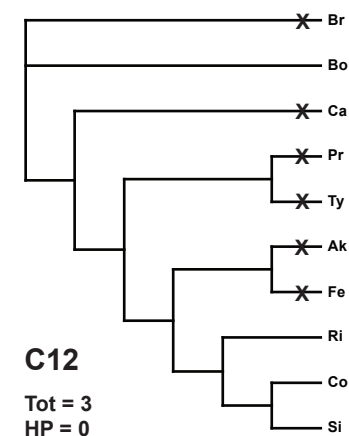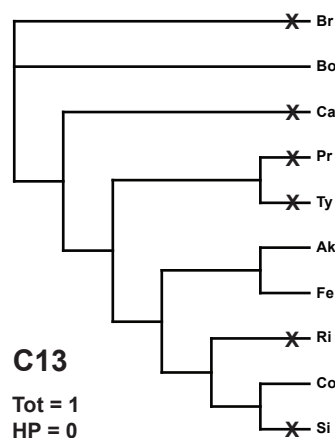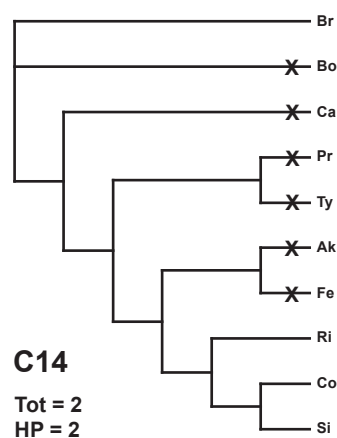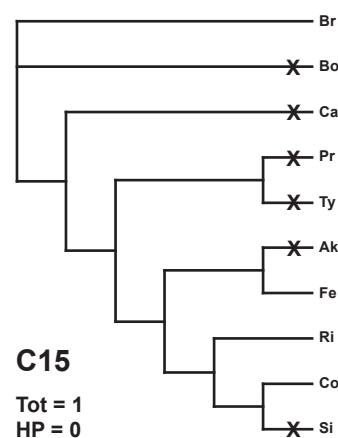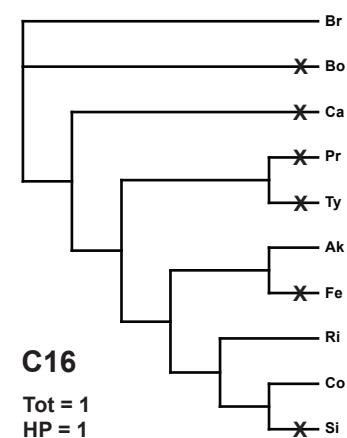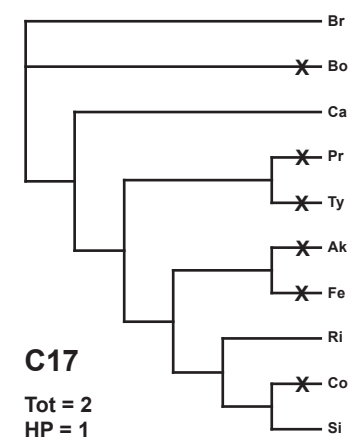

Figure S2

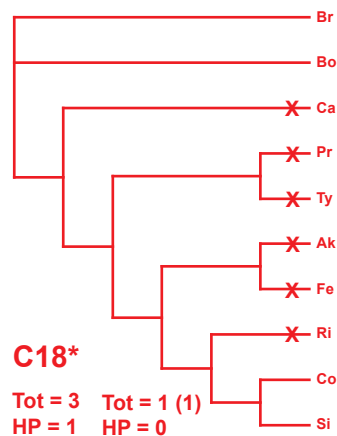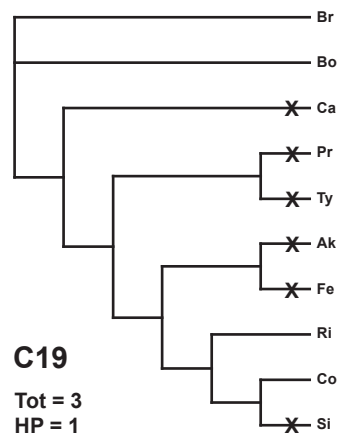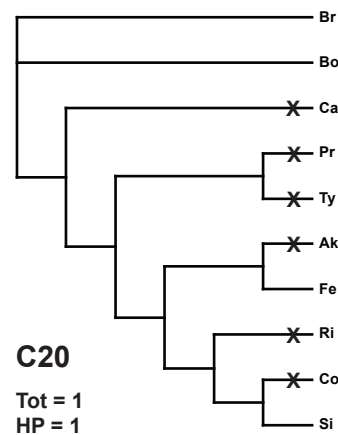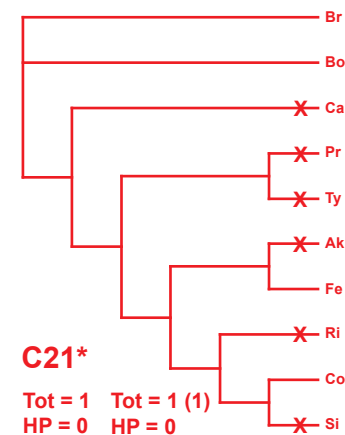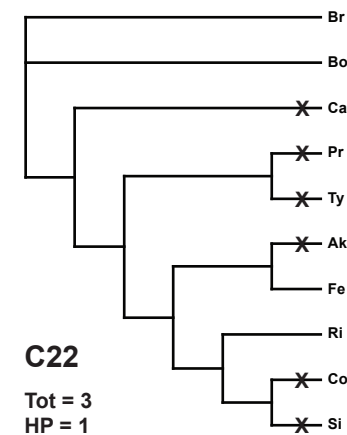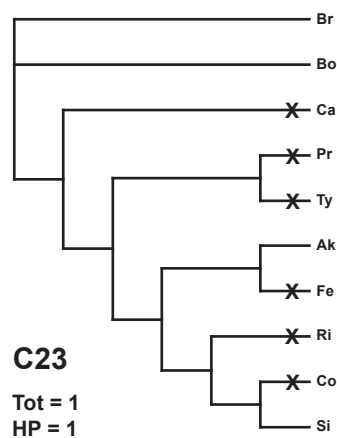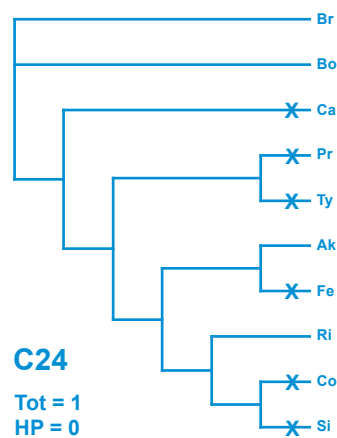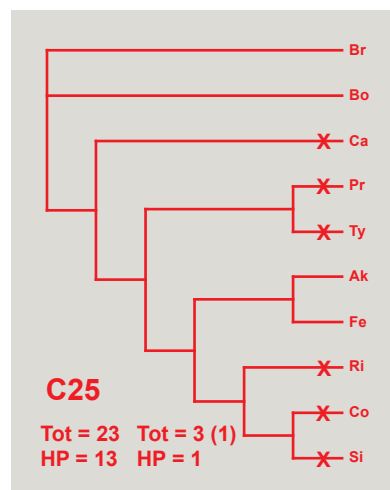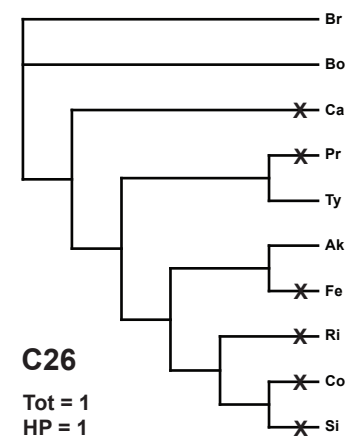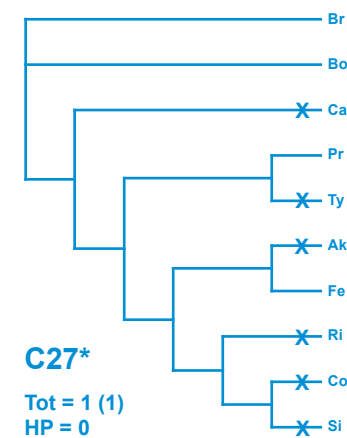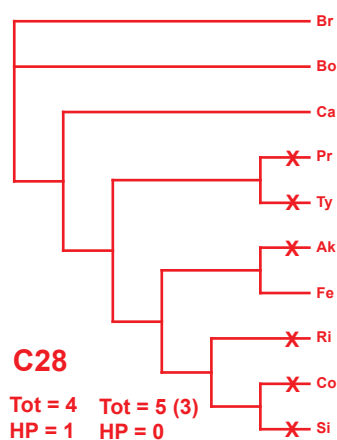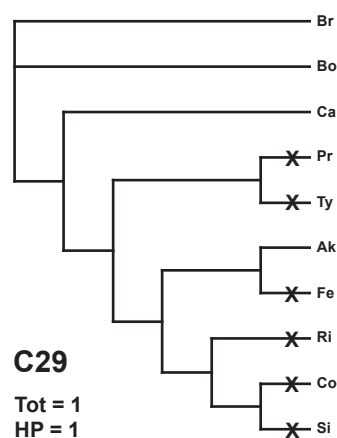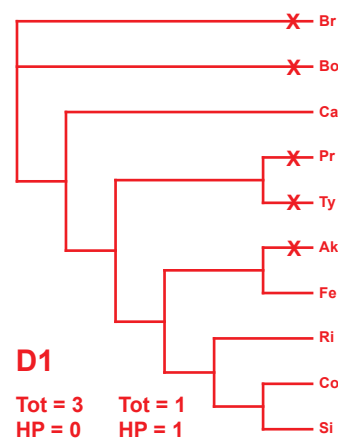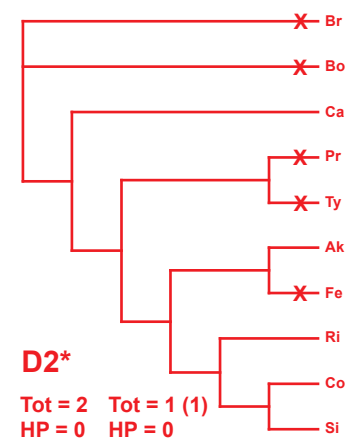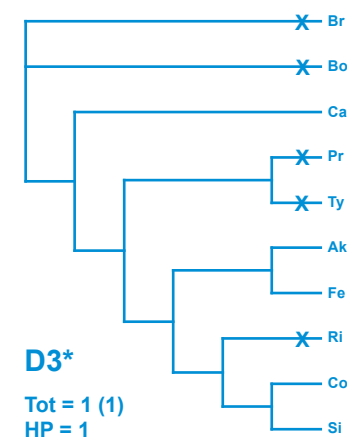

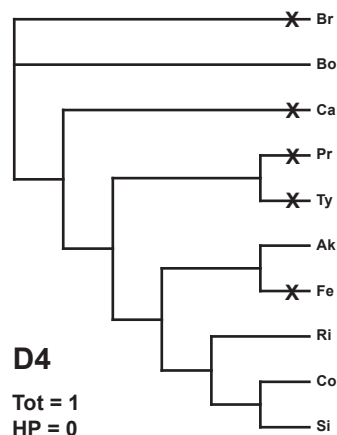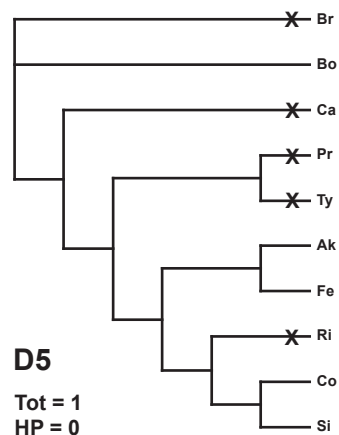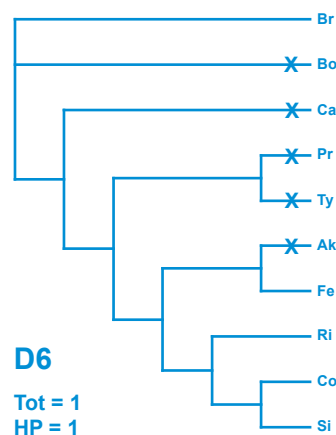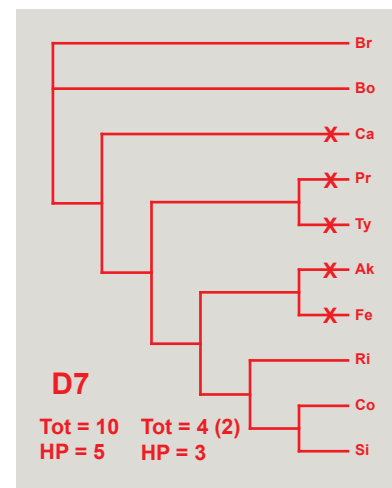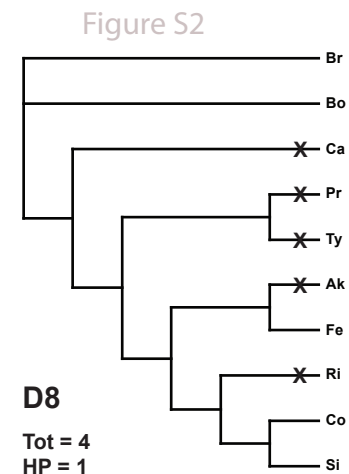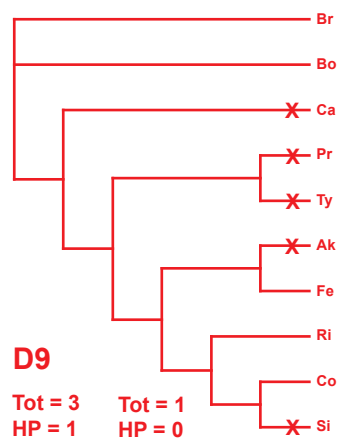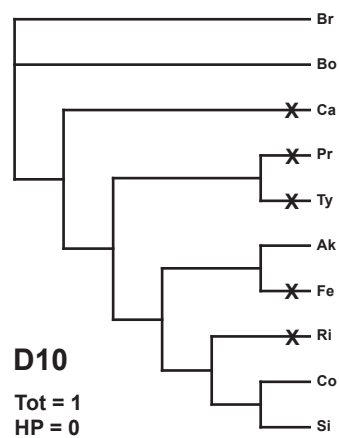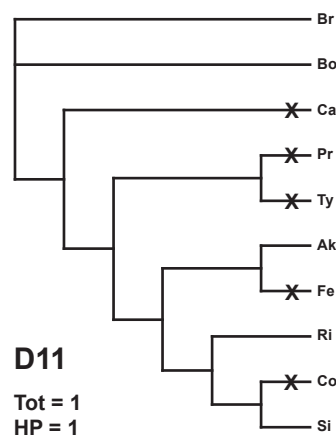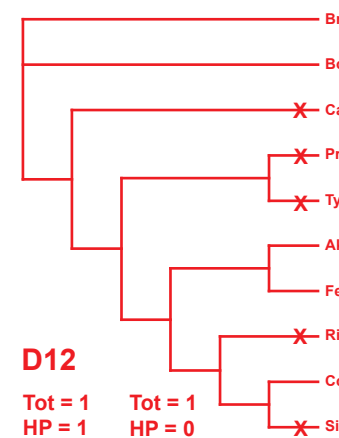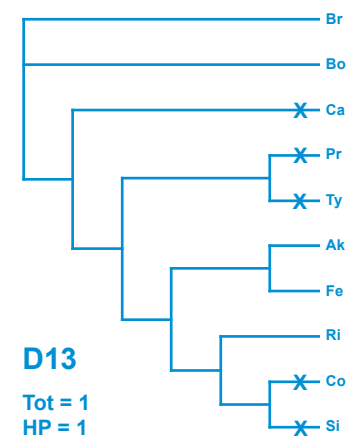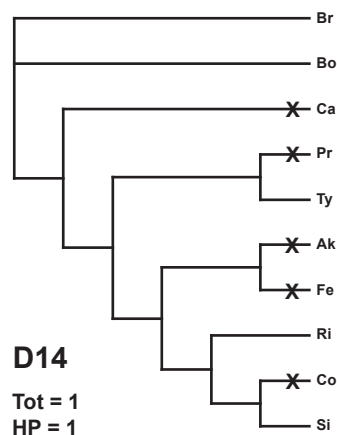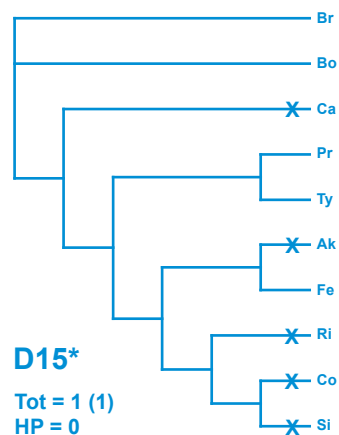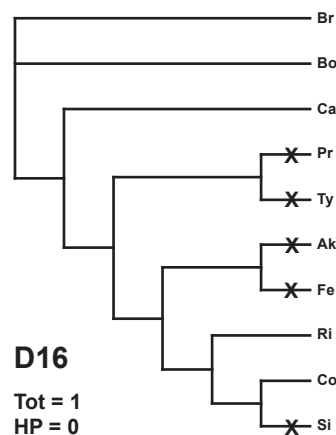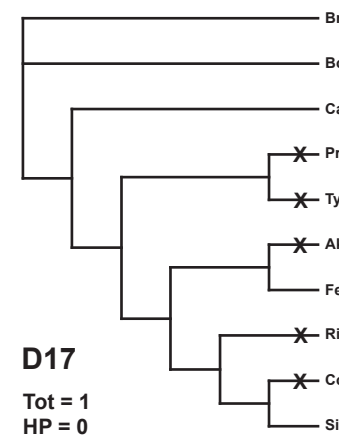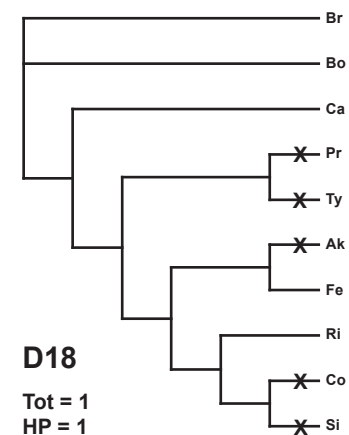

Figure S2

Figure S2

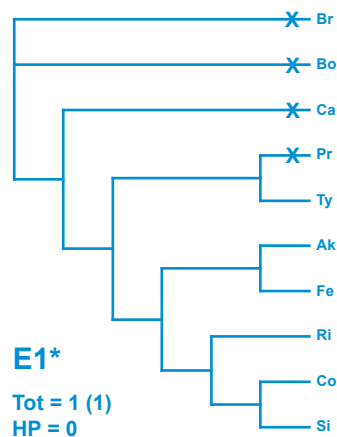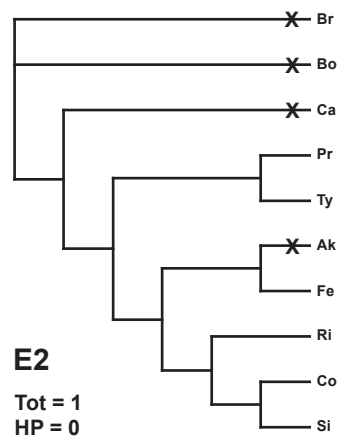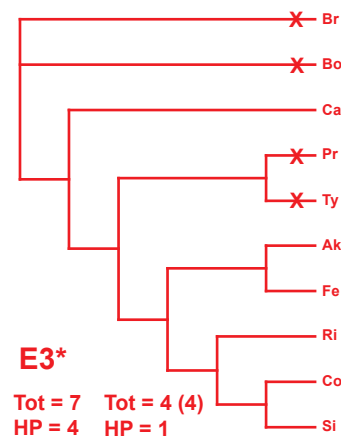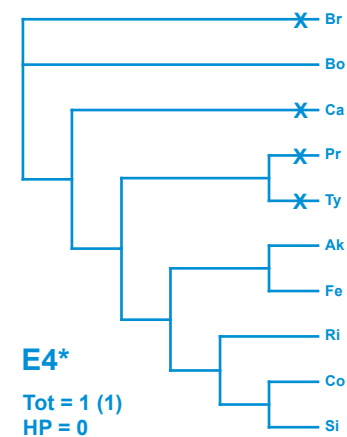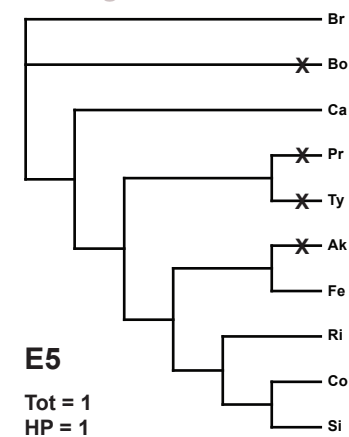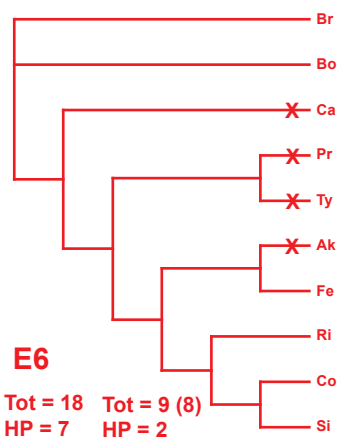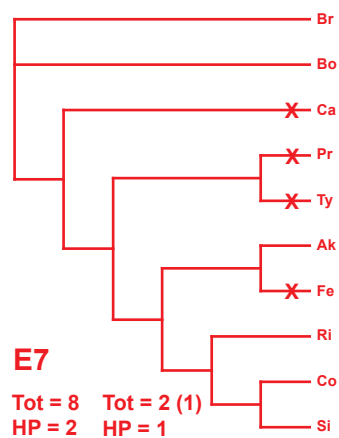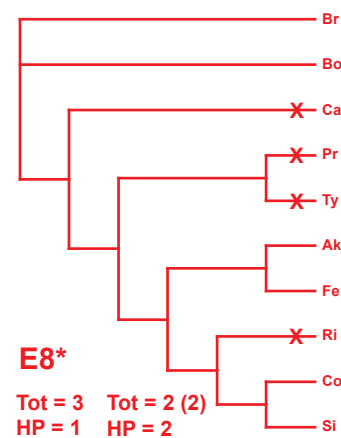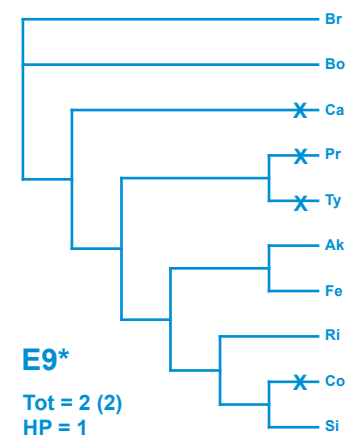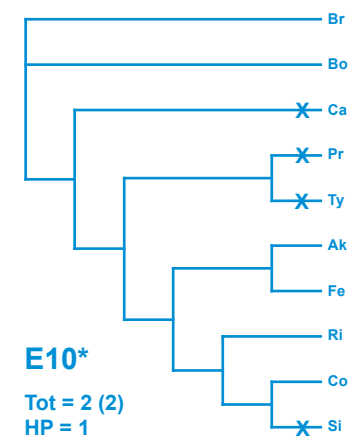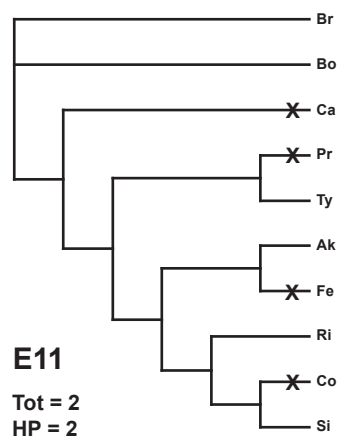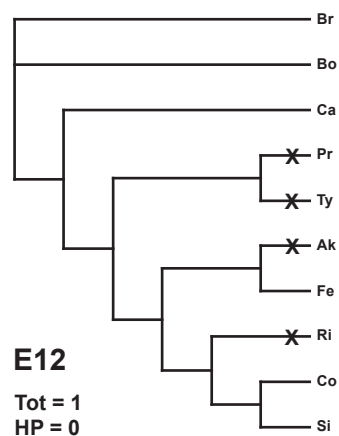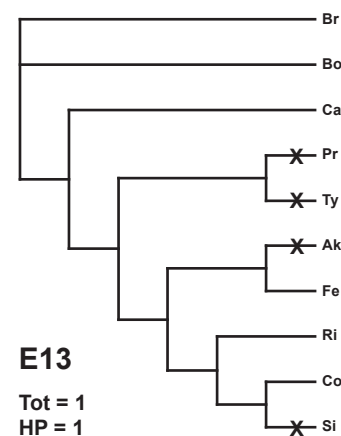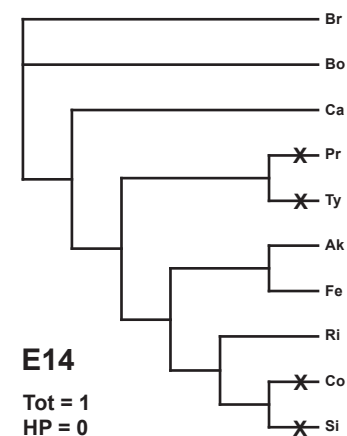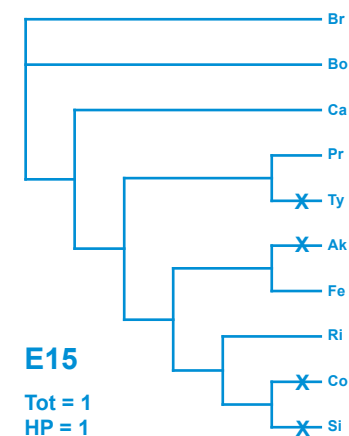

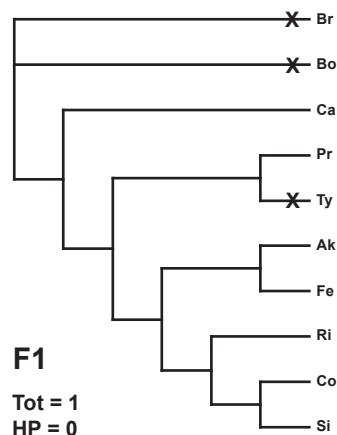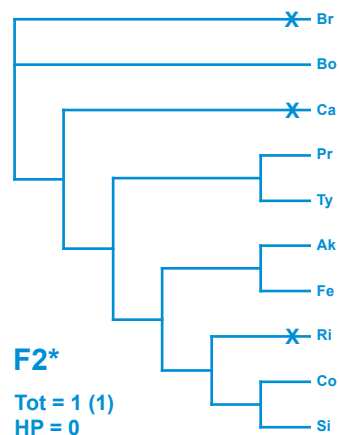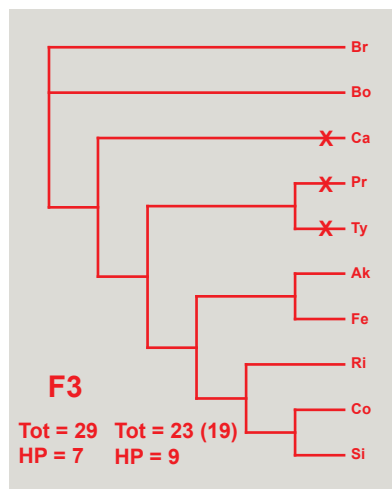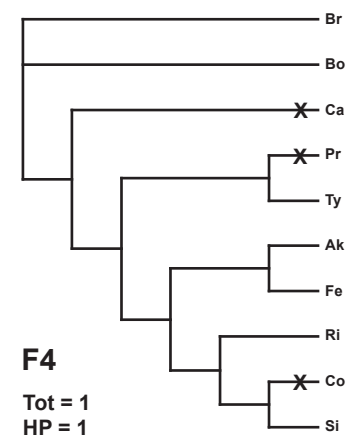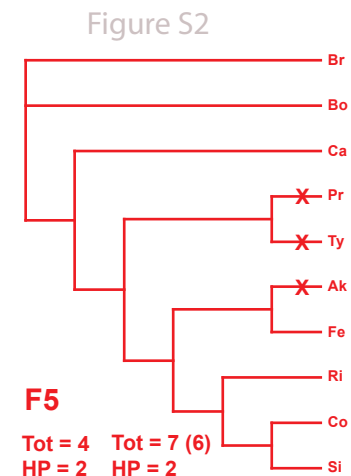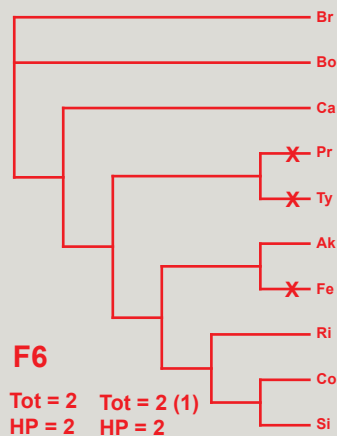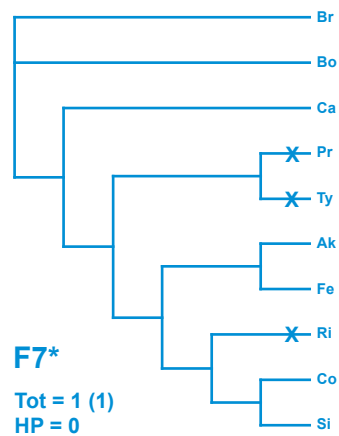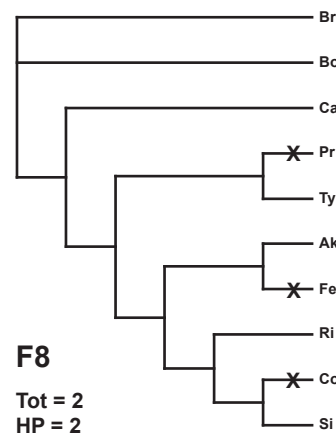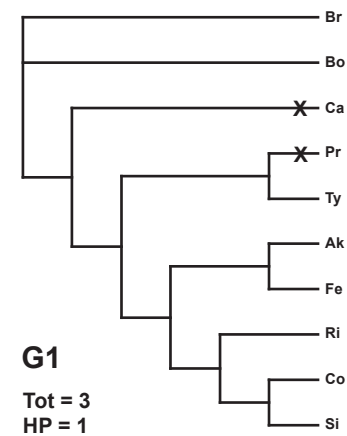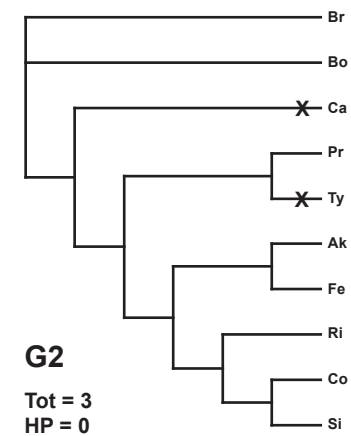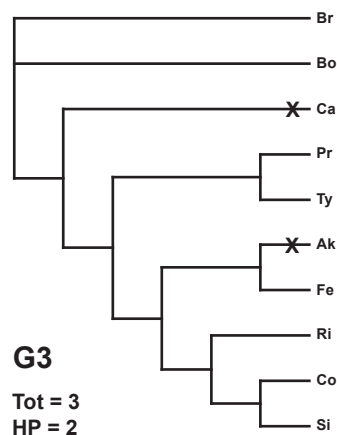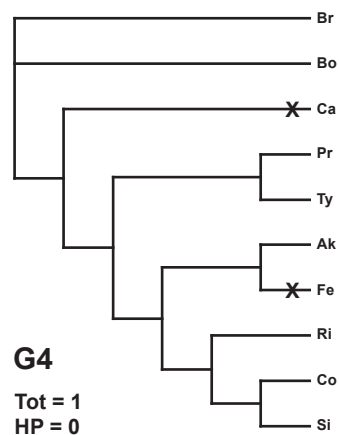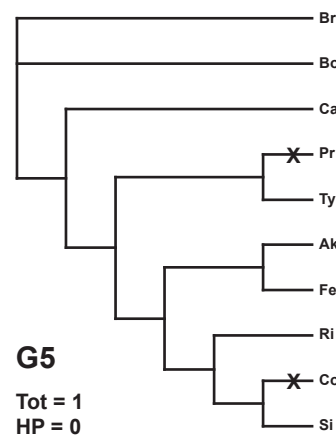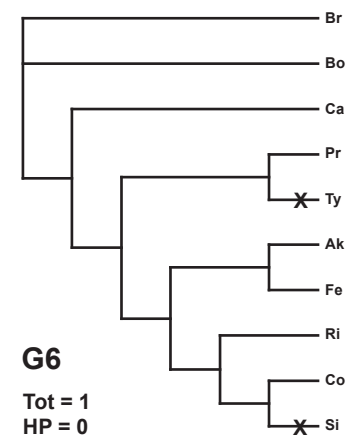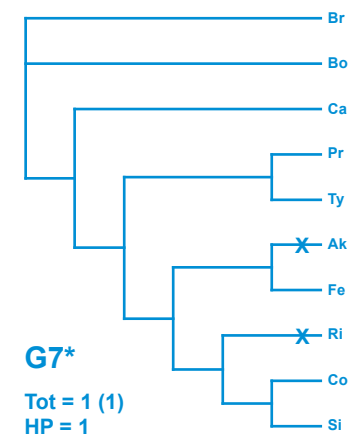

Figure S2

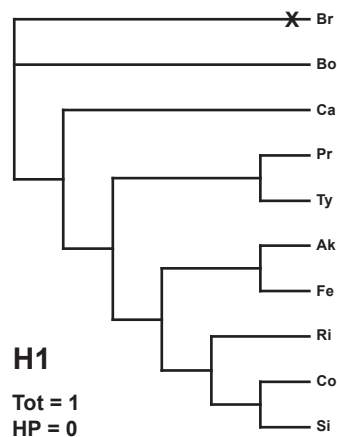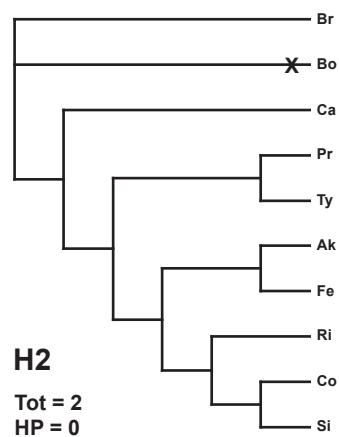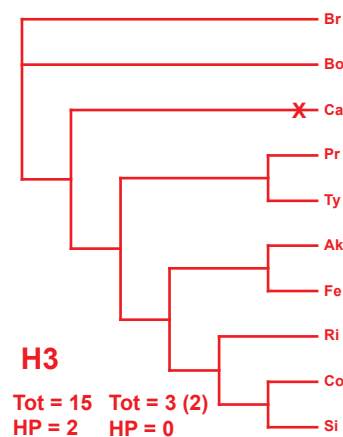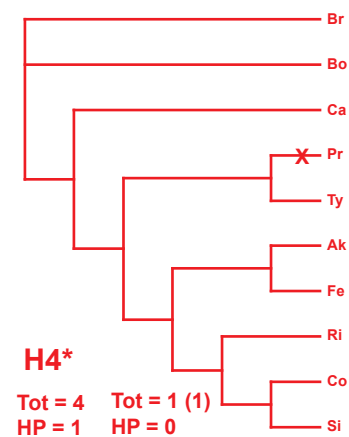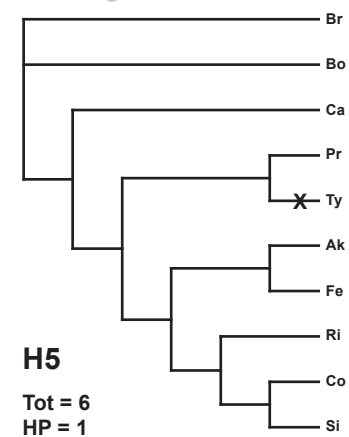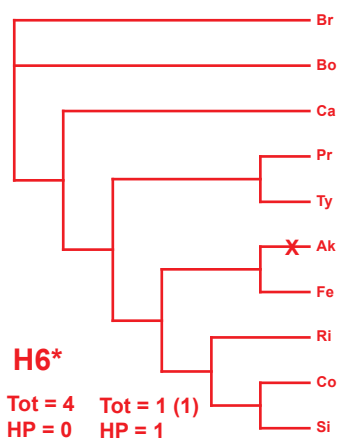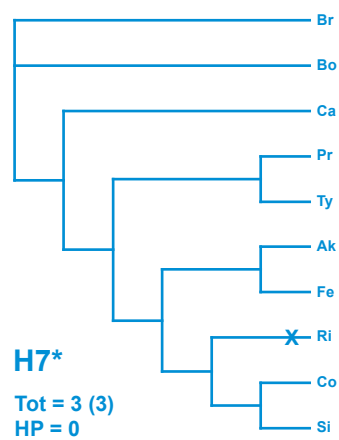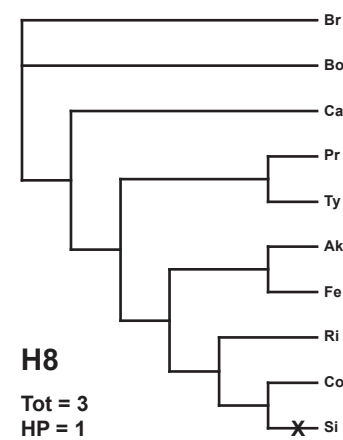

Supplement: Figure S2 — Distribution of 637 representative and non-representative class 2 OGs (C2OGs) over estimated rickettsial phylogeny. These OGs likely include pseudogenes, genes with less conserved functions in rickettsiae, and laterally acquired genes. Black = strictly representative OGs, blue = strictly non-representative OGs, red = both representative and non-representative OGs. Top numbers depict total number of OGs and bottom numbers show proportion of hypothetical proteins. Numbers in parentheses depict the proportion of non-representative OGs made representative via concatenation of split ORFs (see Table S1). Asterisks denote distributions that are made entirely representative after split ORF concatenation (27 of 47 non-representative distributions; see Table 4 and Table S1). (2.99 MB PDF) [file pone.0002018.s002.pdf]

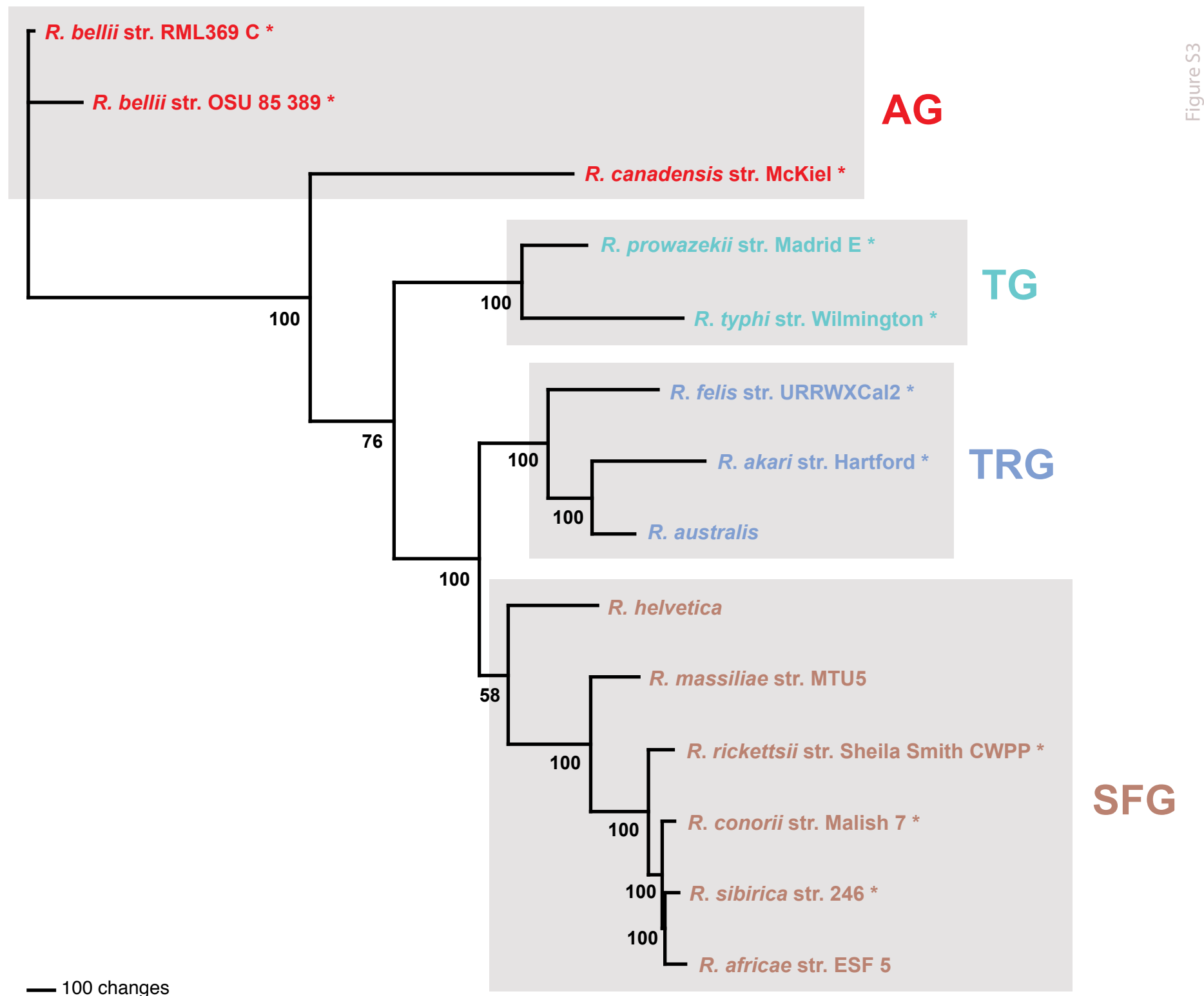

Supplement: Figure S3 — Phylogenetic analysis of 14 rickettsial taxa. Tree estimated using the same 16 proteins as the analysis in Figure 9, with the addition of orthologous sequences from the recently completed genomes of R. massiliae str. MTU5 and R. africae str. ESF 5 (sequences obtained from WGS reads using tBlastn). Tree estimated under parsimony (see text for details). (0.34 MB PDF) [file pone.0002018.s003.pdf]
