# Supplementary material for: Rickettsia Phylogenomics: Unwinding the Intricacies of Obligate Intracellular Life
Source: PLoS One. 2008 Apr 16;3(4):e2018. doi: 10.1371/journal.pone.0002018 (PMC2635572; doi:10.1371/journal.pone.0002018)
Supplement: Table S1 — Phylogenetic analysis of 14 rickettsial taxa. Tree estimated using the same 16 proteins as the analysis in Figure 9, with the addition of orthologous sequences from the recently completed genomes of R. massiliae str. MTU5 and R. africae str. ESF 5 (sequences obtained from WGS reads using tBlastn). Tree estimated under parsimony (see text for details). (0.34 MB PDF) [file pone.0002018.s004.pdf]

Table S1. Distribution and characterization of predicted ORFs within 259 non-representative OGs across ten rickettsial genomes, and the results after manual curation.

No. ORFs	No OGs	RiOG no.	Distribution[1,2]										Designation[3]	Curation[4]
			Br	Bo	Ca	Pr	Ty	Ak	Fe	Ri	Co	Si		
2	49	1498	0	2	0	0	0	0	0	0	0	0	d	non-rep C1OG (FS)
		1499	0	2	0	0	0	0	0	0	0	0	d	non-rep C1OG (FS)
		1500	0	2	0	0	0	0	0	0	0	0	d	non-rep C1OG (FS)
		1501	0	2	0	0	0	0	0	0	0	0	d	non-rep C1OG (FS)
		1503	0	0	0	0	2	0	0	0	0	0	d	non-rep C1OG (FS)
		1504	0	0	0	0	0	0	0	0	0	2	d	non-rep C1OG (FS)
		1560	0	0	0	0	0	0	2	0	0	0	d	non-rep C1OG (FS)
		1564	0	0	0	0	0	0	2	0	0	0	d	non-rep C1OG (FS)
		1568	0	0	0	0	0	0	2	0	0	0	d	non-rep C1OG (FS)
		1569	0	0	0	0	0	0	2	0	0	0	d	non-rep C1OG (FS)
		1573	0	0	0	0	0	0	2	0	0	0	d	non-rep C1OG (FS)
		1574	0	0	0	0	0	0	2	0	0	0	d	non-rep C1OG (FS)
		1575	0	0	0	0	0	0	2	0	0	0	d	non-rep C1OG (FS)
		1577	0	0	0	0	0	0	2	0	0	0	d	non-rep C1OG (FS)
		**1578**	**0**	**0**	**0**	**0**	**0**	**0**	**2**	**0**	**0**	**0**	**1 (pRF), no pRFδ**	**rep C1OG (S)**
		**1579**	**0**	**0**	**0**	**0**	**0**	**0**	**2**	**0**	**0**	**0**	**1 (pRF), no pRFδ**	**rep C1OG (S)**
		**1580**	**0**	**0**	**0**	**0**	**0**	**0**	**2**	**0**	**0**	**0**	**1 (pRF), no pRFδ**	**rep C1OG (S)**
		**1581**	**0**	**0**	**0**	**0**	**0**	**0**	**2**	**0**	**0**	**0**	**1 (pRF), no pRFδ**	**rep C1OG (S)**
		**1582**	**0**	**0**	**0**	**0**	**0**	**0**	**2**	**0**	**0**	**0**	**1 (pRF), no pRFδ**	**rep C1OG (S)**
		**1583**	**0**	**0**	**0**	**0**	**0**	**0**	**2**	**0**	**0**	**0**	**1 (pRF), no pRFδ**	**rep C1OG (S)**
		**1584**	**0**	**0**	**0**	**0**	**0**	**0**	**2**	**0**	**0**	**0**	**1 (pRF), no pRFδ**	**rep C1OG (S)**
		**1585**	**0**	**0**	**0**	**0**	**0**	**0**	**2**	**0**	**0**	**0**	**1 (pRF), no pRFδ**	**rep C1OG (S)**
		**1586**	**0**	**0**	**0**	**0**	**0**	**0**	**2**	**0**	**0**	**0**	**1 (pRF), no pRFδ**	**rep C1OG (S)**
		**1587**	**0**	**0**	**0**	**0**	**0**	**0**	**2**	**0**	**0**	**0**	**1 (pRF), no pRFδ**	**rep C1OG (S)**
		**1588**	**0**	**0**	**0**	**0**	**0**	**0**	**2**	**0**	**0**	**0**	**1 (pRF), no pRFδ**	**rep C1OG (S)**
		**1589**	**0**	**0**	**0**	**0**	**0**	**0**	**2**	**0**	**0**	**0**	**1 (pRF), no pRFδ**	**rep C1OG (S)**
		**1590**	**0**	**0**	**0**	**0**	**0**	**0**	**2**	**0**	**0**	**0**	**1 (pRF), no pRFδ**	**rep C1OG (S)**
		**1591**	**0**	**0**	**0**	**0**	**0**	**0**	**2**	**0**	**0**	**0**	**1 (pRF), no pRFδ**	**rep C1OG (S)**
		**1592**	**0**	**0**	**0**	**0**	**0**	**0**	**2**	**0**	**0**	**0**	**1 (pRF), no pRFδ**	**rep C1OG (S)**

		1593	0	0	0	0	0	0	2	0	0	0	1 (pRF), no pRFδ	rep C1OG (S)
		1594	0	0	0	0	0	0	2	0	0	0	1 (pRF), no pRFδ	rep C1OG (S)
		1595	0	0	0	0	0	0	2	0	0	0	1 (pRF), no pRFδ	rep C1OG (S)
		1596	0	0	0	0	0	0	2	0	0	0	1 (pRF), no pRFδ	rep C1OG (S)
		1597	0	0	0	0	0	0	2	0	0	0	1 (pRF), no pRFδ	rep C1OG (S)
		1598	0	0	0	0	0	0	2	0	0	0	1 (pRF), no pRFδ	rep C1OG (S)
		1599	0	0	0	0	0	0	2	0	0	0	1 (pRF), no pRFδ	rep C1OG (S)
		1600	0	0	0	0	0	0	2	0	0	0	1 (pRF), no pRFδ	rep C1OG (S)
		1601	0	0	0	0	0	0	2	0	0	0	1 (pRF), no pRFδ	rep C1OG (S)
		1602	0	0	0	0	0	0	2	0	0	0	1 (pRF), no pRFδ	rep C1OG (S)
		1603	0	0	0	0	0	0	2	0	0	0	1 (pRF), no pRFδ	rep C1OG (S)
		1604	0	0	0	0	0	0	2	0	0	0	1 (pRF), no pRFδ	rep C1OG (S)
		1605	0	0	0	0	0	0	2	0	0	0	1 (pRF), no pRFδ	rep C1OG (S)
		1606	0	0	0	0	0	0	2	0	0	0	1 (pRF), no pRFδ	rep C1OG (S)
		1607	0	0	0	0	0	0	2	0	0	0	1 (pRF), no pRFδ	rep C1OG (S)
		1608	0	0	0	0	0	0	2	0	0	0	1 (pRF), no pRFδ	rep C1OG (S)
		1609	0	0	0	0	0	0	2	0	0	0	1 (pRF), no pRFδ	rep C1OG (S)
		1610	0	0	0	0	0	0	2	0	0	0	1 (pRF), no pRFδ	rep C1OG (S)
		1740	2	0	0	0	0	0	0	0	0	0	d	non-rep C1OG (FS)
		2073	0	0	0	0	0	2	0	0	0	0	d	non-rep C1OG (FS)
3	14	1276	0	0	0	0	0	0	3	0	0	0	d(2)	non-rep C1OG (FS)
		1277	0	0	0	0	0	0	3	0	0	0	d(2)	non-rep C1OG (FS)
		1278	0	1	0	0	0	0	2	0	0	0	1 (pRF), no pRFδ	rep C2OG; A15
		1279	0	0	0	0	0	0	3	0	0	0	d (pRF), no pRFδ	non-rep C1OG (FS)
		1280	0	0	0	0	0	0	3	0	0	0	2 (Fe & pRF), no pRFδ	non-rep C1OG (FS)
		1281	0	0	0	0	0	0	3	0	0	0	2 (Fe & pRF), no pRFδ	non-rep C1OG (FS)
		1282	0	0	0	0	0	0	3	0	0	0	d (pRF), no pRFδ	non-rep C1OG (FS)
		1283	0	0	0	0	0	0	3	0	0	0	d (pRF), no pRFδ	non-rep C1OG (FS)
		1401	0	0	1	0	0	0	2	0	0	0	1 (pRF), no pRFδ	rep C2OG; A12
		1402	0	0	1	0	0	0	2	0	0	0	1 (pRF), no pRFδ	rep C2OG; A12
		1409	1	2	0	0	0	0	0	0	0	0	s	rep C1OG; bellii
		1419	1	2	0	0	0	0	0	0	0	0	s	rep C1OG; bellii
		1440	2	1	0	0	0	0	0	0	0	0	d	non-rep C1OG; bellii
		1483	0	0	0	0	0	1	2	0	0	0	d	non-rep C1OG; TRG

4	12	1156	0	0	0	0	0	0	0	1	2	1	s	rep C1OG; SFG
		1157	0	0	0	0	0	0	0	1	2	1	t; s; t	rep C1OG; SFG
		1163	0	0	0	0	0	0	0	1	2	1	s	rep C1OG; SFG
		1167	0	0	0	0	0	0	0	2	1	1	s	rep C1OG; SFG
		1173	0	0	0	0	0	0	0	1	1	2	t; s	rep C1OG; SFG
		1184	0	0	2	0	0	0	0	0	1	1	s	rep C2OG; B11
		1204	1	1	0	0	0	0	2	0	0	0	d	non-rep C1OG; plasmid
		1209	1	2	0	0	0	0	1	0	0	0	s	rep C1OG; plasmid
		1210	1	2	0	0	0	0	1	0	0	0	d	non-rep C1OG; plasmid
		1223	0	0	1	0	0	1	2	0	0	0	1 (pRF), no pRFδ	rep C2OG; B15
		1234	0	0	0	0	0	1	1	0	2	0	d	non-rep C2OG; B8
		1264	0	0	0	0	0	2	1	1	0	0	s	rep C2OG; B9
5	28	1055	0	0	0	0	0	0	1	2	0	2	s; s	rep C2OG; B2
		1056	0	0	0	0	0	0	1	2	0	2	s; s	rep C2OG; B2
		1057	0	0	0	0	0	0	5	0	0	0	2 (pRF; 1 s), no pRFδ (2)	non-rep C1OG (FS)
		1058	0	0	0	0	0	0	0	1	2	2	s; s	rep C1OG; SFG
		1059	0	0	0	0	0	0	1	1	2	1	t; s	rep C2OG; C1
		1060	0	0	0	0	0	0	2	1	1	1	s; t	rep C2OG; C1
		1061	0	0	0	0	0	0	1	1	1	2	t; s	rep C2OG; C1
		1062	0	0	0	0	0	0	0	2	2	1	d; d	non-rep C1OG; SFG
		1064	0	0	2	0	0	0	1	1	0	1	s	rep C2OG; C10
		1065	0	0	2	0	0	0	0	1	1	1	s; t	rep C2OG; C8
		1066	0	0	2	0	0	0	0	1	1	1	s	rep C2OG; C8
		1067	0	0	1	0	0	0	0	1	1	2	t; s	rep C2OG; C8
		1068	0	0	1	0	0	0	2	0	1	1	d	non-rep C2OG; C9
		1073	1	1	1	0	0	0	2	0	0	0	t; 1 (pRF), no pRFδ	rep C2OG; C28
		1081	2	1	0	0	0	0	0	0	1	1	s	rep C2OG; C18
		1085	1	1	1	0	0	0	2	0	0	0	t; 1 (pRF), no pRFδ	rep C2OG; C28
		1086	1	1	1	0	0	0	2	0	0	0	t; 1 (pRF), no pRFδ	rep C2OG; C28
		1088	1	1	0	0	0	0	2	0	1	0	s; t	rep C2OG; C21
		1091	3	2	0	0	0	0	0	0	0	0	d(2); d	non-rep C1OG; bellii
		1092	1	1	1	0	0	0	2	0	0	0	d	non-rep C2OG; C28
		1098	2	2	0	0	0	0	1	0	0	0	d; d	non-rep C1OG; bellii
		1100	1	1	0	0	0	1	2	0	0	0	t; d	non-rep C2OG; C25
		1108	1	1	0	0	0	1	0	2	0	0	d	non-rep C2OG; C22
		1120	0	0	0	0	0	2	1	2	0	0	s; s	rep C2OG; B9

		1125	0	0	0	0	0	1	4	0	0	0	d(3)	non-rep C1OG; TRG
		**1128**	**1**	**2**	**0**	**0**	**0**	**1**	**1**	**0**	**0**	**0**	**s**	**rep C2OG; C25**
		**1132**	**0**	**0**	**0**	**0**	**0**	**1**	**0**	**2**	**1**	**1**	**s**	**rep C2OG; C2**
		1134	0	0	0	0	0	2	1	1	0	1	d	non-rep C2OG; C4
6	16	1013	1	2	0	0	0	0	0	1	1	1	d	non-rep C2OG; D7
		1014	1	1	0	0	0	0	2	1	1	0	d	non-rep C2OG; D9
		1022	2	1	1	0	0	0	2	0	0	0	d; d	non-rep C2OG; C28
		1024	0	0	0	0	0	1	1	2	1	1	d	non-rep C1OG; SFG
		**1029**	**0**	**0**	**0**	**0**	**0**	**1**	**2**	**1**	**1**	**1**	**s; t**	**rep C1OG; TRG+SFG**
		**1030**	**0**	**0**	**0**	**0**	**0**	**1**	**1**	**2**	**1**	**1**	**t; s**	**rep C1OG; TRG+SFG**
		**1042**	**0**	**0**	**0**	**0**	**0**	**2**	**1**	**1**	**1**	**1**	**s; t**	**rep C1OG; TRG+SFG**
		**1044**	**0**	**0**	**0**	**0**	**0**	**1**	**2**	**1**	**1**	**1**	**t; s**	**rep C1OG; TRG+SFG**
		**1049**	**0**	**0**	**0**	**0**	**0**	**1**	**0**	**2**	**1**	**2**	**s; s**	**rep C2OG; C2**
		**1051**	**0**	**0**	**0**	**0**	**0**	**1**	**2**	**1**	**1**	**1**	**s**	**rep C1OG; TRG+SFG**
		**1053**	**1**	**1**	**0**	**2**	**0**	**0**	**2**	**0**	**0**	**0**	**s; s**	**rep C2OG; C27**
		**1054**	**1**	**1**	**0**	**1**	**1**	**0**	**2**	**0**	**0**	**0**	**t; 1 (pRF), no pRFδ**	**rep C2OG; D15**
		993	0	0	0	0	0	0	6	0	0	0	d(5)	non-rep C1OG (FS)
		994	0	0	0	0	0	0	6	0	0	0	d(5)	non-rep C1OG (FS)
		995	0	0	0	0	0	0	6	0	0	0	d(5)	non-rep C1OG (FS)
		**996**	**0**	**0**	**0**	**0**	**0**	**0**	**1**	**2**	**1**	**2**	**s; s**	**rep C2OG; C1**
7	23	928	0	0	0	0	0	0	7	0	0	0	5 (3 Fe & 2 pRF), no pRFδ (2)	non-rep C1OG (FS)
		929	0	0	0	0	0	0	1	2	2	2	d; d; d	non-rep C2OG; C1
		**930**	**1**	**1**	**0**	**0**	**0**	**0**	**1**	**2**	**1**	**1**	**s**	**rep C2OG; E6**
		932	2	1	0	0	0	0	0	2	1	1	d; s	non-rep C2OG; D7
		933	1	0	0	0	0	0	2	1	1	2	d; d	non-rep C2OG; D6
		935	6	1	0	0	0	0	0	0	0	0	d(5)	non-rep C1OG; bellii
		**936**	**1**	**1**	**0**	**0**	**0**	**0**	**2**	**1**	**1**	**1**	**1 (pRF), no pRFδ**	**rep C2OG; E6**
		937	6	1	0	0	0	0	0	0	0	0	d(5)	non-rep C1OG; bellii
		**939**	**1**	**2**	**0**	**0**	**0**	**0**	**1**	**1**	**1**	**1**	**s**	**rep C2OG; E6**
		**940**	**2**	**2**	**0**	**0**	**0**	**0**	**0**	**1**	**1**	**1**	**s; s**	**rep C2OG; D7**
		942	6	1	0	0	0	0	0	0	0	0	d(5)	non-rep C1OG; bellii
		**953**	**0**	**0**	**1**	**0**	**0**	**1**	**1**	**0**	**2**	**2**	**s; s**	**rep C2OG; D3**
		954	2	1	0	0	0	1	0	1	1	1	d	non-rep C2OG; E7
		**957**	**0**	**0**	**0**	**0**	**0**	**1**	**1**	**1**	**2**	**2**	**s; s**	**rep C1OG; TRG+SFG**
		**959**	**0**	**1**	**0**	**0**	**0**	**2**	**1**	**1**	**1**	**1**	**s**	**rep C2OG; E4**

		963	0	0	1	0	0	1	1	1	1	2	s	rep C2OG; E3
		971	2	1	0	0	0	1	0	1	1	1	s	rep C2OG; E7
		972	0	0	0	0	0	1	1	2	1	2	s; s	rep C1OG; TRG+SFG
		976	1	1	0	0	0	2	1	1	1	0	s	rep C2OG; E10
		981	0	0	1	0	0	2	1	1	1	1	t; s	rep C2OG; E3
		983	1	1	1	0	0	2	2	0	0	0	t; s; s	rep C1OG; AG+TRG
		984	0	0	2	0	0	2	0	1	1	1	s; s; t	rep C2OG; D2
		986	1	1	0	0	0	1	1	2	0	1	s	rep C2OG; E9
8	21	863	1	1	0	0	0	0	1	1	2	2	s; s	rep C2OG; E6
		864	1	1	0	0	0	0	2	1	1	2	d; s	non-rep C2OG; E6
		865	1	1	0	0	0	0	2	2	1	1	s; s	rep C2OG; E6
		866	1	1	1	0	0	0	0	1	2	2	t; t; s; s	rep C1OG; tick
		868	1	1	0	0	0	2	1	1	1	1	s	rep C2OG; F3
		869	1	1	0	0	0	1	1	0	2	2	s; s	rep C2OG; E8
		877	1	1	0	0	0	1	1	2	1	1	s	rep C2OG; F3
		880	1	1	0	0	0	2	1	1	1	1	s	rep C2OG; F3
		881	1	1	0	0	0	2	1	1	1	1	s; t	rep C2OG; F3
		882	0	0	0	0	0	1	1	2	2	2	s; s; s	rep C1OG; TRG+SFG
		883	2	1	0	0	0	2	1	0	1	1	s; s	rep C2OG; E8
		887	0	0	0	0	0	1	4	1	1	1	d(3)	non-rep C1OG; TRG+SFG
		891	2	1	0	0	0	1	1	1	1	1	s; t	rep C2OG; F3
		893	1	1	0	0	0	1	2	1	1	1	s	rep C2OG; F3
		898	1	1	0	0	0	2	1	1	1	1	s	rep C2OG; F3
		900	1	2	0	0	0	1	2	1	0	1	s; s	rep C2OG; E9
		903	0	0	0	0	0	1	1	2	2	2	s; s; s	rep C1OG; TRG+SFG
		909	0	0	1	0	0	2	1	2	1	1	s; s	rep C2OG; E3
		912	1	1	0	0	0	1	3	2	0	0	d(2); d	non-rep C2OG; D13
		914	1	2	0	0	0	1	1	1	1	1	d; t	non-rep C2OG; F3
		920	1	1	1	1	0	0	3	1	0	0	2 (Fe & pRF), no pRFδ	non-rep C2OG; E15
9	21	807	0	0	1	0	0	0	2	2	3	1	t; d; s; s(2)	non-rep C2OG; D1
		808	0	0	9	0	0	0	0	0	0	0	d(8)	non-rep C1OG (FS)
		809	1	1	1	0	0	0	3	1	1	1	d(2)	non-rep C2OG; F5
		810	1	1	1	0	0	0	1	2	2	1	t; s; s	rep C2OG; F5
		811	1	1	1	0	0	0	1	2	1	2	s; s	rep C2OG; F5
		812	1	1	1	0	0	0	1	2	1	2	t; s; s	rep C2OG; F5

		813	1	1	0	0	0	0	0	2	2	3	s; s; s(2)	rep C2OG; D7
		814	2	2	1	0	0	1	0	1	1	1	d; d; t	non-rep C2OG; F6
		816	1	2	1	0	0	1	1	1	1	1	s	rep C1OG; -TG
		817	1	1	1	0	0	2	0	2	1	1	s; s	rep C2OG; F6
		818	1	1	2	0	0	1	1	1	1	1	s	rep C1OG; -TG
		819	1	1	2	0	0	1	1	1	1	1	s	rep C1OG; -TG
		820	3	3	0	0	0	1	1	0	1	0	d(2); d(2); t	non-rep C2OG; D12
		821	1	1	0	0	0	2	1	2	1	1	s; s	rep C2OG; F3
		822	1	1	1	0	0	1	2	1	1	1	s	rep C1OG; -TG
		823	1	1	1	0	0	1	2	1	1	1	t; t; d	non-rep C1OG
		824	1	1	1	0	0	1	1	2	1	1	t; s	rep C1OG; -TG
		826	0	0	2	0	0	1	1	1	2	2	s; s; s	rep C2OG; E3
		829	1	1	0	0	0	1	1	2	2	1	s; s	rep C2OG; F3
		830	1	1	1	0	0	2	1	1	1	1	s	rep C1OG; -TG
		844	0	1	0	1	1	1	1	0	2	2	s; s	rep C2OG; F2
10	17	388	1	1	1	2	1	1	1	0	1	1	t; s	rep C2OG; H7
		449	1	1	0	1	1	1	1	1	2	1	t; s; t	rep C2OG; H3
		59	1	1	0	0	0	0	1	3	2	2	s(2); s; s	rep C2OG; E6
		60	1	1	1	0	0	0	1	2	2	2	t; s; s; s	rep C2OG; F5
		61	1	2	0	0	0	0	1	2	2	2	s; s; s; s	rep C2OG; E6
		62	1	1	1	0	0	2	1	2	1	1	t; s; s	rep C1OG; -TG
		63	1	1	1	0	0	1	2	1	2	1	t; d; s	non-rep C1OG
		64	1	2	0	0	0	1	1	2	1	2	s; s; s	rep C2OG; F3
		65	1	1	0	0	0	1	1	2	2	2	d; d; d	non-rep C2OG; F3
		66	1	1	0	0	0	1	1	2	2	2	t; s; s; s	rep C2OG; F3
		67	1	1	1	0	0	2	1	1	1	2	s; t; s	rep C1OG; -TG
		68	2	2	0	0	0	2	1	1	1	1	s; s; s; t	rep C2OG; F3
		69	1	1	0	0	0	1	2	2	3	0	t; s; s; s(2)	rep C2OG; E10
		70	1	1	0	0	0	2	1	2	2	1	s; s; s; t	rep C2OG; F3
		71	1	1	0	0	0	2	1	2	2	1	s; s; s	rep C2OG; F3
		72	1	1	1	0	0	3	1	1	1	1	t; s(2)	rep C1OG; -TG
		73	1	1	1	0	0	2	1	1	1	2	t; s; s	rep C1OG; -TG
11	24	35	1	1	0	0	0	0	1	2	3	3	s; s(2); s(2)	rep C2OG; E6
		36	1	1	1	0	0	0	1	2	3	2	t; s; s(2); s	rep C2OG; F5
		37	1	1	2	0	0	1	2	2	1	1	s; s; s	rep C1OG; -TG

		38	1	1	1	0	0	1	2	1	2	2	t; t; s; s; s	rep C1OG; -TG
		39	1	1	1	0	0	1	1	2	2	2	s; s; s	rep C1OG; -TG
		40	1	1	0	0	0	2	1	2	2	2	s; s; s; s	rep C2OG; F3
		41	1	1	0	0	0	1	2	2	2	2	s; s; s; s	rep C2OG; F3
		42	1	1	0	0	0	1	2	3	2	1	t; s; s; s	rep C2OG; F3
		43	1	2	1	1	1	1	1	1	1	1	s	rep C1OG; core
		44	1	1	2	1	1	1	1	1	1	1	s	rep C1OG; core
		45	1	1	1	1	1	1	1	1	1	2	s	rep C1OG; core
		46	1	1	1	1	2	1	1	1	1	1	d	non-rep C1OG; core
		47	1	2	1	1	1	1	1	1	1	1	s	rep C1OG; core
		48	1	1	2	1	1	1	1	0	1	2	s; s	rep C2OG; H7
		49	1	1	2	1	1	1	1	1	1	1	s	rep C1OG; core
		50	1	1	1	1	2	0	3	0	1	1	s; s(2); t; t	rep C2OG; G7
		51	1	1	1	1	1	1	1	0	2	2	s; s	rep C2OG; H7
		52	1	2	1	1	1	1	1	1	1	1	s	rep C1OG; core
		53	1	1	1	1	1	2	1	1	1	1	s	rep C1OG; core
		54	1	1	1	1	1	1	1	2	1	1	s	rep C1OG; core
		55	1	2	1	1	1	1	1	1	1	1	s	rep C1OG; core
		56	1	1	1	1	1	1	2	1	1	1	d	non-rep C1OG; core
		57	1	2	1	1	1	1	1	1	1	1	s	rep C1OG; core
		58	1	2	1	1	1	1	1	1	1	1	s	rep C1OG; core
12	14	21	1	1	2	0	0	0	1	3	2	2	s; s(2); s; s	rep C2OG; F5
		22	2	2	0	0	0	2	2	1	2	1	d; d; d; d; d	non-rep C2OG; F3
		23	0	0	0	0	1	1	2	3	2	3	s; s(2); s; s(2)	rep C2OG; E1
		24	1	1	2	0	0	2	2	0	2	2	s; s; s; s; s	rep C2OG; F7
		25	1	1	1	0	0	2	1	2	2	2	t; s; s; s; s	rep C1OG; -TG
		26	0	0	0	0	0	12	0	0	0	0	d(11)	non-rep C1OG (FS)
		27	0	0	0	0	0	12	0	0	0	0	d(11)	non-rep C1OG (FS)
		28	2	1	0	0	0	3	1	2	2	1	s; s(2); s; s	rep C2OG; F3
		29	2	2	1	1	1	1	1	1	1	1	s; s	rep C1OG; core
		30	1	3	1	1	1	1	1	1	1	1	s(2)	rep C1OG; core
		31	1	1	1	1	1	2	2	1	1	1	d; d	non-rep C1OG; core
		32	2	1	0	1	1	1	1	2	1	2	s; t; s; s	rep C2OG; H3
		33	1	1	2	1	1	1	2	1	1	1	d; d	non-rep C1OG; core
		34	1	1	1	2	1	2	1	1	1	1	t; t; s; s	rep C1OG; core

13	6	**15**	**1**	**1**	**2**	**0**	**0**	**3**	**1**	**2**	**1**	**2**	**s; s(2); s; s**	**rep C1OG; -TG**
		**16**	**2**	**2**	**2**	**0**	**0**	**2**	**1**	**1**	**2**	**1**	**s; s; s; s; s**	**rep C1OG; -TG**
		**17**	**2**	**1**	**1**	**0**	**0**	**2**	**1**	**2**	**2**	**2**	**s; t; s; s; s; s**	**rep C1OG; -TG**
		**18**	**2**	**1**	**1**	**0**	**0**	**1**	**2**	**2**	**2**	**2**	**s; t; s; s; s; s**	**rep C1OG; -TG**
		19	1	1	0	1	1	2	1	2	2	2	d; s; s; s	non-rep C2OG; H3
		**20**	**1**	**1**	**1**	**1**	**1**	**1**	**1**	**2**	**2**	**2**	**s; s; s**	**rep C1OG; core**
14	5	**10**	**1**	**1**	**2**	**0**	**0**	**1**	**1**	**3**	**4**	**1**	**s; t; s(2); s(3)**	**rep C1OG; -TG**
		11	2	2	0	0	0	1	3	2	2	2	d; d; d(2); d; d; d	non-rep C2OG; F3
		**12**	**2**	**1**	**0**	**0**	**0**	**3**	**1**	**2**	**2**	**3**	**s; s(2); s; s; s(2)**	**rep C2OG; F3**
		**13**	**2**	**1**	**2**	**0**	**0**	**3**	**1**	**1**	**2**	**2**	**s; s; s(2); s; s**	**rep C1OG; -TG**
		**14**	**1**	**1**	**2**	**1**	**1**	**2**	**1**	**1**	**2**	**2**	**s; s; s; s**	**rep C1OG; core**
15	3	7	1	14	0	0	0	0	0	0	0	0	d(13)	non-rep C1OG; bellii
		8	12	3	0	0	0	0	0	0	0	0	d(11); d(2)	non-rep C1OG; bellii
		**9**	**2**	**2**	**1**	**1**	**1**	**0**	**1**	**3**	**2**	**2**	**s; s; t; s(2); s; s**	**rep C2OG; H6**
17	1	6	0	0	0	0	0	0	17	0	0	0	d(16)	non-rep C1OG (FS)
18	3	3	0	18	0	0	0	0	0	0	0	0	d(17)	non-rep C1OG (FS)
		**4**	**1**	**2**	**1**	**0**	**0**	**3**	**1**	**4**	**3**	**3**	**s; s(2); s(3); s(2); s(2)**	**rep C1OG; -TG**
		**5**	**1**	**1**	**1**	**1**	**1**	**4**	**4**	**2**	**1**	**2**	**s(3); s(3); s; s**	**rep C1OG; core**
23	1	**2**	**1**	**1**	**3**	**0**	**1**	**3**	**1**	**4**	**4**	**5**	**t; t; s(2); t; s(2); s(3); s(3); s(4)**	**rep C2OG; H4**
31	1	1	2	1	0	0	0	1	27	0	0	0	d; 24 (s(3))	non-rep C2OG; C25

[1] Underscored numbers are described sequentially (left to right) under Designation.
[2] Taxon abbreviations are explained in the **Figure 1** legend.
[3] d = gene duplication; s = split gene; t = truncated gene.
[4] Status after concatenation of split ORFs and removal of redundant pRFδ ORFs: rep = representative, non-rep = non-representative, C1OG = class 1 OG, C2OG = class 2 OG, (S) = singleton, (FS) = false singleton. OGs made representative after curation are bolded. Names for C1OGs follow the description in **Figure 5** and **Figure 7**. Alphanumerics following C2OGs depict less conserved distributions of OG members across the rickettsial tree (see **Figure S2**).
