# Supplementary material for: Rickettsia Phylogenomics: Unwinding the Intricacies of Obligate Intracellular Life
Source: PLoS One. 2008 Apr 16;3(4):e2018. doi: 10.1371/journal.pone.0002018 (PMC2635572; doi:10.1371/journal.pone.0002018)
Supplement: Table S2 — Distribution and characterization of predicted ORFs within 259 non-representative OGs across ten rickettsial genomes, and the results after manual curation. (0.18 MB PDF) [file pone.0002018.s005.pdf]

Table S2. Seven hundred-fifty two core rickettsial OGs predicted across ten analyzed genomes.

A: RNA processing and modification (6; 1%)

RiOG[1]	Annotation
300	ATP-dependent RNA helicase dbp2
767	glucose-inhibited division protein A
166	Maturase K
204	RNA modification enzyme, MiaB family
304	RNA methyltransferase, TrmH family, group 3
434	YihY family protein

B: Chromatin structure and dynamics (3; <1%)

RiOG[1]	Annotation
536	Centrosome protein 55
679	DNA-binding protein HU
414	DNA-binding protein HU-like

C: Energy production and conversion (67; 9%)

RiOG[1]	Annotation
58	Acyl-CoA desaturase
212	aconitate hydratase 1
793	ATP synthase F0, A subunit
208	ATP synthase F1, alpha subunit
210	ATP synthase F1, beta subunit
209	ATP synthase F1, gamma subunit
207	ATP synthase F1, delta subunit
211	ATP synthase F1, epsilon subunit
792	ATP synthase protein I
794	ATP synthase protein 9, mitochondrial
795	ATP synthase B' chain
796	ATP synthase B chain, sodium ion specific
527	Citrate-proton symporter
175	citrate synthase I
600	Cytochrome b
217	Cytochrome c biogenesis ATP-binding export protein ccmA
365	Cytochrome c-type biogenesis protein ccmE
30	Cytochrome c-type biogenesis protein cycK
599	Cytochrome c1, heme protein, mitochondrial precursor
613	Cytochrome c
662	Cytochrome c oxidase subunit 3
643	Cytochrome d ubiquinol oxidase subunit I
642	cytochrome d ubiquinol oxidase, subunit II
206	dihydrolipoamide dehydrogenase
189	Ferredoxin-2
655	2Fe-2S ferredoxin
762	iron-sulfur cluster assembly accessory protein
442	FeS cluster assembly scaffold IscU
443	iron-sulfur cluster assembly accessory protein
303	fumarate hydratase, class II
364	Inorganic pyrophosphatase
387	2-polyprenyl-6-methoxyphenol 4-hydroxylase

462	dihydrolipoamide dehydrogenase
469	isopentenyl-diphosphate delta-isomerase, type 2
478	Glycerol-3-phosphate dehydrogenase [NAD(P)+]
133	NADH dehydrogenase [ubiquinone] 1 alpha subcomplex subunit 12
542	NADH (or F420H2) dehydrogenase, subunit C
543	NADH dehydrogenase I, D subunit
541	NADH-quinone oxidoreductase chain A
117	NADH-quinone oxidoreductase, B subunit
544	NADH-quinone oxidoreductase, E subunit
593	NAD(P)H-quinone oxidoreductase chain 5, chloroplast
724	NADH-quinone oxidoreductase, F subunit
214	NADH-quinone oxidoreductase, chain G
216	NADH-quinone oxidoreductase, chain I
220	NADH-quinone oxidoreductase chain J
137	NADH-quinone oxidoreductase chain K
215	NADH-ubiquinone oxidoreductase chain 1
75	proton-translocating NADH-quinone oxidoreductase, chain N
218	proton-translocating NADH-quinone oxidoreductase, chain M
108	NAD(P) transhydrogenase, mitochondrial precursor
751	NAD(P) transhydrogenase, mitochondrial precursor
161	NAD(P) transhydrogenase subunit alpha
528	phosphate acetyltransferase
44	Phosphate acetyltransferase
609	Pyruvate dehydrogenase E1 component subunit alpha, mitochondrial precursor
608	Pyruvate dehydrogenase E1 component subunit beta, mitochondrial precursor
409	pyruvate dehydrogenase complex dihydrolipoamide acetyltransferase
671	2-oxoglutarate dehydrogenase, E1 component
672	2-oxoglutarate dehydrogenase, E2 component, dihydrolipoamide succinyltransferase
712	succinate dehydrogenase, flavoprotein subunit
713	succinate dehydrogenase, hydrophobic membrane anchor protein
714	succinate dehydrogenase, cytochrome b556 subunit
777	succinate dehydrogenase and fumarate reductase iron-sulfur protein
487	succinyl-CoA synthetase, alpha subunit
486	succinyl-CoA synthetase, beta subunit
763	3-deoxy-8-phosphooctulonate synthase
601	ubiquinol-cytochrome c reductase, iron-sulfur subunit
338	ubiquinone biosynthesis O-methyltransferase
291	ubiquinone/menaquinone biosynthesis methyltransferases

cf: Combined functions (22; 3%)

**RiOG[1]**	**Annotation**
347	ribonuclease P protein component; (**A** and **J**)
198	tyrosine recombinase XerC; (**B** and **D**)
538	tyrosine recombinase XerD; (**B** amd **D**)
605	isocitrate dehydrogenase; (**C** and **E**)
526	malate dehydrogenase, NAD-dependent; (**C** and **E**)
219	proton-translocating NADH-quinone oxidoreductase, chain L; (**C** and **P**)
489	dihydrodipicolinate synthase; (**E** and **M**)
379	COG0591: Na+/proline symporter; (**E** and **R**)
803	COG0493: NADPH-dependent glutamate synthase beta chain and related oxidoreductases; (**E** and **R**)
694	Amino-acid-binding protein aabA precursor; (**E** and **T**)
523	3-deoxy-D-manno-octulosonate cytidylyltransferase; (**G** and **M**)
292	2-polyprenylphenol 6-hydroxylase; (**H** and **C**)
243	acyl carrier protein; (**I** and **Q**)

359	transcription-repair coupling factor; (**L** and **K**)
88	Phosphatidate cytidylyltransferase; (**M** and **I**)
91	COG0526: Thiol-disulfide isomerase and thioredoxins; (**O** and **C**)
475	thioredoxin-disulfide reductase; (**O** and **C**)
412	COG0500: SAM-dependent methyltransferases; (**Q** and **R**)
742	COG0500: SAM-dependent methyltransferases; (**Q** and **R**)
283	ATP-dependent Clp protease, ATP-binding subunit ClpX; (**U** and **O**)
583	Guanosine-5'-triphosphate,3'-diphosphate pyrophosphatase; (**T** and **K**)
202	BolA protein homolog; (**V** and **D**)

D: Cell cycle control, mitosis and meiosis (11; 2%)

**RiOG[1]**	**Annotation**
614	cell division protein FtsA
194	DNA translocase ftsK
382	Cell division protein FtsL
110	Cell division protein ftsQ homolog
76	cell division protein FtsW
302	cell division protein FtsZ
698	Chromosome partitioning protein
766	ParB-like partition proteins
516	intracellular septation protein A
200	septum formation protein Maf
493	COG2919: Septum formation initiator

E: Amino acid transport and metabolism (19; 3%)

**RiOG[1]**	**Annotation**
573	amino acid transporter
675	Excitatory amino acid transporter 2
711	amino acid ABC transporter, permease protein, 3-TM region, His/Glu/Gln/Arg/opine family
743	Aspartate aminotransferase
251	aspartate kinase
567	aspartate-semialdehyde dehydrogenase
441	cysteine desulfurase IscS
181	DAP2-like protein
84	diaminopimelate epimerase
695	dihydrodipicolinate reductase
435	Glutamine synthetase
157	Glutamine transport ATP-binding protein glnQ
248	NAD-specific glutamate dehydrogenase
127	Nitrogen assimilation regulatory protein ntrX
444	putrescine-ornithine antiporter
261	Serine hydroxymethyltransferase 2
152	succinyl-diaminopimelate desuccinylase
659	2,3,4,5-tetrahydropyridine-2,6-dicarboxylate N-succinyltransferase
471	Threonine dehydratase biosynthetic

F: Nucleotide transport and metabolism (17; 2%)

**RiOG[1]**	**Annotation**
325	adenylate kinases
431	ADP/ATP carrier protein family
448	ADP/ATP carrier protein family
525	ADP/ATP carrier protein family

264	ADP/ATP carrier protein family
770	ADP/ATP carrier protein family
524	CTP synthase
415	cytidylate kinase
187	cytosine deaminase
756	deoxycytidine triphosphate deaminase
241	Guanylate kinase
768	Nucleoside diphosphate kinase
639	Phosphoribosylaminoimidazole-succinocarboxamide synthase
120	thymidylate kinase
578	thymidylate synthase, flavin-dependent
510	dUTP diphosphatase
689	uridylate kinase

G: Carbohydrate transport and metabolism (15; 2%)

RiOG[1]	Annotation
276	Beta-hexosaminidase A precursor
551	Capsular polysaccharide biosynthesis glycosyl transferase capM
555	Capsular polysaccharide biosynthesis protein capD
556	dTDP-4-dehydrorhamnose reductase
369	HAD-superfamily subfamily IIA hydrolase, TIGR01459
623	Inositol-1-monophosphatase
424	Phosphomannomutase
33	Proline/betaine transporter
147	Proline/betaine transporter
249	Proline/betaine transporter
287	proline/betaine transporter
749	Proline/betaine transporter
437	pyruvate, phosphate dikinase
427	sugar isomerase, KpsF/GutQ family
769	Sugar phosphate permease
579	sugar-phosphate isomerases, RpiB/LacA/LacB family

H: Coenzyme transport and metabolism (19; 3%)

RiOG[1]	Annotation
178	5-aminolevulinic acid synthase
402	Delta-aminolevulinic acid dehydratase
406	biotin--acetyl-CoA-carboxylase ligase
463	biotin synthesis protein BioC
113	Coproporphyrinogen III oxidase, mitochondrial precursor
143	dephospho-CoA kinase
145	ferrochelatase
404	FolC bifunctional protein
419	FolD bifunctional protein
85	GTP cyclohydrolase I
151	lipoate-protein ligase B
142	lipoic acid synthetase
446	Octaprenyl-diphosphate synthase
286	4-hydroxybenzoate polyprenyl transferase
400	polyprenyl P-hydroxybenzoate and phenylacrylic acid decarboxylases
457	porphobilinogen deaminase
673	COG0720: 6-pyruvoyl-tetrahydropterin synthase
663	Ubiquinone biosynthesis protein COQ7 homolog
116	uroporphyrinogen decarboxylase

I: Lipid transport and metabolism (16; 2%)

RiOG[1]	Annotation
3455	1-acyl-sn-glycerol-3-phosphate acyltransferase 1, chloroplast precursor
40	acylglycerophosphoethanolamine acyltransferase
801	beta-hydroxyacyl-(acyl-carrier-protein) dehydratase FabZ
773	CDP-diacylglycerol--glycerol-3-phosphate 3-phosphatidyltransferase
620	CDP-diacylglycerol--serine O-phosphatidyltransferase
78	Dolichol kinase
535	Enoyl-[acyl-carrier-protein] reductase [NADH]
519	Lipid A export ATP-binding/permease protein msbA
265	malonyl CoA-acyl carrier protein transacylase
155	COG0671: Membrane-associated phospholipid phosphatase
254	Phosphatidylglycerophosphatase A
373	phosphopantethiene--protein transferase domain
621	phosphatidylserine decarboxylase homolog
290	polyhydroxyalkanoate depolymerase, intracellular
341	Propionyl-CoA carboxylase beta chain, mitochondrial precursor
342	Propionyl-CoA carboxylase alpha chain, mitochondrial precursor

J: Translation

RiOG[1]	Annotation (118; 16%)
119	Sua5/YciO/YrdC/YwlC family protein
173	pseudouridine synthase, RluA family
193	methionine aminopeptidase, type I
233	Small protein A homolog precursor
460	Ribonuclease D
494	COG0349: Ribonuclease D
334	ribonuclease PH
344	rpsU-divergently transcribed protein
353	peptidyl-tRNA hydrolase
410	peptide chain release factor 1
597	peptide chain release factor 2
134	peptide deformylase
418	ribosomal subunit interface protein
447	Mitochondrial ribosomal protein VAR1
92	SsrA-binding protein
484	ribosome-binding factor A
548	16S rRNA processing protein RimM
690	ribosome recycling factor
692	glutamyl-tRNA(Gln) and/or aspartyl-tRNA(Asn) amidotransferase, A subunit
693	glutamyl-tRNA(Gln) and/or aspartyl-tRNA(Asn) amidotransferase, B subunit
691	glutamyl-tRNA(Gln) and/or aspartyl-tRNA(Asn) amidotransferase, C subunit
757	COG0154: Asp-tRNAAsn/Glu-tRNAGln amidotransferase A subunit, related amidases
428	Polyribonucleotide nucleotidyltransferase
172	methyltransferase, HemK family
684	Ribosomal RNA large subunit methyltransferase J
646	S-adenosylmethionine:tRNA ribosyltransferase-isomerase
298	dimethyladenosine transferase
273	queuine tRNA-ribosyltransferase
498	MiaB-like tRNA modifying enzyme
416	ribosomal protein S1
746	ribosomal protein S2
312	ribosomal protein S3

550	ribosomal protein S4
321	ribosomal protein S5
782	ribosomal protein S6
709	ribosomal protein S7
318	30S ribosomal protein S8
627	30S ribosomal protein S9
306	ribosomal protein S10
327	30S ribosomal protein S11
710	ribosomal protein S12
326	30S ribosomal protein S13
317	30S ribosomal protein S14
429	ribosomal protein S15
150	ribosomal protein S16
314	30S ribosomal protein S17
781	ribosomal protein S18
310	ribosomal protein S19
330	ribosomal protein S20
345	ribosomal protein S21
704	ribosomal protein L1
309	ribosomal protein L2
307	50S ribosomal protein L3
308	50S ribosomal protein L4
316	50S ribosomal protein L5
319	50S ribosomal protein L6
702	ribosomal protein L7/L12
780	ribosomal protein L9
703	50S ribosomal protein L10
705	ribosomal protein L11
628	ribosomal protein L13
315	ribosomal protein L14
323	ribosomal protein L15
313	ribosomal protein L16
329	ribosomal protein L17
320	ribosomal protein L18
727	ribosomal protein L19
349	ribosomal protein L20
253	ribosomal protein L21
311	ribosomal protein L22
139	50S ribosomal protein L23
352	ribosomal protein L25, Ctc-form
252	ribosomal protein L27
735	ribosomal protein L28
140	ribosomal protein L29
322	ribosomal protein L30
97	50S ribosomal protein L31
234	ribosomal protein L32
149	ribosomal protein L33
348	ribosomal protein L34
350	ribosomal protein L35
466	50S ribosomal protein L36
47	alanyl-tRNA synthetase
760	arginyl-tRNA synthetase
697	aspartyl-tRNA synthetase
747	cysteinyl-tRNA synthetase
337	glutamyl-tRNA synthetase
562	glutamyl-tRNA synthetase

170	glycyl-tRNA synthetase, alpha subunit
171	glycyl-tRNA synthetase, beta subunit
572	histidyl-tRNA synthetase
343	isoleucyl-tRNA synthetase
55	leucyl-tRNA synthetase
779	tRNA(Ile)-lysidine synthetase
530	lysyl-tRNA synthetase
288	methionyl-tRNA synthetase
648	methionyl-tRNA formyltransferase
82	phenylalanyl-tRNA synthetase, alpha subunit
497	phenylalanyl-tRNA synthetase, beta subunit
522	prolyl-tRNA synthetase
226	seryl-tRNA synthetase
638	threonyl-tRNA synthetase
389	tyrosyl-tRNA synthetase
456	tryptophanyl-tRNA synthetase
285	valyl-tRNA synthetase
728	tRNA (guanine-N1)-methyltransferase
798	tRNA-nucleotidyltransferase 1, mitochondrial precursor
165	tRNA pseudouridine synthase A
247	tRNA modification GTPase TrmE
423	tRNA delta(2)-isopentenylpyrophosphate transferase
430	tRNA pseudouridine synthase B
458	tRNA-modifying protein ygfZ
574	tRNA (5-methylaminomethyl-2-thiouridylate)-methyltransferase
708	translation elongation factor G
624	translation elongation factor P
201	translation initiation factor IF-1
393	translation initiation factor IF-2
408	translation initiation factor IF-3
745	translation elongation factor Ts
305	translation elongation factor Tu

K: Transcription (14; 2%)

RiOG[1]	Annotation
299	Cold shock-like protein 7.0
328	DNA-directed RNA polymerase, alpha subunit
700	DNA-directed RNA polymerase, beta'' subunit
701	DNA-directed RNA polymerase, beta subunit
372	DNA-directed RNA polymerase, omega subunit
162	transcription elongation factor GreA
199	RNA polymerase-binding protein DksA
164	RNA polymerase sigma factor RpoD
754	Transcriptional regulatory protein pmrA
706	transcription termination/antitermination factor NusG
93	transcription antitermination factor NusB
392	transcription termination factor NusA
413	transcription termination factor Rho
576	alternative sigma factor RpoH

L: Replication, recombination and repair (52; 7%)

RiOG[1]	Annotation
331	Bacterial chromosomal replication initiator protein, family
356	chromosomal replication initiator protein DnaA

520	Holliday junction DNA helicase RuvA
89	Holliday junction DNA helicase RuvB
721	crossover junction endodeoxyribonuclease RuvC
148	DNA mismatch repair protein MutL
580	DNA mismatch repair protein MutS
231	DNA polymerase I
230	DNA polymerase III, alpha subunit
496	DNA polymerase III, beta subunit
46	DNA polymerase III, subunits gamma and tau
678	DNA polymerase III subunit delta
664	COG1466: DNA polymerase III, delta subunit
268	DNA polymerase III, epsilon subunit
153	COG2927: DNA polymerase III, chi subunit
163	DNA primase
385	DNA double-strand break repair rad50 ATPase
20	DNA double-strand break repair rad50 ATPase
396	DNA repair protein RadA
245	recA protein
789	DNA replication and repair protein RecF
52	COG1200: RecG-like helicase
411	single-stranded-DNA-specific exonuclease RecJ
395	DNA repair protein RecO
670	DNA repair protein RecN
472	COG0419: ATPase involved in DNA repair
481	recombination protein RecR
284	DNA recombination protein rmuC homolog
183	excinuclease ABC, A subunit
652	excinuclease ABC, B subunit
378	excinuclease ABC, C subunit
294	exodeoxyribonuclease III
610	exodeoxyribonuclease III
295	exodeoxyribonuclease VII, large subunit
546	exodeoxyribonuclease VII, small subunit
258	endonuclease III
266	ATP-dependent helicase
399	replicative DNA helicase
473	ATP-dependent DNA helicase PcrA
271	Ribonuclease HI
653	Ribonuclease HII
95	ribonuclease III
274	DNA ligase, NAD-dependent
289	hydrolase, TatD family
649	DNA gyrase, A subunit
370	DNA gyrase, B subunit
401	primosomal protein N''
421	ribonucleoside-diphosphate reductase, alpha subunit
422	Ribonucleoside-diphosphate reductase small chain B
545	DNA-3-methyladenine glycosylase
561	DNA topoisomerase I
758	DNA topoisomerase IV, A subunit
633	DNA topoisomerase IV, B subunit
174	uracil-DNA glycosylase, family 4
182	single-strand binding protein

M: Cell wall/membrane biogenesis (63; 9%)

RiOG[1]	Annotation
611	D-alanyl-D-alanine carboxypeptidase precursor
615	D-alanine--D-alanine ligase
739	alanine racemase
81	HtrB proetin
103	Glycosyltransferase
474	Glycosyltransferase
278	COG0438: Glycosyltransferase
499	COG0438: Glycosyltransferase
109	Membrane protein
176	GTP-binding protein Obg/CgtA
722	GTP-binding protein Era
596	GTP-binding protein LepA
607	GTP-binding protein TypA
354	GTP-binding protein YchF
277	outer membrane autotransporter barrel domain
549	Outer membrane assembly protein
636	Outer membrane protein tolC precursor
686	Outer membrane protein assembly factor yaeT precursor
797	outer membrane autotransporter barrel domain
185	17 kDa surface antigen precursor
339	29 kDa membrane protein in psaA 5'region
191	Opacity protein, putative
192	Phospho-N-acetylmuramoyl-pentapeptide-transferase
205	penicillin-binding protein, 1A family
383	Penicillin-binding protein 2
384	Penicillin-binding protein
518	penicillin-binding protein dacF precursor
236	peptidoglycan-associated lipoprotein
279	lipoprotein releasing system, transmembrane protein, LolC/E family
517	rare lipoprotein A
534	apolipoprotein N-acyltransferase
100	prolipoprotein diacylglyceryl transferase
641	Lipopolysaccharide core biosynthesis glycosyl transferase kdtX
540	COG3307: Lipid A core - O-antigen ligase and related enzymes
238	cell shape determining protein, MreB/Mrl family
239	rod shape-determining protein MreC
595	rod shape-determining protein RodA
242	3-oxoacyl-[acyl-carrier-protein] synthase 2
235	3-oxoacyl-(acyl-carrier-protein) synthase III
244	3-oxoacyl-(acyl-carrier-protein) reductase
275	tetraacyldisaccharide 4''-kinase
296	Organic solvent tolerance protein precursor
505	Membrane proteins related to metalloendopeptidases
685	membrane-associated zinc metalloprotease, putative
362	phospho-N-acetylmuramoyl-pentapeptide-transferase
371	UDP-N-acetylglucosamine 1-carboxyvinyltransferase
360	UDP-N-acetylmuramyl-tripeptide synthetases
361	UDP-N-acetylmuramoyl-tripeptide--D-alanyl-D-alanine ligase
229	UDP-glucose 6-dehydrogenase
112	UDP-N-acetylenolpyruvoylglucosamine reductase
104	UDP-N-acetylglucosamine 2-epimerase
502	UDP-N-acetylmuramoylalanine--D-glutamate ligase
612	UDP-3-0-acyl N-acetylglucosamine deacetylase
616	UDP-N-acetylmuramate--alanine ligase

800	UDP-3-O-[3-hydroxymyristoyl] glucosamine N-acyltransferase
802	acyl-[acyl-carrier-protein]--UDP-N-acetylglucosamine O-acyltransferase
381	S-adenosyl-methyltransferase MraW
468	Bifunctional protein glmU
492	undecaprenyl diphosphate synthase
501	undecaprenyldiphospho-muramoylpentapeptide beta-N-acetylglucosaminyltransferase
509	COG0741: Soluble lytic murein transglycosylase and related regulatory proteins (some contain LysM/invasin domains)
634	Vegetative cell wall protein gp1 precursor
101	Periplasmic protein TonB

N: Cell motility (1; <1%)

RiOG[1]	Annotation
454	COG5622: Protein required for attachment to host cells

O: Posttranslational modification, protein turnover, chaperones (36; 5%)

RiOG[1]	Annotation
136	Fe-S protein assembly co-chaperone HscB
335	10 kDa chaperonin 5
106	ATP-dependent protease hslV
417	ATP-dependent Clp protease, proteolytic subunit ClpP
784	Chaperone clpB
668	chaperone protein DnaJ
654	Chaperone protein dnaK
667	chaperone protein DnaK
336	chaperonin GroEL
179	Chaperone protein htpG
632	carboxyl-terminal protease
470	ATP-dependent protease La
559	Membrane protease subunit, stomatin/prohibitin-like protein
565	heat shock protein HslVU, ATPase subunit HslU
594	Protease 2
666	heat shock protease
716	protease Do
177	trigger factor
445	Aminopeptidase P
195	COG0760: Parvulin-like peptidyl-prolyl isomerase
560	Peroxiredoxin-1
259	glutaredoxin homolog
651	glutaredoxin 3
438	Glutathione S-transferase
297	Parvulin-like peptidyl-prolyl isomerase, putative
531	Disulfide bond formation protein DsbB
791	Protein-disulfide isomerase
488	COG1651: Protein-disulfide isomerase
513	COG1047: FKBP-type peptidyl-prolyl cis-trans isomerases 2
301	HIRA-interacting protein 5
333	Protein grpE
351	Organic radical activating enzyme
374	Protein export protein PrsA precursor
420	Thioredoxin reductase
805	thioredoxin
778	ATP-dependent metalloprotease FtsH

P: Inorganic ion transport and metabolism (14; 2%)

RiOG[1]	Annotation
144	Metal ABC transporter substrate-binding lipoprotein precursor
184	Ferric cations import ATP-binding protein fbpC
186	cation diffusion facilitator family transporter
223	Tellurium resistance protein terC
368	magnesium transporter
405	Superoxide dismutase [Fe]
515	FTR1 family protein
564	lipid-A-disaccharide synthase
604	monovalent cation/proton antiporter, MnhG/PhaG subunit
94	COG1863: Multisubunit Na+/H+ antiporter, MnhE subunit
603	Na(+)/H(+) antiporter subunit B
590	Na(+)/H(+) antiporter subunit C
591	Na(+)/H(+) antiporter subunit D
592	Na(+)/H(+) antiporter subunit D

Q: Secondary metabolites biosynthesis, transport and catabolism (2; <1%)

RiOG[1]	Annotation
14	metabolite:proton symporter family protein
512	Protocatechuate 3,4-dioxygenase beta chain
740	ABC-type transport system involved in resistance to organic solvents, auxiliary component

R: General function prediction only (67; 9%)

RiOG[1]	Annotation
256	ABC-type transport systems periplasmic component
272	ABC transporter substrate binding protein
425	Probable ABC transporter ATP-binding protein in ntrA/rpoN 5'region
532	ABC transporter permease protein
533	ABC transporter substrate binding protein, family
738	ABC transporter permease protein
765	ABC transporter, putative
293	small GTP-binding protein domain
734	GTP-binding conserved hypothetical protein
29	GTP pyrophosphokinase
390	hemolysin A
263	Hemolysin C
197	Phospholipase D (PLD) superfamily protein
355	Patatin B1 precursor
547	Extracellular matrix protein 33 precursor
190	Putative outer surface protein
606	Outer membrane protein, putative
160	Outer membrane lipoprotein-sorting protein, putative
5	outer membrane autotransporter barrel domain
34	outer membrane autotransporter barrel domain
750	Putative outer membrane protein RP075 precursor
483	Predicted membrane protein
786	Putative colicin V production membrane protein
363	integral membrane protein MviN
45	putative TIM-barrel protein, nifR3 family
167	COG0687: Spermidine/putrescine-binding periplasmic protein
83	Probable transcriptional regulatory protein silR

240	Predicted transcriptional regulator
282	exsB protein
790	putative CarD-like transcriptional regulator
74	Bacterial SH3-like region, putative
154	Predicted permeases
237	Predicted permeases
332	Putative permease perM homolog
477	Predicted permease
529	Predicted permease
657	TRAP transporter solute receptor, TAXI family
775	Small-conductance mechanosensitive channel, putative
99	chromosome partitioning protein, putative
453	COG2945: Predicted hydrolase of the alpha/beta superfamily
552	Predicted hydrolase or acyltransferase
156	Metal-dependent hydrolase of the beta-lactamase superfamily I
626	Probable (di)nucleoside polyphosphate hydrolase
53	Predicted esterase of the alpha-beta hydrolase superfamily
761	deoxyguanosinetriphosphate triphosphohydrolase, putative
115	COG0457: FOG: TPR repeat
465	invasion protein homolog
105	Protein cyaY
440	Probable cysteine desulfurase 1
783	metalloendopeptidase, putative, glycoprotease family
87	Nitrilotriacetate monooxygenase component B
132	putative ribosomal-protein-alanine N-acetyltransferase
196	UbiD family decarboxylases
260	Carboxylesterase 1
508	LD-carboxypeptidase A, putative
56	Protein bioY
366	Protein senC
676	putative oxygen-independent coproporphyrinogen III oxidase
682	Protein COQ10 B, mitochondrial precursor
394	Putative glycoprotein endopeptidase
432	Antigenic heat-stable 120 kDa protein
436	Putative aldolase class 2 protein CC1201
461	5,10-methenyltetrahydrofolate synthetase
480	Probable inorganic polyphosphate/ATP-NAD kinase
490	Putative branched-chain-amino-acid aminotransferase
511	Putative signal peptide peptidase sppA
575	Lactosylceramide 4-alpha-galactosyltransferase
598	Probable Hsp20-family chaperone
647	Putative ATPase n2B
650	ATP-binding cassette sub-family B member 7, mitochondrial precursor
660	MJ0042 family finger-like domain
699	Probable cytosol aminopeptidase
737	Probable ribonucleotide transport ATP-binding protein mkl
54	Ran-binding protein 2-like 4

rpe: Rickettsial palindromic element-containing protein (19; 1%)

**RiOG[1]**	**Annotation**
77	Rickettsial palindromic element (RPE) domain
90	Rickettsial palindromic element (RPE) domain
397	Rickettsial palindromic element (RPE) domain
451	Rickettsial palindromic element (RPE) domain
506	Rickettsial palindromic element (RPE) domain

507	Rickettsial palindromic element (RPE) domain
719	Rickettsial palindromic element (RPE) domain
720	Rickettsial palindromic element (RPE) domain
744	Rickettsial palindromic element (RPE) domain
776	Rickettsial palindromic element (RPE) domain

S: Function unknown (93; 13%)

RiOG[1]	Annotation
31	Hypothetical protein
43	Hypothetical protein
107	hypothetical protein
114	Hypothetical protein
121	Hypothetical protein
126	Hypothetical protein
128	Hypothetical protein
135	Hypothetical protein
141	Hypothetical protein
168	Hypothetical protein
169	Hypothetical protein
180	Hypothetical protein
221	Hypothetical protein
222	Hypothetical protein
224	Hypothetical protein
250	Hypothetical protein
280	Hypothetical protein
357	hypothetical protein
376	Hypothetical protein
386	Hypothetical protein
426	Hypothetical protein
433	hypothetical protein
439	Hypothetical protein
464	Hypothetical protein
476	Hypothetical protein
479	Hypothetical protein
482	Hypothetical protein
485	Hypothetical protein
495	Hypothetical protein
500	Hypothetical protein
503	Hypothetical protein
514	Hypothetical protein
553	Hypothetical protein
566	Hypothetical protein
581	hypothetical protein
587	Hypothetical protein
618	hypothetical protein
622	Hypothetical protein
635	Hypothetical protein
637	Hypothetical protein
640	hypothetical protein
644	Hypothetical protein
656	Hypothetical protein
665	Hypothetical protein
683	Hypothetical protein
687	Hypothetical protein
696	hypothetical protein

736	Hypothetical protein
752	Hypothetical protein
759	Hypothetical protein
764	Hypothetical protein
785	Hypothetical protein
398	conserved hypothetical protein
79	conserved hypothetical protein TIGR00726
102	conserved hypothetical protein TIGR00278
146	conserved hypothetical protein TIGR00701
158	conserved hypothetical protein TIGR02281
159	conserved hypothetical protein TIGR00103
257	conserved hypothetical protein TIGR00096
262	conserved hypothetical protein TIGR00043
467	conserved hypothetical protein TIGR01033
557	conserved hypothetical protein TIGR00250
563	conserved hypothetical protein TIGR00247
799	conserved hypothetical protein TIGR00150
681	conserved hypothetical protein TIGR00046
391	UPF0090 protein yhbC
669	UPF0169 lipoprotein Xf0938 precursor
715	UPF0176 protein RHE_CH03638
726	UPF0335 protein RPA4190
787	UPF0301 protein SPO0296
806	UPF0085 protein Erum2050/ERWE_CDS_02070
558	UPF0141 inner membrane protein yhbX
130	UPF0053 protein HI0107
111	COG1434: Uncharacterized conserved protein
213	COG3671: Predicted membrane protein
269	COG3494: Uncharacterized protein conserved in bacteria
270	COG3814: Uncharacterized protein conserved in bacteria
377	COG2847: Uncharacterized protein conserved in bacteria
407	COG5346: Predicted membrane protein
539	COG4765: Uncharacterized protein conserved in bacteria
537	Uncharacterized protein L183
741	Uncharacterized protein RC0123 precursor
771	Uncharacterized lipoprotein RC0077 precursor
772	Uncharacterized protein RP050 precursor
521	unnamed protein product
358	Protein of unknown function DUF339, putative
459	Protein C6orf96
617	DUF2-containing protein
129	iojap homolog
228	Asparagine-rich protein
380	Protein mraZ
403	Myosin heavy chain, clone 203
582	Toxin B
629	WD40-like repeat
753	NADPH-dependent 7-cyano-7-deazaguanine reductase

T: Transduction mechanisms (9; 1%)

RiOG[1]	Annotation
227	AmpG protein
450	AmpG-related permease
346	Nitrogen regulation protein ntrY homolog
491	Osmolarity sensor protein EnvZ

631	Sensor protein gacS
625	diguanylate cyclase (GGDEF) domain
658	Universal stress protein UspA
246	COG0642: Signal transduction histidine kinase
788	COG0643: Chemotaxis protein histidine kinase and related kinases

U: Intracellular trafficking and secretion (46; 6%)

RiOG[1]	Annotation
661	VirB2-like protein
733	Type IV secretory system protein VirB3, putative
225	type IV secretion/conjugal transfer ATPase, VirB4 family
732	type IV secretion/conjugal transfer ATPase, VirB4 family
584	Protein virD4
729	Protein virB6
96	TrbL/VirB6 plasmid Conjugative transfer protein
730	COG3704: Type IV secretory pathway, VirB6 components
731	TrbL/VirB6 plasmid Conjugative transfer protein
57	COG3704: Type IV secretory pathway, VirB6 components
586	VirB8
588	VirB8 protein
589	P-type conjugative transfer protein VirB9
123	VirB9 protein precursor
585	Protein virB10
122	P-type DNA transfer ATPase VirB11
125	COG1175: ABC-type sugar transport systems, permease components
602	COG0747: ABC-type dipeptide transport system, periplasmic component
80	preprotein translocase, YajC subunit
86	O-antigen export system permease protein rfbA
804	O-antigen export system ATP-binding protein rfbB
98	S-adenosylmethionine transporter
124	Heme exporter protein B
131	Lipoprotein-releasing system ATP-binding protein lolD
188	heme exporter protein CcmC
267	Surfeit locus protein 1
554	efflux transporter, RND family, MFP subunit
138	Sec-independent protein translocase TatC
255	twin-arginine translocation protein, TatA/E family
568	type I secretion system ATPase
569	type I secretion membrane fusion protein, HlyD family
571	protein TolQ
570	protein TolR
577	Tol-Pal system beta propeller repeat protein TolB
630	tol-pal system protein YbgF
232	signal recognition particle-docking protein FtsY
677	signal recognition particle protein
723	signal peptidase I
504	signal peptidase II
375	preprotein translocase, SecA subunit
755	protein-export protein SecB
367	protein-export membrane protein SecD
707	preprotein translocase, SecE subunit
725	protein-export membrane protein SecF
748	preprotein translocase, SecG subunit
324	preprotein translocase, SecY subunit
774	Inner membrane protein oxaA

V: Defense mechanisms (11; 2%)

RiOG[1]	Annotation
717	HflC protein
718	HflK protein
452	HemY-like protein
118	Multisubunit Na+/H+ antiporter, MnhF subunit
203	potassium efflux system protein
688	drug resistance transporter, EmrB/QacA subfamily
49	Bicyclomycin resistance protein
281	Multidrug resistance protein 3
619	Multidrug resistance protein A
645	Multidrug resistance ABC transporter ATP-binding protein
674	Divalent-cation tolerance protein cutA
680	Hydrophobe/amphiphile efflux-1 HAE1 family protein

[1]Underscored RiOGs depict non-representative OGs.
