# Supplementary material for: Rickettsia Phylogenomics: Unwinding the Intricacies of Obligate Intracellular Life
Source: PLoS One. 2008 Apr 16;3(4):e2018. doi: 10.1371/journal.pone.0002018 (PMC2635572; doi:10.1371/journal.pone.0002018)
Supplement: Table S3 — Seven hundred-fifty two core rickettsial OGs predicted across ten analyzed genomes. (0.19 MB PDF) [file pone.0002018.s006.pdf]

Table S3. OGs present only in the *R. bellii* genomes.

OG[1]	Annotation (312)[2]
1848	Angiopoietin-1 precursor
1783	ATPase (AAA+ superfamily)-like
1768	ATP-dependent Clp protease proteolytic subunit
1789	ATP-dependent Clp protease proteolytic subunit
1797	Bifunctional penicillin-binding protein 1C
1996	Caldesmon
1713	Cassette chromosome recombinase B
1948	COG0477: Permeases of the major facilitator superfamily
1773	Deoxyribodipyrimidine photo-lyase
1817	DNA-cytosine methyltransferase
1998	Ethylene receptor 2
1712	F-box/LRR-repeat protein 13
1759	Filamentation induced by cAMP protein Fic
1920	Glycoprotein X precursor
1876	Guanosine polyphosphate pyrophosphohydrolase/synthetase
1869	Guanosine-3',5'-bis(diphosphate) 3'-pyrophosphohydrolase
1440	GTP pyrophosphokinase
2024	Heavy metal tolerance protein precursor
1771	Inversin
1718	Inversin-B
1842	Methionyl-tRNA synthetase
1988	methyltransferase, FkbM family
1932	Myosin heavy chain, non-muscle
1813	O-acetyl transferase
1971	peptide deformylase
1764	phenylalanine-4-hydroxylase
2019	Phenylserine dehydratase
1757	Poly-beta-hydroxybutyrate polymerase
1973	PREDICTED: retinoblastoma binding protein 9
1865	Putative hemagglutinin protein
1933	Quaternary ammonium compound-resistance protein sugE
1860	rare lipoprotein A precursor
1735	Restriction endonuclease S subunits
1804	Restriction modification system DNA specificity domain
1815	Ribosomal protein L11 methyltransferase
1776	Sulfatase
1767	Type I restriction enzyme EcoR124II M protein
1717	Type I restriction-modification system methyltransferase subunit
1976	Type I restriction-modification system methyltransferase subunit
1725	Type I site-specific restriction-modification system, R (restriction) subunit
1812	UvrABC system protein C
1833	probable ABC transporter nitrate permease
1907	ABC transporter ATP-binding protein Uup, erythromycin resistance
1818	ABC-type anion transport system permease component
1944	sodium/pantothenate symporter
1965	Citrate-proton symporter
1845	MFS type sugar transporter
1955	MFS type sugar transporter
2007	MFS type sugar transporter
1969	multidrug resistance protein B
1835	Reticulocyte-binding protein 1 precursor

1734	Outer membrane protein A precursor
1753	Ankyrin repeat
1772	Ankyrin repeat
1808	Ankyrin repeat
1875	Ankyrin repeat
1958	Ankyrin repeat
1962	Ankyrin repeat domain-containing protein 1
1939	Ankyrin repeat domain-containing protein 18A
1798	Ankyrin repeat domain-containing protein 28
1897	Ankyrin repeat domain-containing protein 36A
1979	Ankyrin repeat domain-containing protein 44
1830	Tetratricopeptide repeat-containing protein
1861	Tetratricopeptide repeat-containing protein
1926	Tetratricopeptide repeat-containing protein
1726	LexA repressor
1715	transcription regulator containing helix turn helix domain
1992	Cytotoxic translational repressor of toxin-antitoxin system RelE
2014	Transcriptional activator protein yukR
1828	transcriptional regulator, AbrB family
1896	transcriptional regulator, AbrB family
1800	Caspase recruitment domain-containing protein 15
1914	Caspase recruitment domain-containing protein 15
1982	Caspase recruitment domain-containing protein 15
2009	Caspase recruitment domain-containing protein 15
1820	Prophage antirepressor
7	Pilin gene-inverting protein
1791	type-F conjugative transfer system pilin assembly protein TrbC
1814	Conjugative transfer protein TraE
1741	F pilus assembly protein TraF
1792	F pilus assembly protein TraH
1748	TraU
1852	Conjugative transfer protein TraV
1849	type-F conjugative transfer system protein TraW
1870	F pilin acetylation protein TraX
1912	Chitinase
1858	Transposase and inactivated derivative
1942	Transposase and inactivated derivative
1850	similar to transposase
8	similar to transposase
1091	similar to transposase
935	similar to transposase
937	similar to transposase
942	similar to transposase
1868	conserved hypothetical protein
1946	conserved hypothetical protein
1827	Hypothetical protein, conserved

[1] Underscored RiOGs depict non-representative OGs.
[2] Including 222 representative HPs and two non-representative HPs.
