# Supplementary material for: Rickettsia Phylogenomics: Unwinding the Intricacies of Obligate Intracellular Life
Source: PLoS One. 2008 Apr 16;3(4):e2018. doi: 10.1371/journal.pone.0002018 (PMC2635572; doi:10.1371/journal.pone.0002018)
Supplement: Table S4 — OGs present only in the R. bellii genomes. (0.06 MB PDF) [file pone.0002018.s007.pdf]

Table S4. Distribution of putative toxin-antitoxin (TA) systems within the rickettsial OGs predicted by OrthoMCL*.

TA System	Toxin	RiOG	Distribution										Antitoxin	RiOG	Distribution									
		no.	Br	Bo	Ca	Pr	Ty	Ak	Fe	Ri	Co	Si		no.	Br	Bo	Ca	Pr	Ty	Ak	Fe	Ri	Co	Si
relBE	RelE	1992	1	1	0	0	0	0	0	0	0	0	RelB/DinJ	1153	0	0	0	0	0	0	1	1	1	1
		1251	1	1	0	0	0	1	1	0	0	0		1169	0	0	0	0	0	0	1	1	1	1
		3672	0	0	0	0	0	0	1	0	0	0	StbD	1273	0	0	0	0	0	0	1	1	0	1
		1262	1	1	0	0	0	1	1	0	0	0		1256	1	1	0	0	0	1	1	0	0	0
		3832	0	0	0	0	0	0	1	0	0	0		1118	0	0	0	0	0	1	1	1	1	1
	RelE/StbE	1560	0	0	0	0	0	0	2	0	0	0	StbD-like	977	1	1	0	0	0	1	1	1	1	1
		1487	0	0	1	0	0	1	1	0	0	0		2056	0	0	0	0	0	1	1	0	0	0
													Txe/YoeB	2069	0	0	0	0	0	1	1	0	0	0
														1864	0	0	0	0	0	0	1	0	0	0
														975	1	1	0	0	0	1	1	1	1	1
phd/doc																								
	Doc												Phd	1446	1	1	0	0	0	0	1	0	0	0
														1352	0	0	0	0	0	0	1	0	1	1
														965	1	1	0	0	0	1	1	1	1	1
														1084	1	1	0	0	0	0	0	1	1	1
														1427	1	1	0	0	0	0	1	0	0	0
														1408	1	1	0	0	0	0	1	0	0	0
														1470	1	1	0	0	0	1	0	0	0	0
														1037	1	1	0	0	0	1	0	1	1	1
														3220	0	0	0	0	0	0	1	0	0	0
vapBC/vag																								
	VapC	1036	1	1	0	0	0	1	0	1	1	1	VapB	941	1	1	1	0	0	0	1	1	1	1
		870	1	1	1	0	0	1	1	1	1	1												
	PDP	1435	1	1	0	0	0	0	1	0	0	0												
		1135	0	0	?	0	0	1	1	1	1	1												
		1004	1	1	0	0	0	0	1	1	1	1												
		2066	0	0	0	0	0	1	1	0	0	0												
		1466	1	1	0	0	0	1	0	0	0	0												
		1266	1	1	0	0	0	1	1	0	0	0												
mazEF																								
	mazF	1243	1	1	0	0	0	1	1	0	0	0												
	PemK-like	1087	1	1	0	0	0	0	0	1	1	1												
parDE																								
	ParE												ParD	1240	1	1	0	0	0	1	1	0	0	0

* Following the seven typical TA gene families categorized by Gerdes et al. (2005).
