# Supplementary material for: Rickettsia Phylogenomics: Unwinding the Intricacies of Obligate Intracellular Life
Source: PLoS One. 2008 Apr 16;3(4):e2018. doi: 10.1371/journal.pone.0002018 (PMC2635572; doi:10.1371/journal.pone.0002018)
Supplement: Table S5 — Distribution of putative toxin-antitoxin (TA) systems within the rickettsial OGs predicted by OrthoMCL. (0.07 MB PDF) [file pone.0002018.s008.pdf]

Table S5. Singletons present in the *R. bellii* str. RML369-C genome.

RiOG	Annotation (97)[1]		Size[2]
3504	ATP synthase subunit alpha		251
3066	F-box/LRR-repeat protein 13		279
2863	Fibroin heavy chain precursor		180
3167	GTP-binding protein		199
2378	small GTP-binding protein domain		216
2131	Modification methylase BsuRI		147
2881	NAD-specific glutamate dehydrogenase		140
3431	Osmolarity sensor protein EnvZ		175
3131	Probable inorganic polyphosphate/ATP-NAD kinase		65
3452	Protease 2		141
2180	recombination protein F		40
2621	recombination protein F		43
2300	Membrane-fusion protein component of the RND family transporter		82
2887	Proline/betaine transporter		265
2211	Outer membrane protein A precursor		143
3168	Outer membrane protein B precursor		75
2819	Cell surface antigen-like protein Sca11		53
3020	patatin-like protein		84
2213	Pilin gene-inverting protein		211
2209	similar to transposase		237
2747	similar to transposase		210
2838	similar to transposase		137
3531	similar to transposase		274
3105	Transposase		57
2264	conserved hypothetical protein		213
2629	conserved hypothetical protein		270
2811	Hypothetical protein, conserved		220
3065	Hypothetical protein, conserved		332
3280	Hypothetical protein, conserved		223
3426	Hypothetical protein, conserved		146
3517	Hypothetical protein, conserved		314
		**Avg.**	**174.9**

[1] Including 65 singleton HPs, with average length of 65.78 amino acids, and one false singleton HP.
[2] Length in amino acids of predicted ORF.
