# Supplementary material for: Rickettsia Phylogenomics: Unwinding the Intricacies of Obligate Intracellular Life
Source: PLoS One. 2008 Apr 16;3(4):e2018. doi: 10.1371/journal.pone.0002018 (PMC2635572; doi:10.1371/journal.pone.0002018)
Supplement: Table S6 — Singletons present in the R. bellii str. RML369-C genome. (0.05 MB PDF) [file pone.0002018.s009.pdf]

Table S6. Singletons and false singletons present in the *R. bellii* str. OSU 85 389 genome.

RiOG[1]	Annotation (112)[2]	Size[3]
3113	2,3,4,5-tetrahydropyridine-2,6-dicarboxylate N-succinyltransferase	51
3435	30S ribosomal protein S5	51
2418	Actin polymerization protein RickA	86
2972	Actin polymerization protein RickA	62
2953	Adenylate kinase	93
2548	Alanyl-tRNA synthetase	71
2493	Tyrosyl-tRNA synthetase	206
2396	Methionyl-tRNA formyltransferase	36
2704	Chaperone protein hscA homolog	198
2775	Filamentation induced by cAMP protein Fic	215
3332	Hemolysin C	136
2159	Lipoprotein releasing system, transmembrane protein	99
3174	Peroxisomal targeting signal receptor	367
3382	predicted amidophosphoribosyltransferase	34
2119	Putative hemagglutinin protein	112
3255	Ribonuclease HI	46
2253	RNA polymerase sigma factor rpoD	36
2405	RNA polymerase sigma factor rpoD	105
3087	Type I restriction enzyme EcoEI M protein	107
2345	Type I restriction-modification system, M subunit	72
2456	UbiD family decarboxylases	55
2387	Putrescine-ornithine antiporter	115
2847	Putrescine-ornithine antiporter	99
2170	Proline/betaine transporter	153
2311	Pilin gene-inverting protein	249
2755	Leucine-rich repeat protein	33
2654	Transposase and inactivated derivative	121
<u>3</u>	Transposase and inactivated derivative	NA
2759	conserved hypothetical protein	169
2764	Conserved hypothetical protein	115
2891	conserved hypothetical protein	118
3177	Conserved hypothetical protein	597
3219	conserved hypothetical protein	286
2269	Hypothetical protein, conserved	566
	**Avg.**	**147.24**

[1] Underscored RiOGs depict non-representative OGs.
[2] Including 79 singleton HPs, with average length of 76.70 amino acids, and four false singleton HPs.
[3] Length in amino acids of predicted singleton ORFs; lengths of false singletons not applicable.
