# Supplementary material for: Rickettsia Phylogenomics: Unwinding the Intricacies of Obligate Intracellular Life
Source: PLoS One. 2008 Apr 16;3(4):e2018. doi: 10.1371/journal.pone.0002018 (PMC2635572; doi:10.1371/journal.pone.0002018)
Supplement: Table S7 — Singletons and false singletons present in the R. bellii str. OSU 85 389 genome. (0.06 MB PDF) [file pone.0002018.s010.pdf]

Table S7. Singletons and false singletons present in the *R. canadensis* str. McKiel genome.

RiOG[1]	Annotation (175)[2]	Size[3]
3398	AAA+ superfamily protein	58
3400	Putative AAA+ superfamily ATPase	65
3541	Putative AAA+ superfamily ATPase	43
2613	putative ATPase	107
3428	acetoacetyl-CoA reductase	45
2337	ALANINE DEHYDROGENASE (pntAA)	39
2474	COG1012: NAD-dependent aldehyde dehydrogenases	97
2472	Retinal dehydrogenase 2	49
2335	NAD(P) transhydrogenase subunit alpha	40
3070	NAD(p) transhydrogenase subunit beta	44
3316	NAD(p) transhydrogenase subunit beta	68
2223	Nucleotidyltransferase	33
2191	Predicted glutamine amidotransferase	89
2566	Type I site-specific restriction-modification system, R (restriction) subunit	56
2389	ABC transporter ATP-binding protein Uup	61
3359	ABC transporter ATP-binding protein Uup	100
2532	ABC-type multidrug transport system, ATPase and permease components	36
2859	COG2984: ABC-type uncharacterized transport system, periplasmic component	42
3160	MFS type sugar transporter	53
3153	MFS-type transporter	54
2091	Proline/betaine transporter	49
2344	Variable membrane protein-like protein	46
3171	COG4953: Membrane carboxypeptidase/penicillin-binding protein PbpC	63
2255	COG2982: Uncharacterized protein involved in outer membrane biogenesis	87
3033	Outer membrane assembly protein	33
3311	Outer membrane protein B precursor	87
2645	Cell surface antigen-like protein Sca13	66
2685	Cell surface antigen-like protein Sca7	62
2722	Cell surface antigen-like protein Sca7	56
3178	Cell surface antigen-like protein Sca7	76
2219	Ankyrin	109
3463	Tetratricopeptide repeat-containing protein	57
2140	Conjugal transfer protein TraA	69
2141	Conjugal transfer protein TraA	84
2421	Conjugal transfer protein TraA	77
3034	Conjugal transfer protein TraA	72
3163	Conjugal transfer protein TraA	48
3278	Conjugal transfer protein TraA	60
3468	Conjugal transfer protein TraA	82
2965	Microcin C7 resistance protein	46
3286	Microcin C7 resistance protein	74
3418	Antigenic heat-stable 120 kDa protein	47
3395	NT (nucleotidyltransferase) domain and HEPN (higher eukarytoes and prokaryotes nucleotide-binding) domain	43
808	Transposase IS200-like	NA
2900	COG5304: Uncharacterized protein conserved in bacteria	61

3374	COG3177: Uncharacterized conserved protein	52
2597	COG4285: Uncharacterized conserved protein	39
	**Avg.**	**61.39**

[1] Underscored RiOGs depict non-representative OGs.
[2] Including 129 singleton HPs, with average length of 53.49 amino acids.
[3] Length in amino acids of predicted singleton ORFs.
