# Supplementary material for: Rickettsia Phylogenomics: Unwinding the Intricacies of Obligate Intracellular Life
Source: PLoS One. 2008 Apr 16;3(4):e2018. doi: 10.1371/journal.pone.0002018 (PMC2635572; doi:10.1371/journal.pone.0002018)
Supplement: Table S8 — Singletons and false singletons present in the R. canadensis str. McKiel genome. (0.06 MB PDF) [file pone.0002018.s011.pdf]

Table S8. Singletons present in the *R. prowazekii* str. Madrid E genome.

RiOG	Annotation (68)[1]	Size[2]
2320	190 kDa antigen precursor, putative	138
2664	190 kDa antigen precursor, putative	165
2818	Ankyrin Repeat family protein	107
3482	Antigenic heat-stable 120 kDa protein	247
2708	Lipid A export ATP-binding/permease protein msbA	121
2888	MFS type sugar transporter, putative	43
3485	putative methyltransferase	250
2309	tRNA pseudouridine synthase B	126
3481	ubiquinone biosynthesis protein, putative	96
2204	Hypothetical protein, conserved	91
2347	Hypothetical protein, conserved	60
2717	Hypothetical protein, conserved	67
	**Avg.**	**125.92**

[1] Including 56 singleton HPs, with average length of 63.11 amino acids.
[2] Length in amino acids of predicted ORF.
