# Supplementary material for: Rickettsia Phylogenomics: Unwinding the Intricacies of Obligate Intracellular Life
Source: PLoS One. 2008 Apr 16;3(4):e2018. doi: 10.1371/journal.pone.0002018 (PMC2635572; doi:10.1371/journal.pone.0002018)
Supplement: Table S9 — Singletons present in the R. prowazekii str. Madrid E genome. (0.05 MB PDF) [file pone.0002018.s012.pdf]

Table S9. Singletons present in the *R. typhi* str. Wilmington genome.

RiOG	Annotation (56)[1]		Size[2]
2639	CYTOCHROME C OXIDASE POLYPEPTIDE III (coxC)		38
3068	Hypothetical protein, conserved		42
2319	Ribonuclease PH		51
2615	SURFEIT LOCUS PROTEIN 1 (surf1)		58
		**Avg.**	**47.25**

[1] Including 52 singleton HPs, with average length of 42 amino acids, and one false singleton HP.
[2] Length in amino acids of predicted singleton ORFs; lengths of false singletons not applicable.
