# Supplementary material for: Rickettsia Phylogenomics: Unwinding the Intricacies of Obligate Intracellular Life
Source: PLoS One. 2008 Apr 16;3(4):e2018. doi: 10.1371/journal.pone.0002018 (PMC2635572; doi:10.1371/journal.pone.0002018)
Supplement: Table S10 — Singletons present in the R. typhi str. Wilmington genome. (0.05 MB PDF) [file pone.0002018.s013.pdf]

Table S10. Singletons and false singletons present in the *R. akari* str. Hartford genome.

RiOG[1]	Annotation (284)[2]	Size[3]
2382	Acetylglutamate kinase	53
2963	Acetylglutamate kinase	55
2853	Acetyltransferase	30
2451	alanine racemase	64
2919	alanine racemase	74
2246	ATP-binding cassette sub-family B member 7, mitochondrial	59
2610	ATP-binding cassette sub-family B member 7, mitochondrial	66
2663	ATP-binding cassette sub-family B member 7, mitochondrial precursor	73
3180	COG0350: Methylated DNA-protein cysteine methyltransferase	37
2158	COG0616: Periplasmic serine proteases (ClpP class)	50
2586	COG0616: Periplasmic serine proteases (ClpP class)	59
2851	COG1043: Acyl-	29
3089	D-alanyl-D-alanine dipeptidase	35
2598	D-beta-hydroxybutyrate dehydrogenase	58
2324	NADH dehydrogenase I chain H	56
2640	NADH dehydrogenase subunit N	99
2229	DNA methylase N-4/N-6	64
3505	DNA methylase N-4/N-6	41
3501	DNA modification methylase-like	44
3251	Formamidopyrimidine-DNA glycosylase	64
3318	Putative 3-methyladenine DNA glycosylase	50
2656	Glycosyltransferase	51
3078	Ribose-phosphate pyrophosphokinase	52
3288	GTP pyrophosphokinase	111
3289	GTP pyrophosphokinase	109
2517	Guanosine polyphosphate pyrophosphohydrolase/synthetase	44
3321	Guanosine polyphosphate pyrophosphohydrolase/synthetase homolog	57
3162	Guanosine-3',5'-bis(diphosphate) 3'-pyrophosphohydrolase	80
3432	Guanosine-3,5-bis(diphosphate) 3-pyrophosphohydrolase	61
2978	Predicted ATPase	45
3498	Pyrroloquinoline quinone (Coenzyme PQQ) biosynthesis protein C	60
2588	Magnesium and cobalt transport protein CorA	57
3204	Magnesium and cobalt transport protein CorA	37
2522	ABC transporter ATP-binding protein	71
2184	MFS type sugar transporter	61
2376	MFS type sugar transporter	31
2855	MFS type sugar transporter	185
2525	Na+/H+ antiporter NhaA	34
2220	Hydrophobe/amphiphile efflux-1 HAE1 family protein	61
2485	Hydrophobe/amphiphile efflux-1 HAE1 family protein	39
2605	Hydrophobe/amphiphile efflux-1 HAE1 family protein	106
3152	Hydrophobe/amphiphile efflux-1 HAE1 family protein	76
2650	Efflux transporter, RND family, MFP subunit	84
2458	RND family efflux transporter	94
3510	Transporter	102
3116	outer membrane protein A	32
2130	Outer membrane protein A precursor	163
2940	Outer membrane protein A precursor	67
2101	Cell surface antigen-like protein Sca10	128
2497	Cell surface antigen-like protein Sca10	45
2971	Cell surface antigen-like protein Sca10	52

3222	Cell surface antigen-like protein Sca10	38
2206	Cell surface antigen-like protein Sca7	75
2370	Cell surface antigen-like protein Sca7	72
2457	Cell surface antigen-like protein Sca7	69
3064	Cell surface antigen-like protein Sca7	73
2222	Cell surface antigen-like protein Sca8	71
2785	Cell surface antigen-like protein Sca8	103
2810	Cell surface antigen-like protein Sca8	53
2988	Multidrug resistance protein mdtB	110
2454	Microcin C7 resistance protein	42
2224	COG3210: Large exoproteins involved in heme utilization or adhesion	41
2183	invasion protein homolog	49
3184	Growth inhibitor	34
2287	Leucine-rich repeats (LRRs), ribonuclease inhibitor (RI)-like subfamily protein	38
2420	NT (nucleotidyltransferase) domain and HEPN (higher eukarytoes and prokaryotes nucleotide-binding) domain	81
3408	plasmid maintenance system antidote protein, XRE family	51
3543	Conjugal transfer protein TraD	45
2799	Phage portal protein	67
3296	Transposase	65
2738	Transposase and inactivated derivative	36
2556	transposase Tn5	41
2860	transposase Tn5	97
3069	transposase Tn5	49
2212	Transposase, IS982 family	73
<u>26</u>	Transposase, IS200 family protein	NA
2871	Conserved hypothetical protein	105
3366	Conserved hypothetical protein	189
2177	Hypothetical protein, conserved	38
2674	Hypothetical protein, conserved	35
2784	Hypothetical protein, conserved	52
2837	Hypothetical protein, conserved	33
3484	Hypothetical protein, conserved	46
	**Avg.**	**64.95**

[1] Underscored RiOGs depict non-representative OGs.
[2] Including 202 singleton HPs, with average length of 68.20 amino acids, and 2 false singleton HPs.
[3] Length in amino acids of predicted singleton ORFs; lengths of false singletons not applicable.
