# Supplementary material for: Rickettsia Phylogenomics: Unwinding the Intricacies of Obligate Intracellular Life
Source: PLoS One. 2008 Apr 16;3(4):e2018. doi: 10.1371/journal.pone.0002018 (PMC2635572; doi:10.1371/journal.pone.0002018)
Supplement: Table S11 — Singletons and false singletons present in the R. akari str. Hartford genome. (0.07 MB PDF) [file pone.0002018.s014.pdf]

Table S11. Singletons and false singletons present only in the *R. felis* genome.

RiOG[1]	Annotation (312)[2]	Size[3]
3322	Alanyl-tRNA synthetase	235
2601	Alpha-latrotoxin precursor	406
2406	ATP-dependent protease La	815
2831	ATP-dependent RNA helicase drs1	78
3536	biotin synthase	309
2230	Caspase recruitment domain-containing protein 15	234
2718	Cephalosporin hydroxylase	40
3238	Chaperone clpB	118
2291	COG0419: ATPase involved in DNA repair	54
1057	COG0847: DNA polymerase III, epsilon subunit and related 3'-5' exonucleases	NA
1601	Type I restriction-modification system methyltransferase subunit	NA
3173	COG0477: Permeases of the major facilitator superfamily	45
3258	COG0493: NADPH-dependent glutamate synthase beta chain and related oxidoreductases	38
2721	COG0605: Superoxide dismutase	43
2604	COG1752: Predicted esterase of the alpha-beta hydrolase superfamily	37
3161	D-beta-hydroxybutyrate dehydrogenase	46
2829	Dehydrogenase/reductase SDR family member 4	84
2643	Dimethyladenosine transferase	36
2202	Excinuclease ABC subunit C	100
2773	short chain dehydrogenase	124
3472	Thermostable carboxypeptidase 1	181
1596	Cell division cycle protein 27 homolog	NA
2952	Fic family protein	83
3387	Fic family protein	127
3478	Fic family protein	56
3216	gp84	92
2531	GTP pyrophosphokinase	140
2842	GTP pyrophosphokinase	172
3061	GTP pyrophosphokinase	204
2476	Putative GTP pyrophosphokinase	238
1595	thymidylate kinase	NA
3503	Leucine-rich repeats (LRRs), ribonuclease inhibitor (RI)-like subfamily protein	102
2100	metallophosphoesterase	253
3076	Mg chelatase-related protein	46
2894	Penicillin acylase precursor	377
2970	Citrate-proton symporter	425
2401	ABC transporter ATP-binding protein	274
2680	High-affinity choline transporter 1	984
2967	MFS-type transporter	397
2492	PROLINE/BETAIN TRANSPORTER (proP2)	79
2394	Proline/betaine transporter	307
3284	Proline/betaine transporter	276
2106	101 kDa malaria antigen	352
3050	Chitinase A precursor	442
1587	Patatin T5 precursor	NA
3082	Ankyrin repeat domain-containing protein 28	305
1597	Ankyrin repeat protein	NA
3352	Ankyrin-2	124

1586	Ankyrin-2	NA
2930	Chain A, Crystal Structure Of E3_19 An Designed Ankyrin Repeat Protein , protein related	181
2928	PREDICTED: similar to ankyrin 2,3/unc44	1179
2168	Outer membrane protein A precursor	103
2524	Outer membrane protein A precursor	333
3051	Outer membrane protein A precursor	476
3124	Outer membrane protein A precursor	105
3516	Outer membrane protein A precursor	28
3370	cell surface antigen Sca12	46
3457	cell surface antigen Sca12	77
3490	cell surface antigen Sca12	179
1594	Spore protein SP21	NA
1280	Spore protein SP21	NA
1579	COG3706: Response regulator containing a CheY-like receiver domain and a GGDEF domain	NA
2545	COG2944: Predicted transcriptional regulator	43
1283	COG1396: Predicted transcriptional regulators	NA
3048	addiction module toxin, Txe/YoeB family	86
2342	Cytotoxic translational repressor of toxin-antitoxin (TA) system RelE	92
1560	Addiction module toxin, RelE/StbE	NA
1610	ParA family protein	NA
2400	ParB-like partition proteins	252
2670	Plasmid stabilization system	90
2359	stability protein	211
1606	TraA	NA
1588	Conjugative transfer protein TraG	NA
2402	Rickettsial palindromic element (RPE) domain	54
2902	Rickettsial palindromic element (RPE) domain	52
2908	Rickettsial palindromic element (RPE) domain	41
3268	Rickettsial palindromic element (RPE) domain	39
3097	TPR repeat	213
3538	Transposable element Tc1 transposase	155
993	Insertion sequence IS5376 putative ATP-binding protein	NA
1281	putative transposase	NA
994	Putative transposase for insertion sequence element IS5376	NA
1578	transposase	NA
928	Probable transposase for transposon Tn903	NA
3146	Probable transposase for insertion sequence element	53
1602	ISBma2, transposase	NA
1585	Transposase for insertion sequence element IS1328	NA
1591	Transposase for insertion sequence element IS1328	NA
995	Transposase for insertion sequence element IS21	NA
1282	Transposon Tn917 resolvase	NA
2428	Trichohyalin	216
2630	Uncharacterized phage-associated protein	213
2096	Conserved hypothetical protein	219
2226	conserved hypothetical protein	131
2281	Conserved hypothetical protein	147
2427	Conserved hypothetical protein	247
2473	Conserved hypothetical protein	138
2515	Conserved hypothetical protein	139
2537	Conserved hypothetical protein	157
2584	Conserved hypothetical protein	282
2600	Conserved hypothetical protein	310
2638	Conserved hypothetical protein	234

2682	Conserved hypothetical protein	107
3091	Conserved hypothetical protein	273
3147	Conserved hypothetical protein	94
3210	Conserved hypothetical protein	94
3234	Conserved hypothetical protein	437
3394	Conserved hypothetical protein	195
3436	Conserved hypothetical protein	169
3544	Conserved hypothetical protein	136
1581	conserved hypothetical protein	NA
1583	Conserved hypothetical protein	NA
1589	Conserved hypothetical protein	NA
1603	Conserved hypothetical protein	NA
2248	Hypothetical protein, conserved	48
2399	Hypothetical protein, conserved	179
2540	Hypothetical protein, conserved	254
2549	Hypothetical protein, conserved	32
3403	Hypothetical protein, conserved	349
3439	Hypothetical protein, conserved	35
3345	protein of unknown function DUF1016	175
2873	predicted protein	146
3520	Orf12	82
	**Avg.**	**193.66**

[1] Underscored RiOGs depict non-representative OGs.
[2] Including 218 singleton HPs, with average length of 68.20 amino acids, and 25 false singleton HPs.
[3] Length in amino acids of predicted singleton ORFs; lengths of false singletons not applicable.
