# Supplementary material for: Rickettsia Phylogenomics: Unwinding the Intricacies of Obligate Intracellular Life
Source: PLoS One. 2008 Apr 16;3(4):e2018. doi: 10.1371/journal.pone.0002018 (PMC2635572; doi:10.1371/journal.pone.0002018)
Supplement: Table S12 — Singletons and false singletons present only in the R. felis genome. (0.08 MB PDF) [file pone.0002018.s015.pdf]

Table S12. Singletons present in the *R. rickettsii* str. Sheila Smith CWPP genome.

RiOG	Annotation (153)[1]		Size[2]
2252	30S ribosomal protein S1		149
2288	AAA+ superfamily protein		64
2898	ABC-type multidrug transport system, ATPase and permease components		76
2709	Acetoacetyl-CoA reductase		53
2236	Ankyrin repeat		31
2668	Ankyrin repeat		49
3057	Ankyrin repeat		88
2128	Conserved hypothetical protein		105
3420	Conserved hypothetical protein		85
3380	Dihydrofolate reductase FolA		45
2681	Hypothetical protein, conserved		66
3334	Hypothetical protein, conserved		39
2426	methylated-DNA-[protein]-cysteine S-methyltransferase		52
3393	MFS type sugar transporter		58
2251	plasmid maintenance system antidote protein		46
3200	Poly-beta-hydroxyalkanoate depolymerase		36
3304	Prophage antirepressor		46
3088	Pyrroloquinoline quinone (Coenzyme PQQ) biosynthesis protein C		37
3299	Rickettsial palindromic element (RPE) domain		50
2395	S-adenosylmethionine synthetase		64
3125	similarity to methylated-DNA--protein-cysteine methyltransferase		41
3326	sodium/pantothenate symporter		67
2612	Superfamily I DNA and RNA helicase		97
2921	Vegetative cell wall protein gp1 precursor		63
		**Avg.**	**62.79**

[1] Including 129 singleton HPs, with average length of 52.02 amino acids.
[2] Length in amino acids of predicted ORF.
