# Supplementary material for: Rickettsia Phylogenomics: Unwinding the Intricacies of Obligate Intracellular Life
Source: PLoS One. 2008 Apr 16;3(4):e2018. doi: 10.1371/journal.pone.0002018 (PMC2635572; doi:10.1371/journal.pone.0002018)
Supplement: Table S13 — (0.06 MB PDF) [file pone.0002018.s016.pdf]

Table S13. Singletons present in the *R. conorii* str. Malish 7 genome.

RiOG	Annotation (97)[1]	Size[2]
2768	3-hydroxyacyl-CoA dehydrogenase FadB	70
3139	AAA+ superfamily protein	58
2797	ABC-type multidrug transport system, ATPase and permease components	90
3223	Acetate kinase	83
2984	Ankyrin repeat	87
2675	COG0419: ATPase involved in DNA repair	82
2310	COG0457: FOG: TPR repeat	40
3241	COG1835: Predicted acyltransferases	97
2553	COG3243: Poly(3-hydroxyalkanoate) synthetase	87
2284	COG5430: Uncharacterized secreted protein	63
2511	Hypothetical protein, conserved	40
3488	Hypothetical protein, conserved	40
2429	Mg chelatase-related protein	67
3083	Proline/betaine transporter	77
2813	Prolyl endopeptidase precursor	53
2383	Putative multidrug resistance protein 22	131
3199	Rickettsial palindromic element (RPE) domain	36
3496	RND efflux system, outer membrane protein	59
2833	Tellurite resistance protein-related protein	46
2589	Type I restriction-modification system methyltransferase subunit	58
	**Avg.**	**68.2**

[1] Including 77 singleton HPs, with average length of 53.84 amino acids.
[2] Length in amino acids of predicted ORF.
