# Supplementary material for: Rickettsia Phylogenomics: Unwinding the Intricacies of Obligate Intracellular Life
Source: PLoS One. 2008 Apr 16;3(4):e2018. doi: 10.1371/journal.pone.0002018 (PMC2635572; doi:10.1371/journal.pone.0002018)
Supplement: Table S14 — (0.05 MB PDF) [file pone.0002018.s017.pdf]

Table S14. Singletons present in the *R. sibirica* str. 246 genome.

RiOG	Annotation (114)[1]		Size[2]
2241	Acetyltransferase		26
2487	COG0666: FOG: Ankyrin repeat		75
2440	COG1835: Predicted acyltransferases		82
2772	ComEC/Rec2-related protein		43
3486	Competence protein F		38
2279	Glycosyltransferase		34
2365	Heme exporter protein A		34
2776	Lipid A export ATP-binding/permease protein msbA		65
2261	Os02g0189700		79
2181	Periplasmic divalent cation tolerance protein		43
2285	Probable tRNA-dihydrouridine synthase		82
3126	Proline/betaine transporter		41
3134	Rickettsial palindromic element (RPE) domain		48
3437	S-adenosylmethionine synthetase		69
2419	similarity to cell surface antigen		113
2243	similarity to methylated-DNA--protein-cysteine methyltransferase		46
2305	similarity to type I restriction enzyme restriction subunit		45
2918	Vegetative cell wall protein gp1 precursor		59
2137	Hypothetical protein, conserved		58
2780	Hypothetical protein, conserved		36
3534	Hypothetical protein, conserved		74
		**Avg.**	**56.67**

[1] Including 93 singleton HPs, with average length of 45.06 amino acids, and one false singleton.
[2] Length in amino acids of predicted singleton ORFs; lengths of false singletons not applicable.
