# Supplementary material for: Rickettsia Phylogenomics: Unwinding the Intricacies of Obligate Intracellular Life
Source: PLoS One. 2008 Apr 16;3(4):e2018. doi: 10.1371/journal.pone.0002018 (PMC2635572; doi:10.1371/journal.pone.0002018)
Supplement: Table S15 — (0.05 MB PDF) [file pone.0002018.s018.pdf]

Table S15. Sequences retrieved from GenBank for *R. helvetica* and *R. australis* used in the phylogenetic analyses shown in Figure 9 and Figure S13.

Gene	RiOG	*R. helvetica*	*R. australis*
citrate synthase	175	Q59741	P51039
co-chaperonin GroES	335	ABD93985	NA
chaperonin GroEL	336	ABD93984	NA
VirB4 protein precursor	225	ABG74480	ABG74462
recombinase A	245	ABG74458	ABG74444
molecular chaperone DnaK	667	ABG74418	ABG74428
Chromosomal replication initiator protein DnaA	356	ABG74394	ABG74398
ATP synthase alpha subunit	208	AAM93518	ABG74366
190 KD antigen precursor (sca1)	797	AAU06440	AAU06437
DNA polymerase III subunit alpha	230	CAB56077	NA
DNA polymerase I	231	Q9RLB6	NA
Cell division protein FTSY	232	CAB56072	NA
Cell surface antigen Sca4	432	AAL23857	Q9AJ64
DNA-directed RNA polymerase beta subunit	701	AAM93506	NA
Elongation factor EF-Tu	305	Q8KT99	NA
Elongation factor EF-G	708	Q8KTB4	NA
